# Comparative evolutionary diversity and phylogenetic structure across multiple forest dynamics plots: a mega-phylogeny approach

**DOI:** 10.3389/fgene.2014.00358

**Published:** 2014-11-05

**Authors:** David L. Erickson, Frank A. Jones, Nathan G. Swenson, Nancai Pei, Norman A. Bourg, Wenna Chen, Stuart J. Davies, Xue-jun Ge, Zhanqing Hao, Robert W. Howe, Chun-Lin Huang, Andrew J. Larson, Shawn K. Y. Lum, James A. Lutz, Keping Ma, Madhava Meegaskumbura, Xiangcheng Mi, John D. Parker, I. Fang-Sun, S. Joseph Wright, Amy T. Wolf, W. Ye, Dingliang Xing, Jess K. Zimmerman, W. John Kress

**Affiliations:** ^1^Department of Botany, Museum Routing Code-166, National Museum of Natural History, Smithsonian InstitutionWashington, DC, USA; ^2^Department of Botany and Plant Pathology, Oregon State UniversityCorvallis, OR, USA; ^3^Smithsonian Tropical Research Institute, PanamáPanamá; ^4^Department of Plant Biology, Michigan State UniversityEast Lansing, MI, USA; ^5^Forest Ecosystem Station of the Pearl River Delta, State Forestry Administration, Research Institute of Tropical Forestry, Chinese Academy of ForestryGuangzhou, China; ^6^Conservation Ecology Center, Smithsonian Conservation Biology Institute, Smithsonian InstitutionFront Royal, VA, USA; ^7^Key Laboratory of Plant Resources Conservation and Sustainable Utilization, South China Botanical Garden, The Chinese Academy of SciencesGuangzhou, China; ^8^Center for Tropical Forest Science-Forest Global Earth Observatory, Smithsonian Tropical Research InstituteWashington, DC, USA; ^9^State Key Laboratory of Forest and Soil Ecology, Institute of Applied Ecology, Chinese Academy of ScienceShenyang, China; ^10^Department of Natural and Applied Sciences, Biology Program, University of Wisconsin-Green BayGreen Bay, WI, USA; ^11^Laboratory of Molecular Phylogenetics, Department of Biology, National Museum of Natural ScienceTaichung, Taiwan; ^12^Department of Forest Management, The University of MontanaMissoula, MT, USA; ^13^National Institute of Education, Nanyang Technological University, SingaporeSingapore; ^14^Wildland Resources, Utah State UniversityLogan, UT, USA; ^15^State Key Laboratory of Vegetation and Environmental Change, Institute of Botany, Chinese Academy of SciencesBeijing, China; ^16^Department of Zoology, Faculty of Science, University of PeradeniyaPeradeniya, Sri Lanka; ^17^Smithsonian Environmental Research Center, Smithsonian InstitutionEdgewater, MD, USA; ^18^Department of Natural Resources and Environmental Studies, National Dong Hwa UniversityHualien, Taiwan; ^19^Institute for Tropical Ecosystem Studies, University of Puerto RicoSan Juan, PR, USA

**Keywords:** ForestGEO, barcode, phylogeny, community assembly, phylogenetic diversity, ecology

## Abstract

Forest dynamics plots, which now span longitudes, latitudes, and habitat types across the globe, offer unparalleled insights into the ecological and evolutionary processes that determine how species are assembled into communities. Understanding phylogenetic relationships among species in a community has become an important component of assessing assembly processes. However, the application of evolutionary information to questions in community ecology has been limited in large part by the lack of accurate estimates of phylogenetic relationships among individual species found within communities, and is particularly limiting in comparisons between communities. Therefore, streamlining and maximizing the information content of these community phylogenies is a priority. To test the viability and advantage of a multi-community phylogeny, we constructed a multi-plot mega-phylogeny of 1347 species of trees across 15 forest dynamics plots in the ForestGEO network using DNA barcode sequence data (*rbc*L, *mat*K, and *psb*A-*trn*H) and compared community phylogenies for each individual plot with respect to support for topology and branch lengths, which affect evolutionary inference of community processes. The levels of taxonomic differentiation across the phylogeny were examined by quantifying the frequency of resolved nodes throughout. In addition, three phylogenetic distance (PD) metrics that are commonly used to infer assembly processes were estimated for each plot [PD, Mean Phylogenetic Distance (MPD), and Mean Nearest Taxon Distance (MNTD)]. Lastly, we examine the partitioning of phylogenetic diversity among community plots through quantification of inter-community MPD and MNTD. Overall, evolutionary relationships were highly resolved across the DNA barcode-based mega-phylogeny, and phylogenetic resolution for each community plot was improved when estimated within the context of the mega-phylogeny. Likewise, when compared with phylogenies for individual plots, estimates of phylogenetic diversity in the mega-phylogeny were more consistent, thereby removing a potential source of bias at the plot-level, and demonstrating the value of assessing phylogenetic relationships simultaneously within a mega-phylogeny. An unexpected result of the comparisons among plots based on the mega-phylogeny was that the communities in the ForestGEO plots in general appear to be assemblages of more closely related species than expected by chance, and that differentiation among communities is very low, suggesting deep floristic connections among communities and new avenues for future analyses in community ecology.

## Introduction

Phylogenetic hypotheses have played an increasingly important role in ecology over the last decade and their use in understanding community processes has been well reviewed (Webb et al., 2002; Cavender-Bares et al., 2009; Swenson, 2013). Knowledge of phylogenetic relationships among species has been used to quantify various aspects of ecology, including competition (Webb, 2000; Kembel and Hubbell, 2006; Webb et al., 2008; Cavender-Bares et al., 2009; Lebrija-Trejos et al., 2013), environmental filtering (Cavender-Bares et al., 2004; Uriarte et al., 2010; Liu et al., 2013; Pearse et al., 2013), pathogen and herbivore selection (Gilbert and Webb, 2007; Whitfeld et al., 2012), succession (Whitfeld et al., 2012) and the spatial differentiation of phylogenetic diversity (Weiblen et al., 2006; Graham and Fine, 2008; Fine and Kembel, 2011). In the context of conservation biology, phylogenetic information has also been used to quantify diversity within and among communities (Faith, 1992; Hardy and Senterre, 2007). The best measure of diversity that is most relevant for conservation assessment remains an important question. For example, does species diversity or phylogenetic diversity best capture the full spectrum of organismal diversity and traits in a community or habitat to be conserved (e.g., Swenson, 2013)? Nonetheless, the ability of phylogenetic data to precisely quantify evolutionary history within and among communities provides a framework for addressing how best to quantify, manage and conserve biodiversity and communities.

The application of evolutionary information to questions in community ecology has been limited in large part by the lack of accurate estimates of phylogenetic relationships among individual species found within communities. This dearth of information has been particularly true for the most species- and ecologically-diverse communities in the tropics where existing phylogenetic data are most limiting (Webb and Donoghue, 2005; Kress et al., 2009). Traditionally, phylogenetic systematists have focused on taxonomic groups and lineages, not communities, on the assumption that phylogenetic treatments are most robust when all members of a clade are included in the analysis. In communities where diverse sets of species are present, the very large evolutionary divergences among co-occurring taxa and more sparse taxonomic sampling have been thought to hinder accurate reconstructions of phylogenetic relationships (Poe and Swofford, 1999).

Newly emerging tools for constructing community phylogenies have largely ameliorated these concerns. Supertree methods, which prune and graft taxa from existing phylogenetic trees, can be used to construct phylogenetic relationships among species in a community (Bininda-Emonds and Sanderson, 2001; Webb and Donoghue, 2005). However, these methods have two drawbacks. Firstly, a phylogeny assembled from separate phylogenetic trees carries topological information, but contain no information on the evolutionary distances connecting species (i.e., branch lengths). Because the use of phylogenies in community ecology is specifically dependent upon evolutionary distances, branch lengths must be inferred. Assigning branch lengths to a topology with no intrinsic branch length information requires assumptions (e.g., bladj; Webb et al., 2008) where the branch lengths between any two dated nodes are evenly divided among the nodes separating the dates, which is unrealistic. Secondly, unless the reference trees from which the super-phylogeny is constructed contain all members of the community, which is extremely unlikely particularly for diverse tropical communities, the relationships of many species will be inferred only at higher taxonomic levels where relationships are completely resolved (Kress et al., 2009) and information about the tips of the phylogeny will be lost. Despite these limitations supertree-based community phylogenies have in many ways revolutionized community ecology. The availability of supertree tools, such as phylomatic (Webb and Donoghue, 2005), has resulted in an explosion of interest in the merging of community ecology and phylogenetic systematics (Swenson, 2013).

A relatively new source of phylogenetic character information available to complement supertree methods in community ecology is DNA barcode sequence data. Multi-locus DNA barcodes for plants are composed of genes or parts of genes that have traditionally been used in molecular systematics (Soltis et al., 2011). The community phylogenies that have been estimated from DNA barcode sequence data are robust and congruent with overall phylogenetic expectations for vascular plants (Kress et al., 2009; Pei et al., 2011; Whitfeld et al., 2012; Yessoufou et al., 2013). The advantage of these DNA barcode phylogenies is their ability to (1) better resolve relationships at the species-level in clades where supertree methods are less robust and (2) provide direct estimates of evolutionary distances (e.g., branch lengths) that connect clades within the phylogeny (Kress et al., 2009).

Recently supertree methods have been combined with DNA barcode sequence data to enhance resolution in community phylogenies (e.g., Kress et al., 2010). In these cases the phylogenetic relationships generated through supertree algorithms are a combination of broadly accepted patterns of taxonomic relationships at the deepest phylogenetic nodes provided by a guide or constraint tree while phylogenetic resolution among genera and species at the tips of the branches is provided by the rapidly evolving DNA barcode markers. Equally important is that branch lengths may be estimated with the DNA barcode sequence data throughout the tree, including the parts of the tree that are constrained. This merging of the two methods has been particularly fruitful in a number of community studies (e.g., Kress et al., 2010; Uriarte et al., 2010; Lebrija-Trejos et al., 2013).

The next step in community analyses is to build multiple local phylogenies simultaneously that can be quantitatively compared. Currently most community phylogenies are constructed for one community at a time using different genes and different algorithms for estimating the phylogeny, as well as employing different dating methods, all of which will likely limit the ability to compare results among the communities. A few studies have employed molecular phylogenies to multiple communities (Swenson, 2012), but most comparisons among communities have relied upon either species taxonomic lists (Ricklefs et al., 2012) or taxonomic supertree methods (e.g., phylomatic). If we are to use phylogenetics to compare the structure, diversity, and ecological determinants of diversity among communities, then we must develop robust methods to build and employ multi-community phylogenies. Furthermore, an area in which the application of phylogenetic hypotheses to understanding ecological processes remains relatively less well explored is the geographic distribution of phylogenetic diversity and structure (Hardy and Jost, 2008). The power of sequence-based phylogenies to resolve evolutionary relationships and calculate evolutionary distances within communities can now be applied to determining genetic differentiation and phylogenetic diversity among sites and communities by combining DNA barcode sequence data from multiple communities into a mega-phylogeny across these communities. The value of using these measures of phylogenetic diversity to assess the conservation status of communities representing various habitat types and regions across the globe should not be underestimated (e.g., Faith, 1992).

In this study the ForestGEO (http://www.forestgeo.si.edu) global network of forest dynamics plots was used as the focus for developing a single large phylogeny for comparing measures of phylogenetic structure within and among plots. These plots have been developed over the last three decades to monitor forest change in different forest types around the world. Recently an effort has been initiated to generate DNA barcodes for tree species in each plot as a new tool for forensic ecology and community phylogenetics (e.g., Kress et al., 2009, 2010; Jones et al., 2011; Pei et al., 2011; Swenson, 2012). Here a method is developed for reconstructing species relationships based on the DNA barcode sequence data in fifteen different ForestGEO plots simultaneously by constructing a single mega-phylogeny. The benefits of a simultaneous phylogenetic reconstruction are addressed by estimating branch lengths and evolutionary divergence within and among the individual plots. Finally, analyses of the geographic distribution of community structure, measures of phylogenetic diversity across these plots (e.g., Phylogenetic Diversity, Mean Phylogenetic Diversity, and Mean Nearest Taxon Density), and inferences into the mechanisms that produce these observed patterns are provided.

## Materials and methods

### Community sampling and genotyping

**Figure 1:**
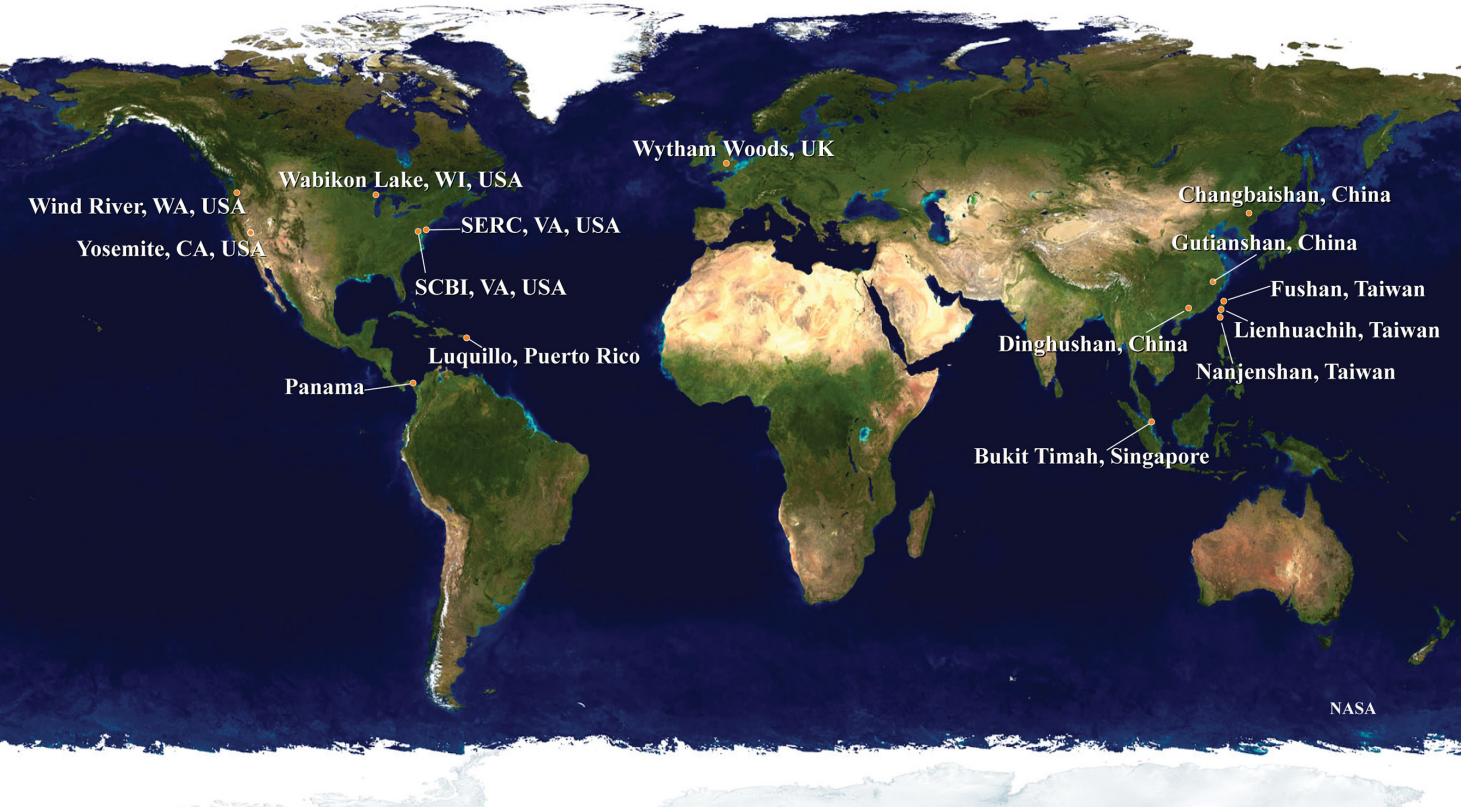
**The distribution of the 15 ForestGEO plots incorporated into the mega-phylogeny are shown**. The plots encompass temperate, sub-tropical and tropical habitats and are distributed globally.

The samples for our analyses were obtained from 15 forest dynamics plots, which are part of the ForestGEO network organized by the Smithsonian Institution (http://www.forestgeo.si.edu; Figure 1). Some of these sites have been the focus of investigations into the application of DNA barcodes in understanding the processes of community ecology (e.g., Kress et al., 2009, 2010; Uriarte et al., 2010; Pei et al., 2011; Swenson, 2012). We used samples from four plots in tropical Asia, two from sub-tropical Asia, one from temperate Asia, two from the neotropics, five from temperate North America, and one from temperate Europe (Table 1). A total of 1347 species were included in the final dataset, encompassing 553 genera in 125 families and 43 orders.

**Table 1:** **Descriptions of the ForestGEO plots examined in this study are given**.

**Plot**	**Species**	**Genera**	**Families**	**Geography**	**Habitat**	**Coordinates**
BCI	337	205	55	New-world	Tropics	8.63, −77.81
Bukit-Timah	326	177	61	Asian	Tropics	3.37, 98.92
Dinghushan	192	114	20	Asian	Sub-tropics	23.30, 114.54
Gutianshan	146	97	44	Asian	Sub-tropics	28.04, 121.08
Luquillo	141	107	39	New-world	Tropics	17.61, −67.68
Lienhuachih	129	79	49	Asian	Tropics	25.44, 120.27
Fushan	98	62	30	Asian	Tropics	24.21, 123.59
SCBI	62	37	52	New-world	Temperate	38.89, −78.14
Changbaishan	54	35	17	Asian	Temperate	42.38, 128. 08
Nanjenshan	42	36	17	Asian	Tropics	22.070, 122.73
Waibikon lake	30	23	18	New-world	Temperate	45.551, −88.78
SERC	28	20	15	New-world	Temperate	38.89, −76.56
Wytham	18	12	5	Europe	Temperate	51.77, −1.338
Wind river	7	4	3	New-world	Temperate	45.82, −121.95
Yosemite	7	5	4	New-world	Temperate	37.77, −119.82
Mega-phylogeny	1347	553	125			

*For each plot, the number of species, genera, and families is shown, as are general classification of the Geography, habitat type, and GPS coordinates. The number of species in the Mega-phylogeny is given, and is smaller than the sum among all communities due to shared species in some communities*.

Three samples per species were directly sequenced at three separate loci corresponding to the commonly used DNA barcode markers: (1) 552 bp of the ribulose-bisphosphate/carboxylase Large-subunit gene (*rbc*L;), (2) approximately 760 bp of the maturase-K gene (*mat*K), and (3) the *psb*A-*trn*H intergenic spacer (median 450 bp). All three markers are derived from the chloroplast genome. Methods for DNA extraction, PCR, and sequencing follow Kress et al. (2009) and Pei et al. (2011). Sequences for some of taxa were retrieved from GenBank (trees in Yosemite, Wind-River, and Wytham plots); for an individual species we used only our original sequence data or GenBank data and never combined original DNA barcode sequence data with GenBank data for the same species. All DNA barcode data generated for the study have been submitted to GenBank (see Supplemental Table S1 for accession numbers for our original sequences and those retrieved from GenBank).

### Sequence alignment

DNA barcode sequence data for trees collected from the 15 forest dynamics plots at each of the three separate markers were aligned across all species then concatenated together in an alignment supermatrix for estimation of phylogenetic relationships. The *rbc*L gene data were aligned through back-translation, using transAlign (Bininda-Emonds, 2005). The *mat*K gene was also initially aligned using transAlign, and then adjusted manually to remove gaps corresponding to frame-shift mutations. Following manual adjustment of the alignment to remove gaps, the matrix was aligned a second time using MAFFT (Katoh and Standley, 2013), implementing the FFT-NS-2 option for larger datasets. The *psb*A-*trn*H marker was aligned using SATe (Liu et al., 2012), implementing the PRANK aligner (Löytynoja and Goldman, 2005) for sub-groupings and the MUSCLE aligner (Edgar, 2004) for merging sub-alignments. SATe is a “divide and conquer” style algorithm where an initial set of sequences is subdivided into smaller sets which are aligned and then joined back into a single alignment using a consensus alignment algorithm. SATe is iterative and goes through many cycles of generating sub-alignments and merging to consensus alignment using the likelihood score of a phylogenetic tree to determine an optimal alignment state. To improve the estimate of alignment in SATe, a guide tree derived from the Phylomatic portal (Webb and Donoghue, 2005) was used as a starting tree in the alignment. The guide tree used in SATe was not a constraint tree, and thus the tree inferred from a final alignment in SATe may differ from the phylomatic input tree. SATe allowed us to generate a single alignment block for the hyper-variable *psb*A-*trn*H marker for all species, in contrast to sets of nested alignments as used previously (Kress et al., 2009).

### Phylogenetic reconstruction

The aligned 3-gene matrix was fully analyzed in the phylogenetic tree-building algorithm GARLI (Zwickl, 2006) via the CIPRES portal (Miller et al., 2010) to produce the 1347 taxon phylogeny that we call the “mega-phylogeny.” The configuration file used with GARLI is given in Supplemental Table S2. In addition to the aligned 3-gene matrix we utilized a phylogenetic constraint tree (described below). The aligned data-file was also partitioned by locus for use in GARLI, so that each of the three genes had separate model parameters estimated using the program MODELTEST 3.7 (Posada and Crandall, 1998). The use of SATe greatly assisted model estimation at this stage because only a single model was required for the *psb*A-*trn*H marker, whereas with nested alignments either a single model would need to be chosen for all discrete alignment blocks (which would be artificial since the same model would not readily be chosen for all alignment partitions), or a very large number of models would be estimated separately for the same genetic locus. For a best tree search, 100 search replicates were initiated, each starting from random tree, to search for a best, most likely phylogeny. Further, we implemented a separate set of 100 bootstrap runs under the CAT-GAMMA model in GARLI, while still using the ordinal level constraint tree, to quantify support for the topology used in subsequent analyses.

Because of the relatively rapidly evolving sequence data provided by the DNA barcode markers and the inclusion of a large number of species spanning broad evolutionary distances, we employed a constraint tree to fix the deep phylogenetic relationships (Kress et al., 2010). The search for the best tree was performed with a constraint tree derived from Phylomatic using the R20120829 phylogenetic tree for plants, derived from the Angiosperm Phylogeny Group III reconstruction (APGIII, 2009). The constraint was modified in Mesquite (Maddison and Maddison, 2014) in which each taxonomic order was reduced to a polytomy. This effect enforced phylogenetic relationships at the level of order and above. The molecular data were then responsible for reconstructing family, generic, and species relationships within orders. The quality of the phylogenetic reconstructions was evaluated by quantifying the fraction of resolved nodes, and the level of monophyly at the taxonomic family- and genus-levels. Although the constraint tree fixed relationships among orders according to APGIII, the branch lengths for all groups of taxa, including those fixed by the constraint-tree, were calculated from the aligned DNA barcode sequence alignment. As such, the combination of the constraint and sequences enabled phylogeny reconstruction by limiting the searched tree space and estimation of branch lengths across the depth of the tree.

In addition to constructing a single phylogeny for 15 ForestGEO community plots, phylogenetic relationships were estimated in each of the 15 plots separately. Taxa corresponding to each plot were pruned out from the aligned 3-marker matrix produced for the full 1347 taxon set and a phylogeny was constructed using the alignment for the taxa present in each plot as described above. Any benefits of high-taxon density to sequence alignment in the larger dataset were accordingly propagated to the estimates of alignment for each individual plot. For each of the 15 community plots, a best tree search with 100 independent search replicates was conducted in GARLI via the CIPRES portal using the same configuration parameters as the mega-phylogeny. The best scoring ML tree was used in subsequent comparisons between individually constructed community phylogeny and those estimated within the context of the mega-phylogeny.

To evaluate how well taxa were resolved in the mega-phylogeny and in individually constructed plot phylogenies, the fraction of non-zero length branches (that is, the fraction of resolved branches) were calculated for the entire mega-phylogeny, for individual plots that were pruned out of the mega-phylogeny, and for each individually constructed plot phylogeny. To compare how changes in taxonomic composition were associated with degree of phylogenetic resolution, spearman rank correlation was computed between the resolution of each phylogeny with species richness, Mean Phylogenetic Distance (MPD) and Mean Nearest Taxon Distance (MNTD), the latter described below. Similarly, we used spearman correlation to examine how rates of resolution changed as a function of latitude, as we moved from the tropics to temperate environments.

### Mean path length (MPL) calibration of phylogeny

Mean Path Length (MPL) calibration (Britton et al., 2002) was used to transform all molecular phylogenies into ultrametric chronogram. MPL estimates branch lengths using the mean of all branches descending from it, and thus is closer to molecular clock calibration. The algorithm was implemented using APE (Paradis et al., 2004) implemented through the Picante package (Kembel et al., 2010) of the R programming language (R Core Team, 2012) with the “chonoMPL” command, setting the root age to 1, as opposed to attempting to assign any dates. This method was selected because (1) it most directly reflects inferred evolutionary distances (i.e., branch lengths) with the minimum of alteration of branch length relative to other methods of generating an ultrametric tree (Britton et al., 2002), and (2) attempts to use Bayesian methods for branch length calibration (e.g., BEAST; Drummond and Rambaut, 2007) were unable to reach a state where the optimization converged for the larger phylogenies. Thus, each of the 15 separately generated community phylogeny, and the mega-phylogeny were transformed with MPL and these transformed phylogenies were used in analysis of phylogenetic distance (PD) and diversity (Sections Phylogenetic Diversity Metrics and Comparative Community Phylogenetic Diversity and Structure).

### Phylogenetic diversity metrics

Three common metrics of phylogenetic diversity were utilized to quantify differences among the 15 ForestGEO plot-based community phylogenies. All of these metrics were estimated within the Picante package (Kembel et al., 2010) of the R programming language. For each plot community, the phylogenetic diversity was calculated and then the values observed were compared for individually constructed phylogenies and for those estimated within the mega-phylogeny. The PD metric (Faith, 1992), which sums the branch lengths for any defined set of taxa in a phylogeny, is correlated with species richness, but greatly refines estimates of diversity by incorporating a quantitative measure of evolutionary divergence (Faith, 1992; Forest et al., 2007; Morlon et al., 2011). For individually constructed community phylogenies, PD was simply the sum of all branch lengths in the phylogeny. For community phylogenies within the mega-phylogeny, PD was the sum of all branch lengths within the mega-phylogeny connecting the species belonging to that community.

The second metric utilized was MPD (Webb et al., 2002), which obtains an average for the pair-wise PD across all pairs of taxa in a community. As such, MPD is not directly correlated with species number by default, and is strongly influenced by branch lengths at the deepest nodes of the phylogeny (Swenson, 2013). This metric gives an estimate of the overall divergence of taxonomic clades present in a community and is sensitive to replacement of taxa that differ in broad taxonomic placement.

The third metric employed was MNTD (Webb et al., 2002), which provides an average of the distances between each species and its nearest phylogenetic neighbor in the community. MNTD quantifies the degree that a community may be a set of closely related species vs. a heterogeneous set of taxa from disparate taxonomic clades. MNTD is necessarily sensitive to replacement of closely related taxa and is much less sensitive to changes at the basal (or oldest) nodes of the phylogeny. For each of these terms, the phylogenetic diversity is inferred through the summed branch length distances connecting species in the phylogeny, thus distance is equivalent to diversity.

The absolute values of PD, MPD, and MNTD are not relevant here; rather the differences in these metrics estimated from independently derived phylogenies vs. those estimated from the mega-phylogeny are most important. To compare how estimates of phylogenetic diversity vary, the proportional difference for the values in each community were measured and values of difference were plotted for all 15-plot communities. For each metric, 15 values were calculated representing the difference between individually constructed plot phylogeny and values inferred from the mega-phylogeny. The percentage difference was calculated as: [(M_i_ − M_j_)/M_j_]^*^100 where M = the metric under evaluation (PD, MPD, or MNTD), i = the value estimated from individually constructed community phylogeny and j = the value estimated from the mega-phylogeny. A value of zero corresponds to no difference in estimates of PD between that inferred in the mega-phylogeny and that from individually constructed phylogenies. We further examined if there was a significant correlation between latitude and phylogenetic diversity using the spearman correlation coefficient with decimal values of latitude for each community plot. Whereas species richness is known to exhibit a strong latitudinal gradient, we used this correlation to evaluate if phylogenetic diversity metrics exhibit similar patterns.

### Comparative community phylogenetic diversity and structure

To compare the phylogenetic diversity and structure among ForestGEO plots, two methods were used, both estimated within the Picante package of the R programming language, and using the MPL transformed mega-phylogeny. The first metric was the Inter-community Mean Pairwise Distance, which is a measure of phylogenetic beta diversity (Webb et al., 2002) and is calculated as the mean for all pair-wise comparisons of PD between the taxa of two different communities (the “mpd.comdist” routine within Picante). The second metric is the MNTD among nearest-neighbor pairs of species in different communities (the “comdistnt” routine within Picante) and is sensitive to higher-level taxonomic substitutions (i.e., changes in representation of taxonomic family or order) among communities. For mpd.comdist and comdistnt, both the mean and variance of the inter-community PDs were plotted.

To further test if each of the 15 ForestGEO plots was a random sample of the larger community of species represented by the mega-phylogeny, a randomization test implemented in Picante was used to estimate the standard effects size of each of the three PD metrics. This test was run for the three phylogenetic diversity metrics PD, MPD, and MNTD using the MPL transformed mega-phylogeny. For each of the three metrics, the algorithm in Picante was run using 999 randomizations of the community within the mega-phylogeny applying the “taxa.labels.” The “taxa.labels” model maintains the species richness of each community as well as the number of forest plots a particular species may be assigned to (i.e., a species observed in one forest can only be found in one forest in the randomized data), but alters the evolutionary relationships (i.e., branch lengths connecting species) in that community by randomizing the names of the species at the tip of the phylogenetic tree (Webb et al., 2002). The model generates a distribution from the 999 independent randomizations, against which the observed value of phylogenetic diversity (PD, MPD, or MNTD) may then be compared and a *p*-value assigned to it. Communities with a *p*-value of <0.05 were judged to be significantly different from random within the context of the 15 plot mega-phylogeny. *Z*-values, observed and expected values of diversity, and *p*-value are given as supplemental data (Supplemental Tables S3–S5, respectively, for PD, MTD, and MNTD). Departures from random have been interpreted as a signal for local-level processes within communities, such that species with observed PDs significantly less than the randomized mean are more closely related than expected (i.e., phylogenetically clustered) and hence the result of environmental filtering on phylogenetically structured traits (Webb, 2000). Alternatively, species with evolutionary distances significantly greater than the observed mean are more distantly related than expected (i.e., phylogenetically overdispersed), which is consistent with the role of competition in structuring species composition (Webb et al., 2002). The entire ForestGEO mega-phylogeny was treated in essence as a global “meta-community” and as such these metrics provide evidence for similar ecological processes among communities that are linked to the environment or taxonomic structure.

## Results

### Phylogenetic reconstruction

**Figure 2:**
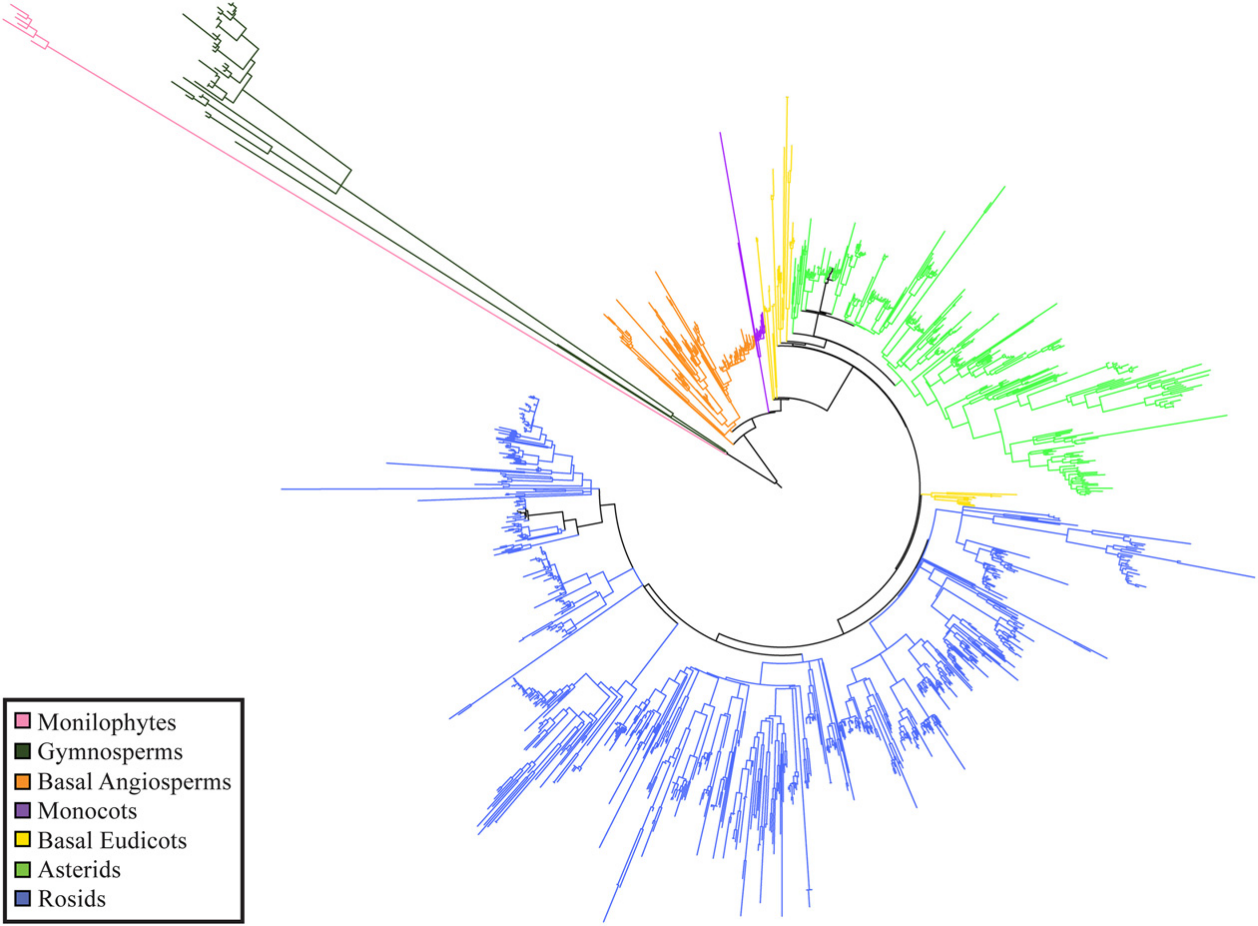
**Representation of the ForestGEO 15-plot mega-phylogeny, reconstructed with Maximum-Likelihood, shown with un-transformed branch lengths**.

Phylogenetic resolution, which is the fraction of non-zero length branches in a phylogeny, varied among the 15 single-plot phylogenies and the 15-plot mega-phylogeny. The 15-plot mega-phylogeny with molecular branch lengths selected from the most likely of 100 independent maximum-likelihood tree searches is shown in Figure 2. The distribution of the Orders throughout the 15-plot mega-phylogeny are presented in Figure 3A; with the diversity of orders within each plot shown in Figure 3B. The fraction of resolved species for the mega-phylogeny was over 78% using the phylogeny with the best likelihood score derived from 100 independent search replicates. A consensus tree from rapid bootstrapping of the mega-phylogeny found 70.2% of all nodes were supported using majority rule 50% criterion, which closely mirrored the 78% resolution in the highest scoring ML tree. The rates of resolution for the independently derived community phylogenies (Table 2) ranged from 81% (Dinghushan) to 100% (Wytham and Yosemite). A significant relationship was found between phylogenetic resolution and species richness (*r* = −0.799, *p* > 0.001), as smaller community phylogenies (and those at higher latitudes) were more likely to be fully resolved. Importantly, however, phylogenetic resolution for a plot was consistently higher when estimated within the context of the mega-phylogeny (Table 2). On average a 3.5% increase in resolution was found, ranging from an 8% increase for Bukit-Timah and Changbaishan to no increase for Wind-River and Yosemite (Table 2).

**Figure 3A:**
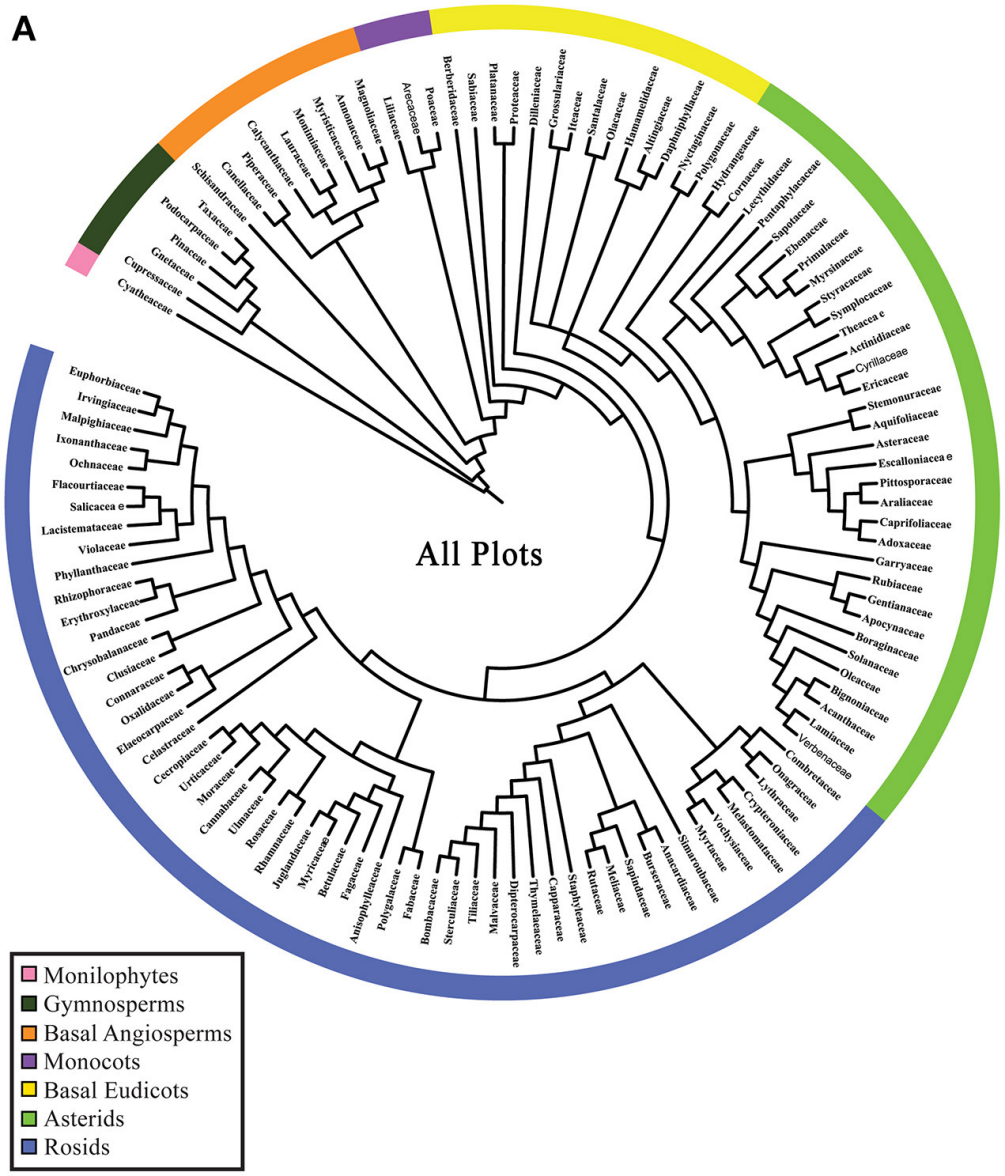
**Phylogenetic relationships of taxa in the 15 ForestGEO plots as a mega-phylogeny and as separate plots resolved at the level of taxonomic family**. A cladogram of the ForestGEO 15-plot mega-phylogeny, with 1347 taxa derived from molecular data is presented. Seven separate major phylogenetic groups of vascular plants are indicated to demonstrate the evolutionary diversity of species included in the mega-phylogeny. The composition of the mega-phylogeny is broadly congruent with land plant relationships showing high diversity in the Asterid, Rosid, and Basal Eudicot clades, and very low diversity among Monilophytes and Gymnosperm clades.

**Figure 3B:**
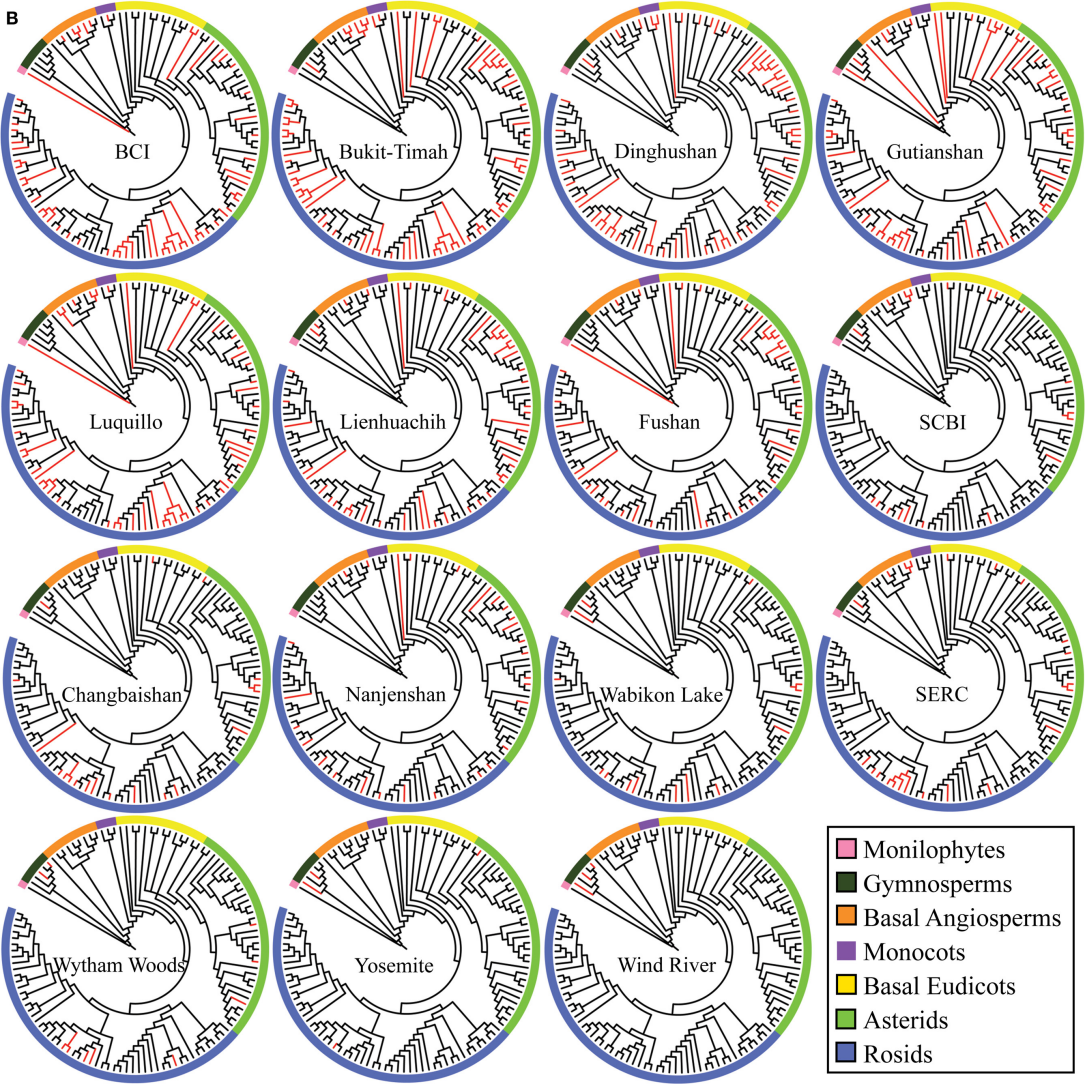
**Individual cladograms for each of the 15 separate ForestGEO plots arranged by species richness**. The families that are present in each individual plot are mapped on the mega-phylogeny in red to show the evolutionary and taxonomic diversity present in each plot.

**Table 2:** **Fraction of resolved nodes within the ForestGEO15 mega-phylogeny and each of the individual plots when estimated separately**.

**Plot**	**# Taxa**	**Individually constructed**	**Mega-phylogeny**	**Difference**
ForestGEO15	1347	n/a	0.78	n/a
BCI	337	0.89	0.93	0.04
Bukit-Timah	326	0.86	0.94	0.08
Dinghushan	192	0.81	0.85	0.04
Gutianshan	146	0.87	0.93	0.06
Luquillo	141	0.95	0.97	0.02
Lienhuachih	129	0.88	0.92	0.04
Fushan	98	0.89	0.91	0.02
SCBI	62	0.89	0.94	0.05
Changbaishan	54	0.85	0.93	0.08
Nanjenshan	42	0.95	0.96	0.01
SERC	30	0.92	0.97	0.05
Wabikon lake	28	0.95	0.98	0.03
Wytham	18	1	1	0
Wind river	7	1	1	0
Yosemite	7	1	1	0

*The fraction of non-zero length nodes in the phylogeny was used to determine the percent resolution for the best-supported ML phylogeny*.

A significant relationship was found between MNTD for a plot and its phylogenetic resolution (*r* = 0.874; *p* > 0.001), with higher MNTD equating to improved resolution. A similar effect was seen with MPD (*r* = 0.658; *p* = 0.008). The relationship of MNTD with phylogenetic resolution paralleled the observation of species richness and phylogenetic resolution, and was similar to correlation with latitude (*r* = 0.397, *p* = 0.142), such that as communities were composed of fewer species, it was easier to distinguish among them topologically.

### Community phylogenetic diversity and structure

**Figure 4:**
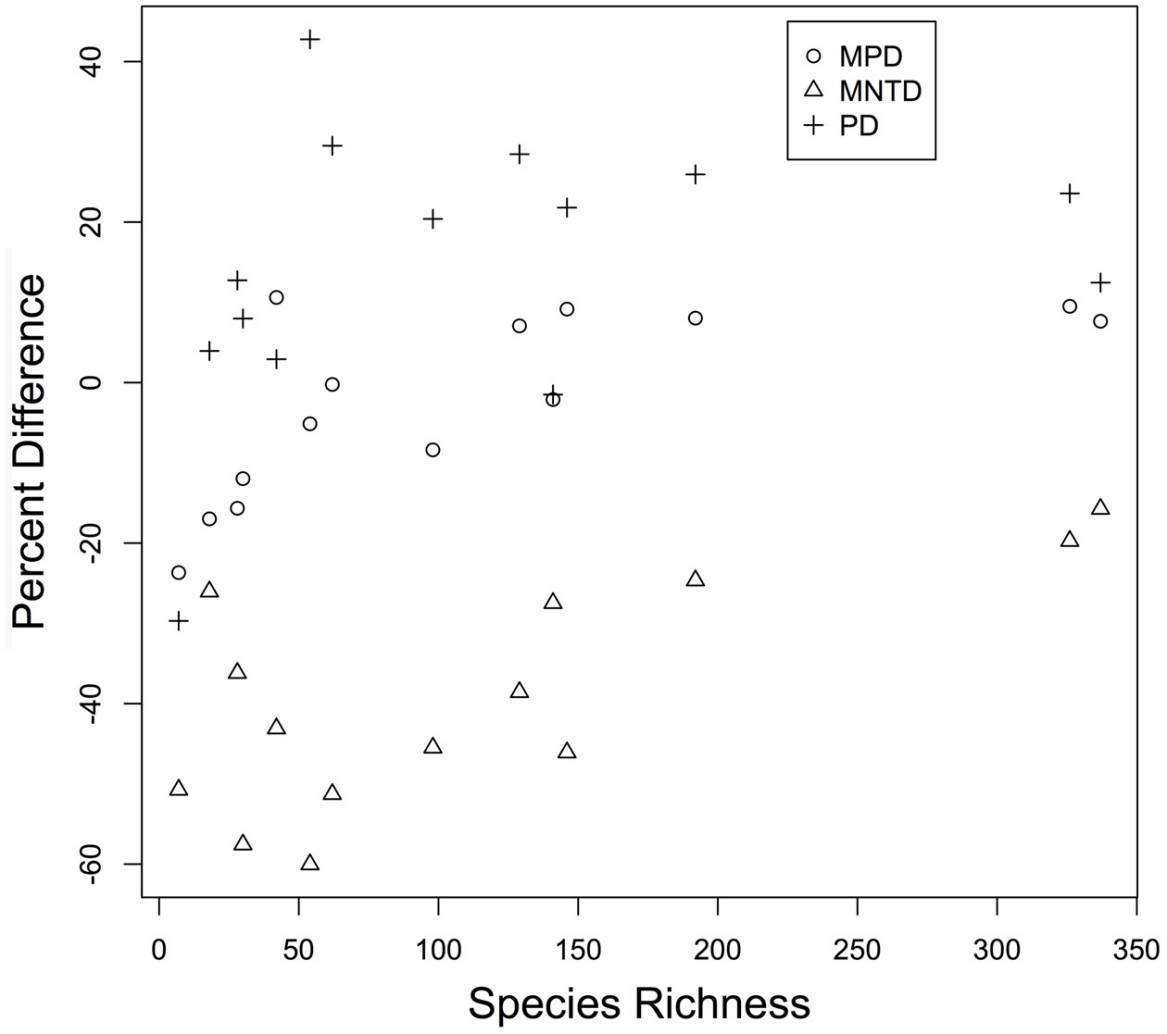
**The percentage difference in observed value of PD, MNTD, and MPD are plotted for each community**. Each point is the percent difference in the value of a metric calculated from individually constructed community phylogeny vs. that observed for the same community in the mega-phylogeny. Values are plotted as a function of Species Richness of the ForestGEO community.

**Figure 5:**
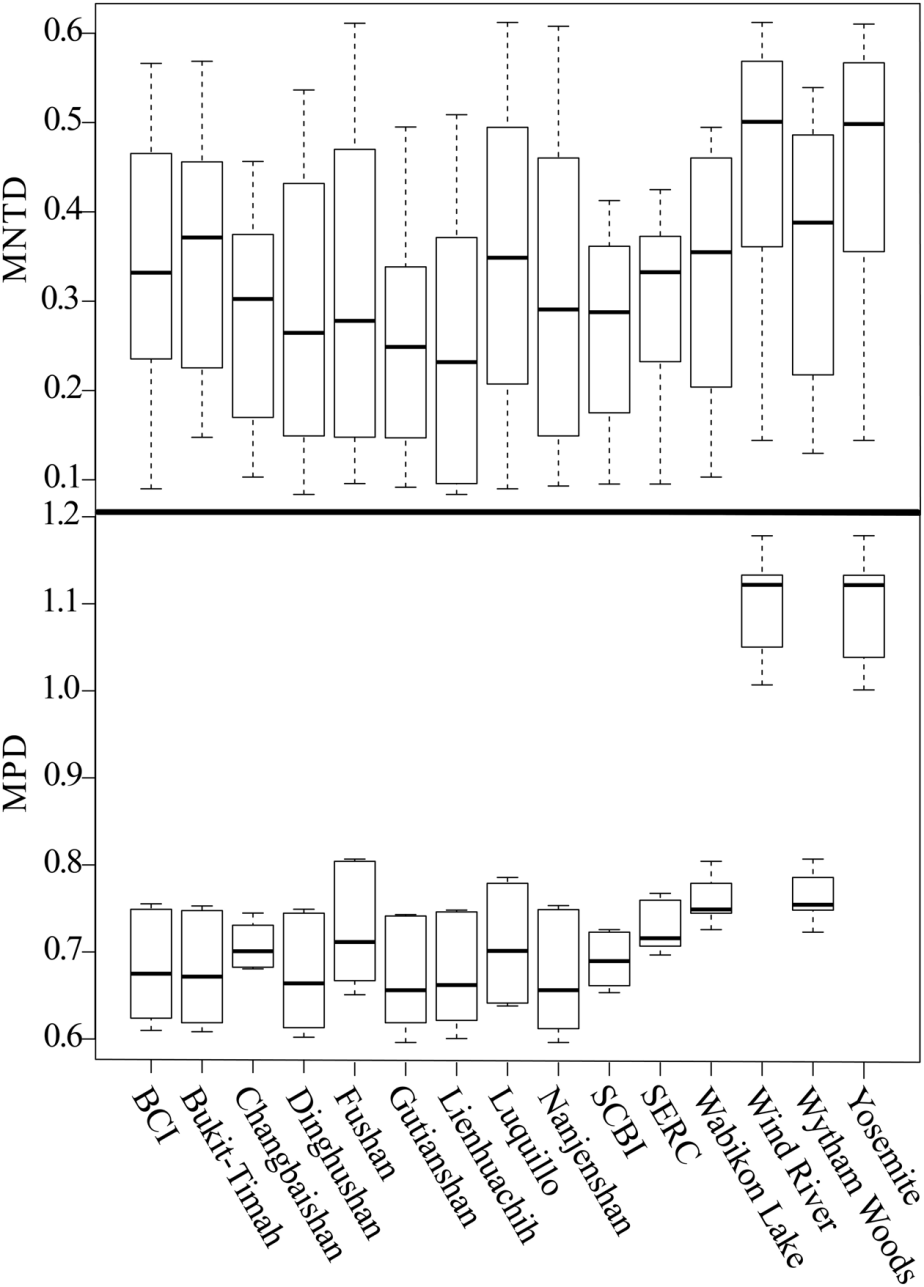
**Two methods to infer differentiation among communities are shown, with the inter-community MNTD (top) and inter-community MPD (bottom)**. Boxplots for each community show the mean (dark bar within box), interquartile range (box), and 95% confidence interval (whisker bars), computed from all pairwise contrasts between plots.

The three diversity metrics (PD, MPD, and MNTD) calculated for each plot varied for those derived from the mega-phylogeny vs. the individually constructed plot phylogenies (Figure 4). A weak relationship was observed between species richness and the proportional difference for PD (*r* = 0.393, *p* = 0.083), but exhibited a significant positive relationship for MPD (*r* = 0.741, *p* = 0.002) and MNTD (*r* = 0.525, *p* = 0.028) as larger plots exhibited less differentiation in the estimated metrics (Figure 4). Averaged over all communities, the percent difference in estimated PD was, PD = 14.38%, MPD = 2.297%, and MNTD = 38.76%. The percent difference for MNTD was striking, and is most evident in the smallest plots with a range of 60% divergence for Changbaishan, to 15% divergence for BCI (Figure 4), which reflects the difficulty that phylogenetic reconstruction methods may have in inferring evolutionary distances when the mean of those distances is very large. The improvements in estimates of PD within the mega-phylogeny are most dramatic for the smallest plots where the higher taxon density of the mega-phylogeny greatly improves estimates of branch lengths among all species found in those communities. The inter-plot Mean Phylogenetic Distance (inter-MPD) was broadly similar for 13 of the 15 plots (Figure 5), with only the most species poor plots (e.g., Wind-River and Yosemite) differing significantly from the other 13 plots. This reflects the wide taxonomic composition of many of the plots, where high variation within plots obscures differentiation among the plots, as seen through taxonomic representation of different orders within each plot (Figure 3B). Similarly, the inter-plot Mean Nearest Taxon Distance (inter-MNTD) exhibited no differentiation among any of the ForestGEO plots, regardless of geographic location or species richness (Figure 4).

In contrast to the inter-community diversity metrics, randomization tests, which evaluate if communities are a random subsample of the larger phylogeny, found that the communities were not a random set of species (Table 3). In the three PD metrics used, all three exhibited significant differences from random in the most speciose plots, with a consistent trend toward their being significantly clustered (Table 3, and Supplemental Tables S3–S5 for PD, MPD, and MNTD, respectively). For PD, the five temperate sites exhibited no departure from random, whereas each of the plots with more than 62 species (excepting Luquillo) was significantly clustered. For MNTD the result was even more skewed with 12 of the 15 plots exhibiting significant clustering. For MPD significant clustering was found for the four most species rich tropical plots (BCI, Bukit-Timah, Dinghushan, and Gutianshan), whereas the most species-poor community plots were inferred to be overdispersed (Wabikon Lake, Wind River, Wytham, and Yosemite). Overall the eight tropical or sub-tropical plots, when considered over all three PD metrics, were significantly clustered in 15 out of 24 cases. In the remaining nine cases they were not different from random, and none were inferred to be over-dispersed. Alternatively for the seven species-poor temperate plots, four were overdispersed (only with MPD), eight were significantly clustered (seven for MNTD and one for PD with Changbaishan), and the remaining 12 showed no departure from random (Table 3). Two plots, Luquillo and Nanjenshan, were consistent in exhibiting no significant departures from random for any of the phylogenetic diversity metrics whereas all other of the plot phylogenies exhibited some significant departure from random for at least one of the metrics.

**Table 3:** **Values for three species richness (SR) and three Phylogenetic Diversity metrics Phylogenetic Distance (PD), Mean Phylogenetic Distance (MPD), and Mean Nearest Taxon Distance (MNTD) are given for each plot**.

**Plot**	**SR**	**PD**	**MPD**	**MNTD**
BCI	337	**28.88** 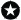	**0.61** 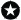	0.09
Bukit-Timah	326	**25.80** 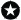	**0.60** 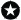	**0.08** 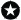
Dinghushan	192	**18.55** 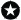	**0.72** 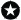	**0.09** 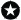
Gutianshan	146	**16.1** 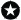	**0.60** 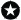	**0.12** 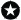
Luquillo	141	**19.57** 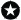	0.67	0.14
Lienhuachih	129	**14.17** 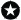	0.62	**0.11** 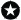
Fushan	98	**12.67** 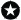	0.86	**0.12** 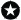
SCBI	62	**8.67** 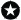	0.61	**0.13** 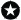
Changbaishan	54	**7.15** 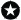	0.69	**0.10** 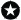
Nanjenshan	42	8.27	0.59	0.23
SERC	30	6.19	0.66	**0.19** 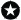
Wabikon lake	28	5.63	**0.75** 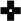	**0.18** 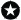
Wytham	18	4.38	**0.78** 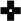	**0.17** 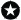
Wind river	7	3.13	**0.92** 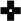	**0.31** 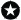
Yosemite	7	3.00	**0.79** 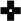	**0.31** 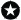

*For each metric 999 randomizations were used to assess departure from random community structure. Significant differences from random are in bold, with pattern denoted by superscript. Standard effect sizes, Z and p-values are reported in Supplemental Tables S3–S5*.

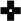
, *Significant Overdispersion;* 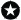, *Significant Clustering*.

## Discussion

In the field of ecology phylogenetic data have been used to understand ecological processes (Webb et al., 2002; Cavender-Bares et al., 2009), the roles of trait conservatism and dispersal limitation in structuring communities (Fine and Kembel, 2011; Liu et al., 2013), and the regulation of beta diversity (Swenson, 2012). In addition, phylogenetic information has been applied to the identification of specific environments critical for conservation (Faith, 1992; Forest et al., 2007; Morlon et al., 2011). Accordingly, the ability to generate and use phylogenetic data to address core questions in ecology and to assess conservation priorities are of increasing importance.

The results shown here demonstrate that constructing a single mega-phylogeny inclusive of many individual community plots improves the estimation of the evolutionary relationships and distances among species in each separate plot. The mega-phylogeny is also helpful in examining the patterns of phylogenetic diversity within and among plots to explore broad scale patterns that may reflect processes regulating community assembly and the maintenance of diversity. Long-term biodiversity monitoring plots, such as the ForestGEO network, provide an ideal context for investigating phylogenetic diversity and geographic structuring among plots to address questions regarding community assembly at very broad scales.

### Generating phylogenies

The use of a constraint tree to construct the mega-phylogeny was adopted in this study and it is recommended for use in large community phylogenies, particularly those built with rapidly evolving sequence data as found in DNA barcodes (Kress et al., 2010). For example, the non-protein coding marker *psb*A*-trn*H has been used phylogenetically at very low taxonomic scales (e.g., within genera or families) because of the difficulty in aligning sequences among distantly related taxa. This limitation has slowed its adoption as an official DNA barcode marker (Hollingsworth et al., 2011). However, in this study we were able to use the SATe algorithm to align *psb*A-t*rn*H across all species, including distantly related ones, in the analysis rather than as in prior studies in which the marker was aligned in a nested format within a supermatrix and did not contribute to the inferred relationships of deeper taxonomic scales (Kress et al., 2009; Pei et al., 2011). This marker evolves very rapidly and global alignment may have contributed to the non-constrained mega-phylogeny exhibiting differentiation from expectations in APGIII. However, the use of *psb*A*-trn*H in a global alignment produced a higher fraction of resolved nodes than the use of only *rbc*L+*mat*K, and did not negatively affect rates of family and generic monophyly (Table 1). Also, a nested approach to alignment of *psb*A-*trn*H requires some subjective decisions with regards to the scale at which to group sequences, which may result in the exclusion of sequences from taxa that are not readily included in groupings. This effect in turn will result in a greater asymmetry in the aligned sequence matrix, and, therefore, will complicate model selection for different data partitions in phylogenetic inference. For these reasons we recommend a global alignment of *psb*A-*trn*H in plant DNA barcode phylogenies using SATe in conjunction with a constraint tree that will enforce higher-level taxonomic resolutions.

Even the relatively limited sequence content from DNA barcode markers, as demonstrated here, can be successfully used to the construct a highly robust phylogeny across multiple plots with high rates of resolution and monophyly. When compared with other studies of very large phylogenies, the mega-phylogeny had comparable rates of resolution among species (Smith et al., 2009, 2011), and an overall remarkably high rate of 78% taxonomic resolution. The 15-plot mega-phylogeny with 1347 species in 43 orders and 125 families (Table 1, Figure 2) was significantly larger than the individual plots in which the average was 12 orders and 38 families (Table 1). The mega-phylogeny improved resolution among species in most communities relative to constructing phylogenies for individual plots (Table 2). The construction of a community phylogeny is greatly improved in the context of resolving difficult taxonomic relationships when taxon density is high (Smith et al., 2011) and the lower level of taxonomic resolution in the mega-phylogeny as a whole does not affect the inferred rates of resolution for the included plots. The increased taxon density of the mega-phylogeny represented by a lower estimate of the MNTD was a central driver in improving rates of phylogenetic resolution (see Supplemental Table S4). As the genetic distances among species become more continuous and evenly distributed, the ability to infer phylogenetic relationships increases, which is reflected in the strong correlation between decreasing MPD and increasing phylogenetic resolution (0.73). Therefore, as ever-larger mega-phylogenies are generated to include an expanded scope of land plant diversity, then more fully resolved and well-supported community phylogenies can be pruned from them.

### Improving phylogenetic resolution

Improving the accuracy of relationships among species in a community phylogeny is not just a methodological detail. Poorly resolved phylogenies can result in biased estimates of the diversity metrics used to infer ecological process (Davies et al., 2012) or may lead to very different conclusions about ecological process in a particular community (Kress et al., 2009). The low rates of taxonomic resolution in supertrees relative to molecular derived community phylogenies may adversely affect ecological inference (Kress et al., 2009); yet with supertrees, at least all samples in a study are assembled and dated similarly, and thus results observed among communities are consistent and comparable (Fine and Kembel, 2011). The challenge of collecting genetic data for all the members of a community has limited the use of molecular phylogeny in studies of community ecology, particularly in studies comparing across multiple communities (Swenson, 2012). With the widespread generation of DNA barcode data across tropical plots, such as the ForestGEO network of forest dynamics plots, information on phylogenetic relationships can now be applied to many communities simultaneously. The benefits of constructing phylogenies for multiple communities concurrently as well as the advantages of increased taxonomic resolution and more accurate evolutionary distances among species and clades are many. Because evolutionary distance, or branch lengths, are necessary to infer processes of community assembly, one of our goals was to quantify the improvement of estimating evolutionary distances through the use of a mega-phylogeny of many plots to construct phylogenies of individual plots.

Nearly all studies of community phylogenetics have examined one community at a time. In most cases the community phylogenies were constructed using supertree methods, including phylomatic (Webb, 2000; Cavender-Bares et al., 2004; Fine and Kembel, 2011) or direct sequence data (Kress et al., 2009; Uriarte et al., 2010; Pei et al., 2011), but it is difficult to know if differences in the results are attributable to differences in the phylogeny employed or in the ecological processes themselves. We have shown here that constructing a molecular phylogeny for all communities together improves estimates of phylogenetic diversity and structure compared to estimating individual phylogenies for each community.

### Phylogenetic diversity

A mega-phylogeny may also improve estimates of community phylogenetic diversity through the conversion of all phylogenies into molecular-clock-based ultrametric trees using the MPL adjustment (Britton et al., 2002) and then directly estimating three commonly employed diversity metrics (Table 3). Communities with the lowest species diversity showed the greatest contrast in diversity measures when estimated in the mega-phylogeny vs. the individual-plot phylogenies (Figure 4). For example, in the Yosemite and Wind-River plots (where species richness = 7), diversity estimates from individually-derived phylogenies were less than half that observed in the mega-phylogeny; whereas for the larger plots the differences were much less. For all communities, the values of PD were lower in individually-constructed community phylogenies (Figure 4). We note that this result considers only trees, and that work comparing canopy and understory diversity suggest that temperate forests may contain comparable phylogenetic diversity when all plants are considered (Halpern and Lutz, 2013). However, for our observations, divergence between estimates were correlated with species richness of the plot (Species Richness vs. % difference in MPD = 0.68) with smaller plots showing the greatest differentiation, and suggests that the mega-phylogeny should greatly improve comparisons among plots, particularly when those communities differ in species richness.

### Phylogenetic structure among communities

A growing, but still small, number of studies have compared phylogenetic structure across communities (Hardy et al., 2012; Swenson, 2012; Oliveira-Filho et al., 2013a,b). However, as shown here the evolutionary structure among plots, via the inter-community measures of MPD and MNTD (Figure 5), complements similar patterns of phylogenetic structure within communities. The lack of differentiation among plots (Figure 5), with the exception of the extremely taxon-poor Yosemite and Wind-River plots in the Cascade and Sierra Nevada Mountains, is striking. The prevalence of trees in the families Fabaceae, Euphorbiaceae, and Myrtaceae in the tropical plots and their relative paucity in the plots located in temperate environments was not significant enough to differentiate these communities in most cases. The effect of latitude on measures of phylogenetic diversity was highly significant (with PD, MPD, and MNTD showing Spearman correlation coefficient of -0.905, 0.684, 0.521, respectively) and followed changes in species richness along the tropical to temperate transition. The correlation for PD was negative with latitude, whereas MPD and MNTD were positive, reflecting how the two latter metrics remove the effect of species richness on phylogenetic diversity. The reliance of MPD on the genetic distances of the most basal nodes of the phylogeny and the emphasis on the presence or absence of basal lineages suggest that substitution of one family (or order) in communities that differ in species number are equivalent. It is even more striking that the inter-community estimates of MNTD should show similarly low rates of differentiation among sites. While the differentiation in MPD can be more readily explained by the role of deeper nodes in determining differentiation, the MNTD would be inflated when comparing environments from the tropics with that of the temperate zones. The lack of differentiation among plots corresponds well to the observation that trees in these plots are in general phylogenetically clustered, and that environmental filtering is driving assembly processes. The main caveat is that we can infer a role of environmental filtering from phylogenetic clustering only when the traits that drive fitness are evolutionarily conserved.

### Phylogenetic distance and ecological processes

A central benefit of constructing a mega-phylogeny containing many communities is our ability to more accurately contrast ecological processes operating in different communities. Therefore, phylogenetic patterns that are observed (e.g., clustering, overdispersion) are not attributable to differences in how community phylogeny are assembled, but are more directly linked to different ecological processes in those communities. We note that disentangling these processes within a community phylogenetic context remains a challenge, as we are just beginning to apply phylogenetic information to multiple communities and appropriate null models of phylogenetic pattern that incorporate explicit geographic differentiation are still being developed. The role of dispersal limitation and biogeographic vicariance in generating differences in species composition observed in different communities affect our results as would community assembly processes within sites. Yet the patterns derived with existing models can at least be viewed as having an ecological or evolutionary basis rather than a simple product of phylogeny construction.

In our study, for each of the different metrics of PD the most diverse tropical communities were composed of a set of more closely related species than expected at random in the context of the null model used (Table 3). The pattern of increased relatedness was most evident for the nearest-taxon metric MNTD, which exhibited significant clustering for all but two plots, but was also true for MPD and PD for the tropical communities. This clustering of related species could be attributable to several factors. From the perspective of community ecology, these observations are consistent with local scale environmental filtering for phylogenetically conserved traits and niche conservatism. We note that with such geographically widespread communities other factors, including dispersal limitation linked with regional vicariance speciation, will play important roles and will require further investigation. Null models of no-dispersal limitation among communities will need to be explicitly re-examined in future work as we continue to construct phylogenies that encompass an increased number of communities.

With respect to environmental filtering and niche conservatism, these two processes are not mutually exclusive, although they make different assumptions regarding the role of phylogenetic conservatism and the role of dispersal. Much work has been done on the degree to which trait conservatism occurs in tropical forests (reviewed in Cavender-Bares et al., 2009) and the role of trait conservatism on phylogenetic pattern (Kraft et al., 2007; Crisp et al., 2009). Kraft et al. (2011) demonstrated that increasing phylogenetic trait conservation will amplify phylogenetic structure, which results in communities composed of more closely related sets of species. Crisp et al. (2009) examined phylogenetic distribution across major South American biomes and found a high degree of constraint on the ability of related groups to invade novel biomes. These results are concordant with our observations of the tropical communities studied here, in which species in each community tended to be phylogenetically clustered. A growing number of studies (e.g., Hardy et al., 2012; Ricklefs et al., 2012) have found evidence for globally-scaled processes regulating species diversity in the tropics. For example, in the neotropics the number of individuals and the number of species in certain families is strongly conserved across five replicated forest plots (Ricklefs et al., 2012). While the main objective of that particular study was an evaluation of the theory of ecological neutrality in community assembly (Hubbell, 2001), the results are concordant with high levels of phylogenetic trait conservatism and environmental filtering (Kraft et al., 2011). In some cases, field-based studies have shown mixed results in linking phylogenetic signal to trait dispersion in tropical forests (Liu et al., 2013). Therefore, even though the current results are consistent with a global pattern of environmental filtering and niche conservatism as a driving force in community assembly, more work needs to be done to clarify the role of phylogenetic trait conservatism in large-scale community processes.

### Conflict of interest statement

The authors declare that the research was conducted in the absence of any commercial or financial relationships that could be construed as a potential conflict of interest.
