# Supplementary material for: Comparative evolutionary diversity and phylogenetic structure across multiple forest dynamics plots: a mega-phylogeny approach
Source: Front Genet. 2014 Nov 5;5:358. doi: 10.3389/fgene.2014.00358 (PMC4220724; doi:10.3389/fgene.2014.00358)
Supplement: Supplementary file 1 [file Table1.DOCX]

Supplemental Table S1

| **Taxon** | **Author** | **Family** | **Site** | **rbcL** | **matK** | **psbA_trnH** |
| --- | --- | --- | --- | --- | --- | --- |
| Abarema macradenia | (Pittier)Barneby&J.W.Grimes | Fabaceae | BCI | GQ981652 | GQ981925 | GQ982133 |
| Abies amabilis | Douglas ex J. Forbes | Pinaceae | Wind_River | JN935604.1 | n/a | n/a |
| Abies balsamea | (L.) Mill. | Pinaceae | SERC | KJ593167 | n/a | n/a |
| Abies concolor | (Gordon & Glend.) Lindl. ex Hildebr. | Pinaceae | Yosemite | JN935650.1 | n/a | n/a |
| Abies holophylla | Maxim. | Pinaceae | Changbaishan | CANGB001-14 | n/a | n/a |
| Abies magnifica | A. Murray bis | Pinaceae | Yosemite | AB029649.1 | n/a | n/a |
| Abies nephrolepis | (Trautv. ex Maxim.) Maxim. | Pinaceae | Changbaishan | CANGB002-14 | n/a | n/a |
| Abies procera | Rehder | Pinaceae | Wind_River | AB029651.1 | n/a | n/a |
| Acalypha diversifolia | Jacq. | Euphorbiaceae | BCI | GQ981653 | n/a | GQ982134 |
| Acalypha macrostachya | Jacq. | Euphorbiaceae | BCI | GQ981654 | GQ981926 | GQ982135 |
| Acer barbinerve | Maxim. ex Miq. | Sapindaceae | Changbaishan | CANGB003-14 | n/a | n/a |
| Acer campestre | L. | Sapindaceae | Wytham | n/a | FJ395381.1 | FJ395476.1 |
| Acer ginnala | Maxim. | Sapindaceae | Changbaishan | CANGB004-14 | n/a | n/a |
| Acer mandshuricum | Maxim. | Sapindaceae | Changbaishan | CANGB005-14 | n/a | n/a |
| Acer mono | Maxim. | Sapindaceae | Changbaishan | CANGB006-14 | n/a | n/a |
| Acer negundo | L. | Sapindaceae | SERC | HM849738.1 | EU749281.1 | DQ006227.1 |
| Acer olivaceum | W.P. Fang & P.L. Chiu | Sapindaceae | Gutianshan | HQ427190.1 | HQ427338 | n/a |
| Acer platanoides | L. | Sapindaceae | SCBI | EU676888.1 | EU749282.1 | EU750429.1 |
| Acer pseudoplatanus | L. | Sapindaceae | Wytham | HE963306.1 | n/a | HE966461.1 |
| Acer pseudosieboldianum | (Pax) Kom. | Sapindaceae | Changbaishan | CANGB007-14 | n/a | n/a |
| Acer rubrum | L. | Sapindaceae | SERC | KJ593173 | KJ592831 | HQ266324.1 |
| Acer saccharum | Marshall | Sapindaceae | SERC | KJ593174 | EU749290.1 | EU750438.1 |
| Acer spicatum | Lam. | Sapindaceae | Wabikon Lake | KJ593177 | KJ592832 | n/a |
| Acer tegmentosum | Maxim. | Sapindaceae | Changbaishan | CANGB008-14 | n/a | n/a |
| Acer triflorum | Kom. | Sapindaceae | Changbaishan | CANGB009-14 | n/a | n/a |
| Acer ukurunduense | Trautv. & C.A.Mey. | Sapindaceae | Changbaishan | CANGB010-14 | n/a | n/a |
| Acer wilsonii | Rehder | Sapindaceae | Gutianshan | HQ427189.1 | HQ427337.1 | n/a |
| Acronychia pedunculata | (L.) Miq. | Rutaceae | Dinghushan | HQ415180 | HQ415351 | HQ415529 |
| Actinodaphne malaccensis | Hook. f. | Lauraceae | Bukit-Timah | KJ594563 | KJ708793 | BTSIN145-13 |
| Adelia triloba | (MŸll.Arg.)Hemsl. | Euphorbiaceae | BCI | GQ981655 | GQ981927 | GQ982136 |
| Adenanthera malayana | Kosterm. | Fabaceae | Bukit-Timah | KJ594564 | KJ708795 | BTSIN315-13 |
| Adenanthera microsperma | Teijsm. & Binn. | Fabaceae:Mimos. | Dinghushan | HQ415090.1 | HQ415273.1 | HQ415441.1 |
| Adinandra dumosa | Jack | Pentaphylacaceae | Bukit-Timah | n/a | KJ708799 | BTSIN588-13 |
| Adinandra integerrima | T. Anderson | Pentaphylacaceae | Bukit-Timah | n/a | KJ708801 | BTSIN599-13 |
| Adinandra milletii | Benth. & Hook. f. ex Hance | Pentaphylacaceae | Gutianshan | HQ427223.1 | HQ427369.1 | n/a |
| Aegiphila panamensis | Moldenke | Lamiaceae | BCI | GQ981656 | GQ981928 | GQ982137 |
| Aeschynomene americana | L. | Fabaceae | Luquillo | AF30870* | n/a | n/a |
| Aglaia elliptica | Blume | Meliaceae | Bukit-Timah | n/a | KJ708802 | BTSIN223-13 |
| Aglaia exstipulata | W. Theob. | Meliaceae | Bukit-Timah | KJ594567 | KJ708803 | BTSIN451-13 |
| Aglaia glabriflora | Hiern | Meliaceae | Bukit-Timah | KJ594568 | KJ708804 | n/a |
| Aglaia macrocarpa | (Miq.) Pannell | Meliaceae | Bukit-Timah | KJ594569 | KJ708806 | BTSIN353-13 |
| Aglaia malaccensis | (Ridl.) Pannell | Meliaceae | Bukit-Timah | KJ594570 | KJ708807 | BTSIN027-13 |
| Aglaia rufinervis | Bentv. | Meliaceae | Bukit-Timah | KJ594573 | KJ708809 | BTSIN292-13 |
| Aglaia sexipetala | Griff. | Meliaceae | Bukit-Timah | n/a | KJ708810 | BTSIN647-13 |
| Agrostistachys longifolia | Kurz | Euphorbiaceae | Bukit-Timah | BTSIN126-13 | BTSIN126-13 | BTSIN126-13 |
| Aidia canthioides | (Champ. ex Benth.) Masam. | Rubiaceae | Dinghushan | HQ415144 | n/a | HQ415494 |
| Aidia cochinchinensis | Lour. | Rubiaceae | Gutianshan | HQ427198.1 | HQ427347 | n/a |
| Aidia wallichiana | Tirveng. | Rubiaceae | Bukit-Timah | KJ594574 | KJ708811 | BTSIN392-13 |
| Alangium longiflorum | Merr. | Cornaceae | Bukit-Timah | KJ594578 | KJ708815 | BTSIN196-13 |
| Alangium nobile | (C.B. Clarke) Harms | Cornaceae | Bukit-Timah | KJ594579 | KJ708816 | BTSIN296-13 |
| Albizia julibrissin | Durazz. | Fabaceae | SERC | GU135262.1 | EU812010.1 | n/a |
| Albizia kalkora | (Roxb.) Prain | Fabaceae:Mimos. | Gutianshan | HQ427141.1 | HQ427295.1 | n/a |
| Albizia splendens | Miq. | Fabaceae | Bukit-Timah | n/a | KJ708817 | BTSIN198-13 |
| Alchornea costaricensis | Pax&K.Hoffm. | Euphorbiaceae | BCI | JQ591406.1 | n/a | n/a |
| Alchornea latifolia | Sw. | Euphorbiaceae | BCI/Luquillo | HM446755 | n/a | HM446884 |
| Alchornea trewioides | Benth. ex Müll. Arg. | Euphorbiaceae | Dinghushan | HQ415177 | HQ415348 | HQ415526 |
| Alchorneopsis floribunda | (Benth.) M¸ll. Arg. | Euphorbiaceae | Luquillo | HM446756 | HM446655 | HM446885 |
| Aleurites montana | (Lour.) E.H. Wilson | Euphorbiaceae | Lienhuachih | KJ687971 | NA | KJ686651 |
| Alfaropsis roxburghiana | (Wall.) Iljinsk. | Juglandaceae | Dinghushan | KJ440015.1 | n/a | KF201462.1 |
| Alibertia edulis | (Rich.)A.Rich.exDC. | Rubiaceae | BCI | GQ981657 | GQ981930 | GQ982139 |
| Allophylus psilospermus | Radlk. | Sapindaceae | BCI | GQ981658 | n/a | GQ982140 |
| Alniphyllum fortunei | (Hemsl.) Makino | Styracaceae | Gutianshan | HQ427122.1 | HQ427279.1 | n/a |
| Alniphyllum pterospermum | Matsum. | Styracaceae | Fushan | KJ687974 | NA | NA |
| Alnus incana | (L.) Moench | Betulaceae | Wabikon Lake | KJ593202 | KJ592842 | n/a |
| Alnus mandshurica | (Callier ex C.K. Schneid.) Hand.-Mazz. | Betulaceae | Changbaishan | CANGB011-14 | n/a | n/a |
| Alphonsea johorensis | J. Sinclair | Annonaceae | Bukit-Timah | n/a | KJ708818 | BTSIN323-13 |
| Alseis blackiana | Hemsl. | Rubiaceae | BCI | GQ981659 | n/a | GQ982141 |
| Alstonia angustifolia | Wall. | Apocynaceae | Bukit-Timah | KJ594580 | KJ708820 | BTSIN225-13 |
| Amaioua corymbosa | Kunth | Rubiaceae | BCI | GQ981660 | GQ981931 | GQ982142 |
| Amelanchier arborea | (F. Michx.) Fernald | Rosaceae | SERC | JQ391252.1 | JQ390935.1 | n/a |
| Amelanchier laevis | Wiegand | Rosaceae | Wabikon Lake | HQ589949.1 | n/a | n/a |
| Anacardium excelsum | (Kunth) Skeels | Anacardiaceae | BCI | GQ981661 | GQ981932 | GQ982143 |
| Anaxagorea panamensis | Standl. | Annonaceae | BCI | GQ981662 | GQ981933 | GQ982144 |
| Andira inermis | (W.Wright)KunthexDC. | Fabaceae:Papil. | BCI/Luquillo | HM446757 | n/a | HM446886 |
| Anisophyllea disticha | Baill. | Anisophylleaceae | Bukit-Timah | BTSIN375-13 | BTSIN375-13 | BTSIN375-13 |
| Anisophyllea griffithii | Oliv. | Anisophylleaceae | Bukit-Timah | BTSIN139-13 | BTSIN139-13 | BTSIN139-13 |
| Anisoptera costata | Korth. | Dipterocarpaceae | Bukit-Timah | KJ594581 | KJ708821 | BTSIN331-13 |
| Anisoptera laevis | Ridl. | Dipterocarpaceae | Bukit-Timah | KJ594582 | n/a | BTSIN070-13 |
| Anisoptera megistocarpa | Slooten | Dipterocarpaceae | Bukit-Timah | KJ594584 | KJ708822 | BTSIN034-13 |
| Annona acuminata | Saff. | Annonaceae | BCI | GQ981664 | GQ981934 | GQ982146 |
| Annona purpurea | Moc.&SessŽexDunal | Annonaceae | BCI | JQ590160.1 | JQ586490.1 | n/a |
| Annona spraguei | Saff. | Annonaceae | BCI | GQ981665 | GQ981935 | GQ982147 |
| Antiaris toxicaria | Lesch. | Moraceae | Bukit-Timah | KJ594588 | n/a | BTSIN053-13 |
| Antidesma bunius | (L.) Spreng. | Phyllanthaceae | Dinghushan | HQ415206 | n/a | HQ415553 |
| Antidesma coriaceum | Tul. | Phyllanthaceae | Bukit-Timah | KJ594590 | n/a | BTSIN089-13 |
| Antidesma fordii | Hemsl. | Phyllanthaceae | Dinghushan | HQ415204 | HQ415370 | HQ415551 |
| Antidesma japonicum var acutisepalum | n/a | Phyllanthaceae | Fushan/Lienhuachih | AB925514.1 | NA | KJ686654 |
| Antidesma japonicum var. densiflorum | (Hayata) Hurus. | Phyllanthaceae | Fushan | KJ687977 | NA | KJ686655 |
| Antidesma neurocarpum | Miq. | Phyllanthaceae | Bukit-Timah | KJ594591 | KJ708824 | n/a |
| Antidesma venosum | E. Mey. ex Tul. | Phyllanthaceae | Dinghushan | HQ415207 | HQ415372 | HQ415554 |
| Antirhea obtusifolia | Urb. | Rubiaceae | Luquillo | HM446758 | HM446656 | HM446887 |
| Apeiba membranacea | SpruceexBenth. | Malvaceae | BCI | GQ981666 | n/a | n/a |
| Apeiba tibourbou | Aubl. | Malvaceae | BCI | GQ981667 | GQ981936 | GQ982148 |
| Aphelandra sinclairiana | Nees | Acanthaceae | BCI | GQ981668 | GQ981937 | GQ982149 |
| Aporusa benthamiana | Hook. f. | Phyllanthaceae | Bukit-Timah | KJ594594 | KJ708826 | BTSIN200-13 |
| Aporusa falcifera | Hook. f. | Phyllanthaceae | Bukit-Timah | KJ594596 | n/a | n/a |
| Aporusa frutescens | Benth. | Phyllanthaceae | Bukit-Timah | KJ594599 | KJ708827 | BTSIN457-13 |
| Aporusa lunatum | n/a | Phyllanthaceae | Bukit-Timah | KJ594600 | KJ708829 | n/a |
| Aporusa microstachya | (Tul.) MŸll.Arg. | Phyllanthaceae | Bukit-Timah | KJ594601 | KJ708830 | BTSIN086-13 |
| Aporusa miqueliana | MŸll. Arg. | Phyllanthaceae | Bukit-Timah | KJ594603 | KJ708831 | BTSIN469-13 |
| Aporusa nervosa | Hook. f. | Phyllanthaceae | Bukit-Timah | KJ594605 | n/a | BTSIN235-13 |
| Aporusa yunnanensis | (Pax & K. Hoffm.) F.P. Metcalf | Phyllanthaceae | Dinghushan | HQ415224.1 | HQ415388.1 | HQ415570.1 |
| Aquilaria sinensis | (Lour.) Spreng. | Thymelaeaceae | Dinghushan | HQ415056 | HQ415244 | HQ415408 |
| Aralia chinensis | L. | Araliaceae | Gutianshan | HQ427250.1 | HQ427393 | n/a |
| Aralia decaisneana | Hance | Araliaceae | Fushan | KJ687982 | KJ687454 | KJ686659 |
| Aralia spinifolia | Merr. | Araliaceae | Dinghushan | HQ415059 | HQ415247 | HQ415411 |
| Archidendron clypearia | (Jack) I.C. Nielsen | Fabaceae | Bukit-Timah | n/a | KJ708833 | n/a |
| Archidendron lucidum | (Benth.) I.C. Nielsen | Fabaceae-Mimosoideae | Dinghushan/Fushan | HQ415101 | HQ415282 | HQ415452 |
| Archidendron turgidum | (Merr.) I.C. Nielsen | Fabaceae | Dinghushan | HQ415094.1 | n/a | HQ415445.1 |
| Ardisia bartlettii | Lundell | Primulaceae | BCI | GQ981669 | GQ981938 | GQ982150 |
| Ardisia colorata | Roxb. | Primulaceae | Bukit-Timah | KJ594606 | KJ708834 | BTSIN262-13 |
| Ardisia cornudentata subsp. morrisonensis | subsp. morrisonensis (Hayata) Yuen P. Yang | Primulaceae | Fushan | KJ687988 | NA | NA |
| Ardisia crenata | Sims | Primulaceae | Gutianshan/Fushan | HQ427270.1 | HQ427412 | n/a |
| Ardisia glauca | Mez | Primulaceae | Luquillo | KJ082123 | KJ012464 | n/a |
| Ardisia guianensis | (Aubl.)Mez | Primulaceae | BCI | GQ981670 | GQ981939 | GQ982151 |
| Ardisia quinquegona | Blume | Primulaceae | Dinghushan/Fushan | HQ415236 | HQ415400 | HQ415582 |
| Ardisia sieboldii | Kurz | Primulaceae | Fushan | NA | NA | KJ686669 |
| Ardisia standleyana | P.H.Allen | Primulaceae | BCI | GQ981671 | GQ981940 | GQ982152 |
| Ardisia teysmanniana | Scheff. | Primulaceae | Bukit-Timah | KJ594608 | n/a | BTSIN368-13 |
| Ardisia virens | Sims | Primulaceae | Fushan | KJ687998 | KJ687460 | KJ686673 |
| Arthrophyllum diversifolium | (Däniker) Philipson | Araliaceae | Bukit-Timah | KJ594609 | KJ708836 | n/a |
| Artocarpus altilis | (Parkinson)Fosberg | Moraceae | Luquillo | HM446760 | HM446658 | HM446889 |
| Artocarpus anisophyllus | Miq. | Moraceae | Bukit-Timah | KJ594612 | n/a | BTSIN155-13 |
| Artocarpus dadah | Miq. | Moraceae | Bukit-Timah | KJ594613 | n/a | BTSIN170-13 |
| Artocarpus elasticus | Reinw. ex Blume | Moraceae | Bukit-Timah | KJ594614 | KJ708837 | BTSIN424-13 |
| Artocarpus hispidus | F.M.Jarrett | Moraceae | Bukit-Timah | n/a | KJ708839 | BTSIN440-13 |
| Artocarpus integer | Merr. | Moraceae | Bukit-Timah | KJ594617 | KJ708840 | BTSIN466-13 |
| Artocarpus kemando | Miq. | Moraceae | Bukit-Timah | KJ594618 | KJ708841 | n/a |
| Artocarpus lanceifolius | Roxb. | Moraceae | Bukit-Timah | KJ594619 | KJ708842 | BTSIN017-13 |
| Artocarpus lowii | King | Moraceae | Bukit-Timah | KJ594620 | KJ708843 | n/a |
| Artocarpus rigidus | Blume | Moraceae | Bukit-Timah | KJ594621 | KJ708844 | BTSIN348-13 |
| Artocarpus scortechinii | King | Moraceae | Bukit-Timah | KJ594622 | KJ708845 | BTSIN212-13 |
| Artocarpus styracifolius | Pierre | Moraceae | Dinghushan | HQ415055 | HQ415243 | HQ415407 |
| Asimina triloba | (L.) Dunal | Annonaceae | SERC | L12631.2 | n/a | n/a |
| Aspidosperma spruceanum | Benth.exMŸll.Arg. | Apocynaceae | BCI | GQ981672 | GQ981941 | GQ982153 |
| Astrocaryum standleyanum | L.H.Bailey | Arecaceae | BCI | GQ981673 | GQ981942 | GQ982154 |
| Astronium graveolens | Jacq. | Anacardiaceae | BCI | GQ981674 | n/a | GQ982155 |
| Attalea rostrata | Oerst. | Arecaceae | BCI | GQ981675 | GQ981943 | GQ982156 |
| Aucuba chinensis | Benth. | Garryaceae | Lienhuachih | KJ688006 | KJ687468 | KJ686681 |
| Baccaurea macrocarpa | (Miq.) Müll. Arg. | Phyllanthaceae | Bukit-Timah | KJ594624 | KJ708847 | BTSIN437-13 |
| Bactris barronis | L.H.Bailey | Arecaceae | BCI | GQ981676 | GQ981944 | GQ982157 |
| Bactris coloniata | L.H.Bailey | Arecaceae | BCI | GQ981677 | n/a | GQ982158 |
| Bactris major | Jacq. | Arecaceae | BCI | GQ981678 | GQ981945 | GQ982159 |
| Beilschmiedia erythrophloia | Hayata | Lauraceae | Lienhuachih | KJ688008 | NA | KJ686684 |
| Beilschmiedia kunstleri | Gamble | Lauraceae | Bukit-Timah | n/a | KJ708848 | n/a |
| Beilschmiedia pendula | (Sw.)Hemsl. | Lauraceae | Luquillo | GQ981679* | n/a | n/a |
| Beilschmiedia tovarensis | (Klotzsch & H. Karst. ex Meisn.) Sach. Nishida | Lauraceae | BCI | GQ981679 | n/a | GQ982160 |
| Betula alleghaniensis | Britton | Betulaceae | Wabikon Lake | KJ593243 | KJ592868 | n/a |
| Betula costata | Trautv. | Betulaceae | Changbaishan | CANGB012-14 | n/a | n/a |
| Betula ermanii | Cham. | Betulaceae | Changbaishan | CANGB013-14 | n/a | n/a |
| Betula papyrifera | Marshall | Betulaceae | Wabikon Lake | KJ593244 | KJ592869 | n/a |
| Betula platyphylla | Sukaczev | Betulaceae | Changbaishan | CANGB014-14 | n/a | n/a |
| Bhesa robusta | (Roxb.) Ding Hou | Celastraceae | Bukit-Timah | KJ594626 | KJ708849 | n/a |
| Bischofia javanica | Blume | Phyllanthaceae | Lienhuachih | AY663571.1 | AY552420.1 | n/a |
| Blastus cochinchinensis | Lour. | Lamiaceae | Dinghushan/Fushan | KJ688010 | NA | KJ686685 |
| Borojoa panamensis | Dwyer | Rubiaceae | BCI | GQ981680 | GQ981946 | GQ982161 |
| Brackenridgea hookeri | A. Gray | Ochnaceae | Bukit-Timah | KJ594629 | n/a | BTSIN432-13 |
| Bridelia retusa | (L.) A. Juss. | Phyllanthaceae | Dinghushan | n/a | FJ439945.1 | n/a |
| Bridelia tomentosa | Blume | Phyllanthaceae | Lienhuachih | KJ688016 | NA | NA |
| Brosimum alicastrum | Sw. | Moraceae | BCI | GQ981681 | GQ981947 | GQ982162 |
| Brosimum guianense | (Aubl.)Huber | Moraceae | BCI | GQ981682 | GQ981948 | GQ982163 |
| Brunfelsia portoricensis | Krug & Urb. | Solanaceae | Luquillo | HM446761 | HM446659 | HM446890 |
| Buchanania sessifolia | Blume | Anacardiaceae | Bukit-Timah | KJ594630 | KJ708850 | BTSIN312-13 |
| Buchenavia tetraphylla | (Aubl.)R.A.Howard | Combretaceae | Luquillo | HM446762 | HM446660 | HM446891 |
| Bunchosia nitida | (Jacq.)DC. | Malpighiaceae | BCI | n/a | KJ012484 | KJ426629 |
| Byrsonima spicata | (Cav.)DC. | Malpighiaceae | Luquillo | HM446763 | HM446661 | HM446892 |
| Byrsonima wadsworthii | Little | Malpighiaceae | Luquillo | HM446764 | HM446662 | HM446893 |
| Callicarpa bodinieri | H.Lév. | Verbenaceae | Gutianshan | HQ427182.1 | HQ427330 | n/a |
| Callicarpa giraldii | Hesse ex Rehder | Verbenaceae | Gutianshan | HQ427184.1 | HQ427332 | n/a |
| Callicarpa kochiana | Makino | Verbenaceae | Fushan | KJ688022 | KJ687476 | KJ686695 |
| Callicarpa rubella | Lindl. | Verbenaceae | Gutianshan | HQ427181.1 | HQ427329 | n/a |
| Callicarpa tikusikensis | Masam. | Verbenaceae | Fushan | KJ688027 | KJ687481 | KJ686700 |
| Calocedrus decurrens | (Torr.) Florin | Cupressaceae | Yosemite | NC_023121 | NC_023121 | NC_023121 |
| Calophyllum calaba | L. | Clusiaceae | Luquillo | HM446765 | n/a | HM446894 |
| Calophyllum incrassatum | M.R. Hend. & Wyatt-Sm. | Clusiaceae | Bukit-Timah | KJ594633 | n/a | BTSIN456-13 |
| Calophyllum longifolium | Willd. | Clusiaceae | BCI | GQ981683 | n/a | GQ982164 |
| Calophyllum macrocarpum | Hook. f. | Clusiaceae | Bukit-Timah | KJ594637 | n/a | n/a |
| Calophyllum membranaceum | Gardner & Champ. | Clusiaceae | Dinghushan | HQ415099 | n/a | HQ415450 |
| Calophyllum pulcherrimum | Wall. ex Choisy | Clusiaceae | Bukit-Timah | KJ594635 | n/a | n/a |
| Calophyllum teysmannii | Miq. | Clusiaceae | Bukit-Timah | KJ594636 | n/a | BTSIN197-13 |
| Calophyllum wallichianum | Planch. & Triana | Clusiaceae | Bukit-Timah | KJ594637 | n/a | n/a |
| Calycogonium squamulosum | Cogn. | Melastomataceae | Luquillo | HM446766 | n/a | HM446895 |
| Camellia chekiang oleosa | Hu | Theaceae | Gutianshan | HQ427229.1 | n/a | n/a |
| Camellia cuspidata | Bean | Theaceae | Gutianshan | HQ427225.1 | HQ427370 | n/a |
| Camellia fraterna | Hance | Theaceae | Gutianshan | HQ427224.1 | n/a | n/a |
| Camellia furfuracea | (Merr.) Cohen-Stuart | Theaceae | Lienhuachih | KJ688031 | KJ687485 | KJ686704 |
| Camellia nokoensis | Hayata | Theaceae | Lienhuachih | KJ688034 | KJ687486 | KJ686705 |
| Camellia salicifolia | Champ. ex Benth. | Theaceae | Lienhuachih | KJ688037 | NA | KJ686707 |
| Campnosperma auriculatum | (Blume) Hook. f. | Anacardiaceae | Bukit-Timah | KJ594639 | KJ708854 | BTSIN249-13 |
| Canarium album | Leenh. | Burseraceae | Dinghushan | HQ415083 | HQ415266 | HQ415434 |
| Canarium littorale | Blume | Burseraceae | Bukit-Timah | n/a | KJ708855 | BTSIN606-13 |
| Canarium pilosum | A.W. Benn. | Burseraceae | Bukit-Timah | KJ594640 | KJ708856 | n/a |
| Canarium tramdenum | C.D. Dai & Yakovlev | Burseraceae | Dinghushan | HQ415084 | HQ415267 | HQ415435 |
| Canthium confertum | Korth. | Rubiaceae | Bukit-Timah | n/a | n/a | BTSIN144-13 |
| Canthium glabrum | Blume | Rubiaceae | Bukit-Timah | KJ594641 | n/a | BTSIN035-13 |
| Canthium horridum | Blume | Rubiaceae | Dinghushan | HQ415226.1 | HQ415390.1 | HQ415572.1 |
| Capparis frondosa | Jacq. | Capparaceae | BCI | GQ981684 | GQ981949 | GQ982165 |
| Capparis sabiaefolia | Hook.f. & Thomson | Capparaceae | Lienhuachih | KJ688041 | KJ687488 | KJ686634 |
| Carallia brachiata | (Lour.) Merr. | Rhizophoraceae | Dinghushan | HQ415233 | HQ415397 | HQ415579 |
| Carpinus betulus | L. | Betulaceae | Wytham | JN893226.1 | JN895396.1 | n/a |
| Carpinus caroliniana | Walter | Betulaceae | SERC | KJ593289 | KJ592883 | n/a |
| Carpinus viminea | Lindl. | Betulaceae | Gutianshan | HQ427161.1 | n/a | n/a |
| Carya cordiformis | (Wangenh.) K. Koch | Juglandaceae | SERC | KJ593294 | KJ592887 | HQ596633.1 |
| Carya glabra | (Mill.) Sweet | Juglandaceae | SERC | L12637.2 | KF201333.1 | KF201453.1 |
| Carya laciniosa | (F. Michx.) Loudon | Juglandaceae | SCBI | L12637.2 | n/a | KF201451.1 |
| Carya ovalis | (Wangenh.) Sarg. | Juglandaceae | SCBI | L12637.2 | n/a | KF201453.1 |
| Carya ovata | (Mill.) K. Koch | Juglandaceae | SERC | AY263931.1 | U92850.1 | KF201454.1 |
| Carya tomentosa | (Poir.) Nutt. | Juglandaceae | SERC | n/a | AF118039.1 | KF201458.1 |
| Caryota maxima | Blume ex Mart. | Arecaceae | Dinghushan | HQ415227 | HQ415391 | HQ415573 |
| Caryota mitis | Lour. | Arecaceae | Bukit-Timah | KJ594645 | KJ708860 | n/a |
| Casearia aculeata | Jacq. | Salicaceae | BCI | GQ981685 | GQ981950 | GQ982166 |
| Casearia arborea | (Rich.)Urb. | Flacourtiaceae | Luquillo | GQ981686 | HM446663 | HM446896 |
| Casearia commersoniana | Cambess. | Salicaceae | BCI | GQ981687 | GQ981952 | GQ982168 |
| Casearia glomerata | Roxb. | Salicaceae | Dinghushan | HQ415115 | HQ415293 | HQ415465 |
| Casearia guianensis | (Aubl.)Urb. | Salicaceae | BCI | HM446767 | n/a | HM446897 |
| Casearia membranacea | Hance | Flacourtiaceae | Fushan | KJ688042 | NA | KJ686709 |
| Casearia sylvestris | Sw. | Flacourtiaceae | Luquillo | HM446768 | HM446664 | HM446898 |
| Casearia velutina | Blume | Salicaceae | Dinghushan | HQ415116.1 | HQ415294.1 | HQ415466.1 |
| Cassipourea elliptica | (Sw.)Poir. | Rhizophoraceae | BCI | GQ981690 | GQ981955 | GQ982171 |
| Cassipourea guianensis | Aubl. | Rhizophoraceae | Luquillo | HM446769 | HM446665 | HM446899 |
| Castanea dentata | (Marshall) Borkh. | Fagaceae | SCBI | KF613012.1 | n/a | JQ677929.1 |
| Castanea mollissima | Blume | Fagaceae | SERC | HQ336406.1 | U92862.1 | n/a |
| Castanea sativa | Mill. | Fagaceae | Wytham | M94936.1 | n/a | FN687510.1 |
| Castanopsis carlesii | (Hemsl.) Hayata | Fagaceae | Gutianshan | HQ427175.1 | HQ427323 | n/a |
| Castanopsis chinensis | (Abel) Schottky | Fagaceae | Dinghushan | HQ415235 | HQ415399 | HQ415581 |
| Castanopsis cuspidata var. carlesii | (Hemsl.)T.Yamaz. | Fagaceae | Fushan | KJ688046 | KJ687491 | KJ686711 |
| Castanopsis eyrei | (Champ. ex Benth.) Tutcher | Fagaceae | Gutianshan | HQ427167.1 | HQ427315 | n/a |
| Castanopsis fargesii | Franch. | Fagaceae | Gutianshan/Lienhuachih | KJ688056 | KJ687501 | KJ686721 |
| Castanopsis fissa | (Champ. ex Benth.) Rehder & E.H. Wilson | Fagaceae | Dinghushan | HQ415234 | HQ415398 | HQ415580 |
| Castanopsis kawakamii | Hayata | Fagaceae | Lienhuachih | KJ688057 | KJ687503 | KJ686723 |
| Castanopsis lucida | (Nees) Soepadmo | Fagaceae | Bukit-Timah | KJ594649 | KJ708864 | BTSIN101-13 |
| Castanopsis tibetana | Hance | Fagaceae | Gutianshan | HQ427172.1 | HQ427320 | n/a |
| Catunaregam spinosa | (Thunb.) Tirveng. | Rubiaceae | Dinghushan | HQ415178 | HQ415349 | HQ415527 |
| Cavanillesia platanifolia | (Bonpl.)Kunth | Malvaceae | BCI | GQ981691 | GQ981956 | GQ982172 |
| Cecropia insignis | Liebm. | Urticaceae | BCI | GQ981692 | n/a | GQ982173 |
| Cecropia longipes | Pittier | Urticaceae | BCI | GQ981693 | GQ981957 | GQ982174 |
| Cecropia obtusifolia | Bertol. | Urticaceae | BCI | GQ981694 | GQ981958 | GQ982175 |
| Cecropia schreberiana | Miq. | Urticaceae | Luquillo | HM446770 | HM446666 | HM446900 |
| Cedrela odorata | L. | Meliaceae | BCI | GQ981695 | GQ981959 | GQ982176 |
| Ceiba pentandra | (L.)Gaertn. | Bombacaceae | Luquillo | GQ981696 | GQ981960 | GQ982177 |
| Celtis biondii | Pamp. | Ulmaceae | Gutianshan | HQ427254.1 | n/a | n/a |
| Celtis formosana | Hayata | Cannabaceae | Lienhuachih | KJ688060 | NA | KJ686725 |
| Celtis occidentalis | L. | Cannabaceae | SERC | JX571800.1 | n/a | n/a |
| Celtis schippii | Standl. | Cannabaceae | BCI | GQ981697 | GQ981961 | GQ982178 |
| Cercis canadensis | L. | Fabaceae | SCBI | NC_024057.1 | NC_024057.1 | NC_024057.1 |
| Cespedesia spathulata | (Ruiz&Pav.)Planch. | Ochnaceae | BCI | GQ981698 | n/a | GQ982179 |
| Cestrum macrophyllum | Vent. | Solanaceae | Luquillo | HM446771 | HM446667 | HM446901 |
| Cestrum megalophyllum | Dunal | Solanaceae | BCI | JQ594113.1 | n/a | GQ982180 |
| Chamaedorea tepejilote | Liebm. | Arecaceae | BCI | GQ981699 | GQ981962 | GQ982181 |
| Chamguava schippii | (Standl.)Landrum | Myrtaceae | BCI | n/a | GQ981963 | GQ982182 |
| Cheilosa montana | Blume | Euphorbiaceae | Bukit-Timah | KJ594650 | KJ708865 | BTSIN003-13 |
| Chimarrhis parviflora | Standl. | Rubiaceae | BCI | GQ981700 | GQ981964 | GQ982183 |
| Chimonanthus salicifolius | S.Y. Hu | Calycanthaceae | Gutianshan | HQ427177.1 | HQ427325 | n/a |
| Chionanthus domingensis | Lam. | Oleaceae | Luquillo | HM446772 | n/a | HM446902 |
| Chionanthus virginicus | L. | Oleaceae | SCBI | DQ006108.1 | n/a | DQ006204.1 |
| Chione venosa | (Sw.)Urb. | Rubiaceae | Luquillo | KJ082185 | KJ012508 | KJ426653 |
| Chisocheton patens | Blume | Meliaceae | Bukit-Timah | KJ594651 | KJ708866 | BTSIN043-13 |
| Chisocheton sarawakanus | (C. DC.) Harms | Meliaceae | Bukit-Timah | KJ594652 | KJ708867 | BTSIN650-13 |
| Chrysochlamys eclipes | L.O.Williams | Clusiaceae | BCI | GQ981702 | n/a | GQ982184 |
| Chrysophyllum argenteum | Jacq. | Sapotaceae | Luquillo | HM446773 | HM446668 | HM446904 |
| Chrysophyllum cainito | L. | Sapotaceae | BCI | GQ981701 | GQ981966 | GQ982186 |
| Chrysophyllum lanceolatum | (Blume) A. DC. | Sapotaceae | Dinghushan | HQ415117 | HQ415295 | HQ415467 |
| Cinnamomum austrosinense | Hung T. Chang | Lauraceae | Fushan | KJ688061 | NA | KJ686727 |
| Cinnamomum camphora | (L.) J. Presl | Lauraceae | Lienhuachih | KJ688064 | NA | KJ686728 |
| Cinnamomum chekiangense | Nakai | Lauraceae | Gutianshan | HQ427267 | HQ427409 | n/a |
| Cinnamomum elongatum | (Nees) Kosterm. | Lauraceae | Luquillo | HM446774 | HM446669 | HM446905 |
| Cinnamomum micranthum | (Hayata) Hayata | Lauraceae | Fushan | KJ688068 | KJ687507 | KJ686732 |
| Cinnamomum montanum | (Sw.) Bercht. & J. Presl | Lauraceae | Luquillo | HM446775 | HM446670 | HM446906 |
| Cinnamomum osmophloeum | Kaneh. | Lauraceae | Lienhuachih | KJ688073 | KJ687512 | KJ686737 |
| Cinnamomum sintoc | Blume | Lauraceae | Bukit-Timah | KJ594654 | n/a | BTSIN326-13 |
| Cinnamomum subavenium | Miq. | Lauraceae | Gutianshan | HQ427266 | HQ427408 | n/a |
| Cinnamomum triplinerve | (Ruiz&Pav.)Kosterm. | Lauraceae | BCI | GQ981703 | GQ981967 | GQ982187 |
| Citharexylum caudatum | L. | Verbenaceae | Luquillo | HM446776 | HM446671 | HM446907 |
| Citharexylum fruticosum | L. | Verbenaceae | Luquillo | HM446777 | HM446672 | HM446908 |
| Citrus paradisi | Macfad. | Rutaceae | Luquillo | HM446778 | n/a | HM446909 |
| Clerodendron trichotomum | Thunb. | Lamiaceae | Gutianshan | HQ427186.1 | n/a | n/a |
| Clerodendrum cyrtophyllum | Turcz. | Lamiaceae | Lienhuachih | KJ688085 | KJ687521 | KJ686745 |
| Clerodendrum fortunatum | L. | Lamiaceae | Dinghushan | HQ415230 | HQ415394 | HQ415576 |
| Clerodendrum japonicum | (Thunb.) R. Sweet | Lamiaceae | Dinghushan | HQ415231 | HQ415395 | HQ415577 |
| Clerodendrum laevifolium | Blume | Lamiaceae | Bukit-Timah | KJ594655 | KJ708870 | BTSIN082-13 |
| Clerodendrum trichotomum | Thunb. | Lamiaceae | Lienhuachih | KJ688086 | KJ687522 | KJ686746 |
| Cleyera japonica | Thunb. | Pentaphylacaceae | Fushan | KJ688091 | KJ687527 | KJ686751 |
| Clibadium erosum | (Sw.) DC. | Asteraceae | Luquillo | HM446779 | HM446673 | HM446910 |
| Clidemia dentata | Pav.exD.Don | Melastomataceae | BCI | GQ981704 | n/a | GQ982188 |
| Clidemia octona | (Bonpl.)L.O.Williams | Melastomataceae | BCI | GQ981705 | n/a | GQ982189 |
| Clidemia septuplinervia | Cogn. | Melastomataceae | BCI | GQ981706 | GQ981968 | GQ982190 |
| Clusia rosea | Jacq. | Clusiaceae | Luquillo | HM446780 | n/a | HM446911 |
| Coccoloba coronata | Jacq. | Polygonaceae | BCI | GQ981707 | GQ981969 | GQ982191 |
| Coccoloba diversifolia | Jacq. | Polygonaceae | Luquillo | HM446781 | HM446674 | HM446912 |
| Coccoloba manzinellensis | Beurl. | Polygonaceae | BCI | GQ981708 | GQ981970 | GQ982192 |
| Coccoloba pyrifolia | Desf. | Polygonaceae | Luquillo | n/a | HM446675 | HM446913 |
| Coffea arabica | L. | Rubiaceae | Luquillo | HM446782 | HM446676 | HM446914 |
| Cojoba rufescens | (Benth.)Britton&Rose | Fabaceae | BCI | GQ981709 | GQ981971 | GQ982193 |
| Colubrina glandulosa | Perkins | Rhamnaceae | BCI | n/a | n/a | n/a |
| Comocladia glabra | Spreng. | Anacardiaceae | Luquillo | HM446783 | HM446677 | HM446915 |
| Conostegia bracteata | Triana | Melastomataceae | BCI | GQ981710 | n/a | GQ982194 |
| Conostegia cinnamomea | (Beurl.)Wurdack | Melastomataceae | BCI | GQ981711 | n/a | GQ982195 |
| Cordia alliodora | (Ruiz&Pav.)Oken | Boraginaceae | BCI | GQ981712 | GQ981972 | GQ982196 |
| Cordia bicolor | A.DC. | Boraginaceae | BCI | GQ981713 | GQ981973 | GQ982197 |
| Cordia borinquensis | Urb. | Boraginaceae | Luquillo | HM446784 | HM446678 | HM446916 |
| Cordia lasiocalyx | Pittier | Boraginaceae | BCI | GQ981714 | n/a | GQ982198 |
| Cordia sulcata | DC. | Boraginaceae | Luquillo | n/a | HM446679 | HM446917 |
| Cornus alternifolia | L. f. | Cornaceae | Wabikon Lake | KJ593329 | KJ592907 | n/a |
| Cornus florida | L. | Cornaceae | SERC | EU002276.1 | n/a | n/a |
| Cornus nuttallii | Audubon ex Torr. & A. Gray | Cornaceae | Wind_River | AF006833.1 | U96897.1 | n/a |
| Corylopsis glandulifera | Hemsl. | Hamamelidaceae | Gutianshan | HQ427165.1 | n/a | n/a |
| Corylus avellana | L. | Betulaceae | Wytham | AY263929.1 | AY263916.1 | n/a |
| Corylus cornuta | Marshall | Betulaceae | Wabikon Lake | KJ593340 | KJ592915 | n/a |
| Corylus mandshurica | Maxim. | Betulaceae | Changbaishan | CANGB015-14 | n/a | n/a |
| Coussarea curvigemmia | Dwyer | Rubiaceae | BCI | GQ981715 | GQ981974 | GQ982199 |
| Coutarea hexandra | (Jacq.)K.Schum. | Rubiaceae | BCI | GQ981716 | GQ981975 | GQ982200 |
| Craibiodendron scleranthum | (S.Y.Hu) Judd | Ericaceae | Dinghushan | HQ415118.1 | n/a | HQ415468.1 |
| Crataegus maximowiczii | C.K. Schneid. | Rosaceae | Changbaishan | CANGB016-14 | n/a | n/a |
| Crataegus monogyna | Jacq. | Rosaceae | Wytham | KC251347.1 | KC251105.1 | HG764982.1 |
| Cratoxylum arborescens | Blume | Clusiaceae | Bukit-Timah | KJ594656 | n/a | BTSIN454-13 |
| Cratoxylum cochinchinense | (Lour.) Blume | Clusiaceae | Dinghushan | HQ415110.1 | n/a | HQ415460.1 |
| Cratoxylum formosum | (Jack) Dyer | Clusiaceae | Bukit-Timah | KJ594660 | n/a | BTSIN131-13 |
| Croton billbergianus | MŸll.Arg. | Euphorbiaceae | BCI | GQ981717 | n/a | GQ982201 |
| Croton lachnocarpus | Benth. | Euphorbiaceae | Dinghushan | HQ415051 | HQ415239 | HQ415403 |
| Croton laevifolius | Blume | Euphorbiaceae | Bukit-Timah | KJ594661 | KJ708871 | BTSIN276-13 |
| Croton poecilanthus | Urb. | Euphorbiaceae | Luquillo | HM446785 | HM446680 | n/a |
| Crypteronia griffithii | C.B. Clarke | Crypteroniaceae | Bukit-Timah | KJ594662 | KJ708872 | BTSIN447-13 |
| Cryptocarya chinensis | (Hance) Hemsl. | Lauraceae | Fushan | KJ688094 | KJ687529 | KJ686754 |
| Cryptocarya concinna | Hance | Lauraceae | Nanjenshan | KJ688716 | KJ687907 | KJ687311 |
| Cryptocarya ferrea | Blume | Lauraceae | Bukit-Timah | KJ594663 | n/a | n/a |
| Cryptocarya nitens | Koord. & Valet | Lauraceae | Bukit-Timah | n/a | KJ708873 | n/a |
| Cryptocarya rugulosa | Hook. f. | Lauraceae | Bukit-Timah | KJ594663 | n/a | BTSIN122-13 |
| Cunninghamia lanceolata | (Lamb.) Hook. | Cupressaceae | Gutianshan | HQ427238 | HQ427381 | n/a |
| Cupania cinerea | Poepp. | Sapindaceae | BCI | GQ981718 | GQ981976 | GQ982202 |
| Cupania latifolia | Kunth | Sapindaceae | BCI | GQ981719 | GQ981977 | GQ982203 |
| Cupania rufescens | Triana&Planch. | Sapindaceae | BCI | GQ981720 | GQ981978 | GQ982204 |
| Cupania seemannii | Triana&Planch. | Sapindaceae | BCI | GQ981721 | GQ981979 | GQ982205 |
| Cyathea arborea | (L.) Sm. | Cyatheaceae | Luquillo | HM446786 | n/a | HM446918 |
| Cyathea borinquena | (Maxon) Domin | Cyatheaceae | Luquillo | HM446787 | n/a | HM446919 |
| Cyathea lepifera | (J. Sm. ex Hook.) Copel. | Cyatheaceae | Fushan | KJ688102 | NA | KJ686762 |
| Cyathea petiolata | (Hook.)R.M.Tryon | Cyatheaceae | BCI | EF463164.1 | n/a | n/a |
| Cyathea podophylla | (Hook.)Copel. | Cyatheaceae | Fushan | KJ688103 | NA | KJ686763 |
| Cyathea spinulosa | Wall. Ex Hook. | Cyatheaceae | Fushan | KJ688108 | NA | KJ686769 |
| Cyathocalyx ramuliflorus | (Maingay ex Hook. f. & Thomson) Scheff. | Annonaceae | Bukit-Timah | KJ594665 | KJ708874 | BTSIN246-13 |
| Cyclobalanopsis gilva | (Blume) Oerst. | Fagaceae | Fushan | KJ688109 | KJ687534 | KJ686770 |
| Cyclobalanopsis glauca | (Thunb.) Oerst. | Fagaceae | Gutianshan | AB060571.1 | HQ427316.1 | n/a |
| Cyclobalanopsis gracilis | (Rehder & E.H. Wilson) W.C. Cheng & T. Hong | Fagaceae | Gutianshan | HQ427176.1 | n/a | n/a |
| Cyclobalanopsis longinux | (Hayata) Schottky | Fagaceae | Fushan | KJ688116 | KJ687541 | KJ686777 |
| Cyclobalanopsis myrsinifolia | (Blume) Oerst. | Fagaceae | Gutianshan | HQ427170.1 | n/a | n/a |
| Cyclobalanopsis pachyloma | (Seemen) Schottky | Fagaceae | Lienhuachih | KJ688117 | KJ687542 | KJ686778 |
| Cyclobalanopsis sessilifolia | (Blume) Schottky | Fagaceae | Fushan | KJ688121 | NA | NA |
| Cyrilla racemiflora | L. | Cyrillaceae | Luquillo | HM446788 | HM446681 | HM446920 |
| Dacryodes costata | H.J. Lam | Burseraceae | Bukit-Timah | n/a | KJ708876 | BTSIN618-13 |
| Dacryodes excelsa | Vahl | Burseraceae | Luquillo | HM446789 | n/a | HM446921 |
| Dacryodes laxa | (A.W. Benn.) H.J. Lam | Burseraceae | Bukit-Timah | KJ594667 | n/a | BTSIN080-13 |
| Dacryodes nervosa | Leenh. | Burseraceae | Bukit-Timah | KJ594668 | KJ708877 | n/a |
| Dalbergia hupeana | Hance | Fabaceae:Papil. | Gutianshan | HQ427142 | HQ427296 | n/a |
| Daphniphyllum glaucescens subsp. oldhamii var. oldhamii | n/a | Daphniphyllaceae | Fushan | KJ688127 | KJ687547 | KJ686785 |
| Daphniphyllum oldhamii | (Hemsl.) K. Rosenthal | Daphniphyllaceae | Dinghushan | HQ427162 | HQ427311 | n/a |
| Daphnopsis philippiana | Krug & Urb. | Thymelaeaceae | Luquillo | HM446790 | HM446682 | HM446922 |
| Dehaasia cuneata | (Blume) Blume | Lauraceae | Bukit-Timah | n/a | n/a | BTSIN229-13 |
| Dendrobenthamia japonica | (Siebold & Zucc.) Hutch. | Cornaceae | Gutianshan | HQ427237.1 | HQ427380.1 | n/a |
| Dendropanax arboreus | (L.)Decne.&Planch. | Araliaceae | Luquillo | HM446791 | HM446683 | HM446923 |
| Dendropanax dentiger | (Harms) Merr. | Araliaceae | Gutianshan | HQ427251 | HQ427394 | n/a |
| Desmopsis panamensis | (B.L.Rob.)Saff. | Annonaceae | BCI | GQ981723 | GQ981981 | GQ982207 |
| Deutzia parviflora var amurensis | Regel | Hydrangeaceae | Changbaishan | CANGB017-14 | n/a | n/a |
| Dialium indum | L. | Fabaceae | Bukit-Timah | KJ594669 | n/a | BTSIN013-13 |
| Dillenia excelsa | Martelli ex Gilg | Dilleniaceae | Bukit-Timah | KJ594670 | KJ708879 | BTSIN287-13 |
| Dillenia grandifolia | Wall. ex Hook. f. & Thomson | Dilleniaceae | Bukit-Timah | KJ594671 | n/a | BTSIN207-13 |
| Dillenia suffruticosa | (Griff.) Martelli | Dilleniaceae | Bukit-Timah | n/a | KJ708881 | BTSIN634-13 |
| Dimocarpus longan | Lour. | Sapindaceae | Dinghushan | HQ415124 | HQ415302 | HQ415474 |
| Diospyros artanthifolia | Mart. | Ebenaceae | BCI | GQ981724 | GQ981982 | GQ982208 |
| Diospyros buxifolia | Thouars | Ebenaceae | Bukit-Timah | KJ594673 | KJ708884 | BTSIN349-13 |
| Diospyros clavigera | C.B. Clarke | Ebenaceae | Bukit-Timah | n/a | KJ708885 | BTSIN630-13 |
| Diospyros confusa | Bakh. | Ebenaceae | Bukit-Timah | KJ594674 | KJ708887 | BTSIN478-13 |
| Diospyros coriacea | Hiern | Ebenaceae | Bukit-Timah | n/a | KJ708889 | n/a |
| Diospyros eriantha | Champ. ex Benth. | Ebenaceae | Lienhuachih | KJ688132 | KJ687552 | KJ686790 |
| Diospyros glaucifolia | F.P. Metcalf | Ebenaceae | Gutianshan | HQ427239 | HQ427382 | n/a |
| Diospyros lanceifolia | Roxb. | Ebenaceae | Bukit-Timah | KJ594675 | KJ708890 | BTSIN133-13 |
| Diospyros maingayi | Bakh. | Ebenaceae | Bukit-Timah | KJ594677 | KJ708892 | BTSIN032-13 |
| Diospyros morrisiana | Hance in Walp. | Ebenaceae | Fushan | KJ688138 | NA | KJ686794 |
| Diospyros pilosanthera | Blanco | Ebenaceae | Bukit-Timah | KJ594678 | KJ708893 | BTSIN405-13 |
| Diospyros styraciformis | King & Gamble | Ebenaceae | Bukit-Timah | KJ594680 | KJ708898 | BTSIN002-13 |
| Diospyros venosa | Wall. ex A. DC. | Ebenaceae | Bukit-Timah | KJ594681 | KJ708900 | n/a |
| Diospyros virginiana | L. | Ebenaceae | SCBI | EU980774.1 | DQ924064.1 | n/a |
| Diplospora dubia | (Lindl.) Masam. | Rubiaceae | Dinghushan | HQ427201 | HQ427350 | HQ415437 |
| Diplospora malaccensis | Hook. f. | Rubiaceae | Bukit-Timah | KJ594682 | KJ708903 | BTSIN118-13 |
| Dipterocarpus caudatus | Foxw. | Dipterocarpaceae | Bukit-Timah | KJ594683 | KJ708904 | BTSIN234-13 |
| Dipterocarpus cf kunstleri | n/a | Dipterocarpaceae | Bukit-Timah | KJ594684 | n/a | BTSIN337-13 |
| Dipterocarpus cornutus | Dyer | Dipterocarpaceae | Bukit-Timah | n/a | KJ708905 | BTSIN046-13 |
| Dipterocarpus sublamellatus | Foxw. | Dipterocarpaceae | Bukit-Timah | n/a | KJ708906 | BTSIN579-13 |
| Dipterocarpus tempehes | Slooten | Dipterocarpaceae | Bukit-Timah | KJ594685 | KJ708907 | BTSIN051-13 |
| Dipteryx oleifera | Benth. | Fabaceae | BCI | GQ981725 | GQ981983 | GQ982209 |
| Dirca palustris | L. | Thymelaeaceae | Wabikon Lake | KJ593370 | KJ592930 | n/a |
| Distyliopsis dunnii | (Hemsl.) P.K. Endress | Hamamelidaceae | Lienhuachih | KJ688140 | NA | KJ686627 |
| Distylium myricoides | Hemsl. | Hamamelidaceae | Gutianshan | HQ427166 | n/a | n/a |
| Ditta myricoides | Griseb. | Euphorbiaceae | Luquillo | HM446792 | HM446684 | HM446924 |
| Dracaena maingayi | Hook. f. | Liliaceae | Bukit-Timah | KJ594686 | n/a | BTSIN016-13 |
| Drypetes alba | M¸ll. Arg. | Euphorbiaceae | Luquillo | HM446793 | n/a | HM446925 |
| Drypetes glauca | Vahl | Euphorbiaceae | Luquillo | HM446794 | n/a | HM446926 |
| Drypetes longifolia | (Blume) Pax & K. Hoffm. | Putranjivaceae | Bukit-Timah | KJ594687 | n/a | BTSIN270-13 |
| Drypetes pendula | Ridl. | Putranjivaceae | Bukit-Timah | KJ594688 | n/a | n/a |
| Durio griffithii | (Mast.) Bakh. | Malvaceae | Bukit-Timah | n/a | KJ708908 | BTSIN435-13 |
| Durio singaporensis | Ridl. | Malvaceae | Bukit-Timah | KJ594689 | KJ708910 | n/a |
| Dyera costulata | Hook. f. | Apocynaceae | Bukit-Timah | KJ594690 | KJ708911 | BTSIN134-13 |
| Dysoxylum acutangulum | Miq. | Meliaceae | Bukit-Timah | KJ594691 | KJ708912 | BTSIN050-13 |
| Dysoxylum cyrtobotryum | Miq. | Meliaceae | Bukit-Timah | KJ594692 | KJ708913 | BTSIN426-13 |
| Dysoxylum densiflorum | (Blume) Miq. | Meliaceae | Bukit-Timah | KJ594693 | KJ708914 | BTSIN299-13 |
| Ehretia acuminata | R. Br. | Boraginaceae | Gutianshan | HQ427271 | HQ427413 | n/a |
| Ehretia longiflora | Champ. ex Benth. | Boraginaceae | Fushan | KJ688142 | KJ687554 | KJ686796 |
| Elaeagnus umbellata | Thunb. | Elaeagnaceae | SCBI | HM849968.1 | HM851107.1 | HQ596679.1 |
| Elaeis oleifera | (Kunth)CortŽs | Arecaceae | BCI | GQ981726 | n/a | GQ982210 |
| Elaeocarpus chinensis | (Gardner & Champ.) Hook. f. ex Benth. | Elaeocarpaceae | Gutianshan | HQ427153 | HQ427304 | n/a |
| Elaeocarpus decipiens | Hemsl. | Elaeocarpaceae | Dinghushan | HQ427154 | HQ415261 | HQ415428 |
| Elaeocarpus ferrugineus | Steud. | Elaeocarpaceae | Bukit-Timah | KJ594695 | n/a | BTSIN409-13 |
| Elaeocarpus floribundus | Blume | Elaeocarpaceae | Bukit-Timah | KJ594696 | KJ708916 | BTSIN428-13 |
| Elaeocarpus japonicus | Siebold | Elaeocarpaceae | Fushan | KJ688145 | KJ687556 | KJ686799 |
| Elaeocarpus nitentifolius | Merr. & Chun | Elaeocarpaceae | Dinghushan | HQ415078 | HQ415262 | HQ415429 |
| Elaeocarpus nitidus | Jack | Elaeocarpaceae | Bukit-Timah | KJ594699 | KJ708919 | BTSIN087-13 |
| Elaeocarpus petiolatus | (Jack) Wall. | Elaeocarpaceae | Bukit-Timah | KJ594700 | KJ708920 | BTSIN414-13 |
| Elaeocarpus polystachyus | Wall. ex Müll. Berol. | Elaeocarpaceae | Bukit-Timah | n/a | KJ708921 | n/a |
| Elaeocarpus sylvestris | (Lour.) Poir. | Elaeocarpaceae | Dinghushan | HQ415081 | HQ415265 | HQ415432 |
| Eleutherococcus senticosus | (Rupr. ex Maxim.) Maxim. | Araliaceae | Changbaishan | CANGB018-14 | n/a | n/a |
| Ellipanthus tomentosus | Kurz | Connaraceae | Bukit-Timah | KJ594701 | KJ708922 | n/a |
| Endiandra maingayi | Hook. f. | Lauraceae | Bukit-Timah | n/a | KJ708924 | BTSIN652-13 |
| Endospermum diadenum | (Miq.) Airy Shaw | Euphorbiaceae | Bukit-Timah | KJ594703 | KJ708925 | BTSIN059-13 |
| Engelhardtia roxburghiana | Lindl. | Juglandaceae | Fushan | KJ688158 | KJ687562 | KJ686811 |
| Enkianthus quinqueflorus | Lour. | Ericaceae | Dinghushan | HQ415063 | HQ415251 | HQ415415 |
| Enterolobium schomburgkii | (Benth.)Benth. | Fabaceae | BCI | GQ981727 | GQ981984 | GQ982211 |
| Eriobotrya deflexa | (Hemsl.) Nakai | Rosaceae | Fushan | KJ688166 | NA | KJ686818 |
| Erythrina costaricensis | Micheli | Fabaceae | BCI | n/a | GQ981985 | GQ982212 |
| Erythrophleum fordii | Oliv. | Fabaceae:Papil. | Dinghushan | HQ415085 | HQ415268 | HQ415436 |
| Erythroxylum macrophyllum | Cav. | Erythroxylaceae | BCI | GQ981728 | GQ981986 | GQ982213 |
| Erythroxylum panamense | Turcz. | Erythroxylaceae | BCI | GQ981729 | GQ981987 | GQ982214 |
| Eugenia coloradoensis | Standl. | Myrtaceae | BCI | GQ981730 | GQ981988 | GQ982215 |
| Eugenia domingensis | O. Berg | Myrtaceae | Luquillo | HM446795 | HM446685 | HM446927 |
| Eugenia eggersii | Kiaersk. | Myrtaceae | Luquillo | HM446796 | HM446686 | HM446928 |
| Eugenia galalonensis | (C.WrightexGriseb.)Krug&Urb. | Myrtaceae | BCI | GQ981731 | n/a | n/a |
| Eugenia nesiotica | Standl. | Myrtaceae | BCI | GQ981732 | GQ981989 | GQ982216 |
| Eugenia oerstediana | O.Berg | Myrtaceae | BCI | GQ981733 | GQ981990 | n/a |
| Eugenia stahlii | (Kiaersk.) Krug & Urb. | Myrtaceae | Luquillo | HM446797 | HM446687 | HM446929 |
| Euonymus carnosus | Hemsl. | Celastraceae | Gutianshan | HQ427246 | HQ427389 | n/a |
| Euonymus laxiflorus | Champ. ex Benth. | Celastraceae | Lienhuachih | KJ688171 | KJ687571 | KJ686824 |
| Euonymus myrianthus | Hemsl. | Celastraceae | Gutianshan | HQ427245 | HQ427388 | n/a |
| Euonymus nitidus | Benth. | Celastraceae | Dinghushan | HQ427248 | HQ427391 | HQ415545 |
| Euonymus oblongifolius | Loes. & Rehder | Celastraceae | Gutianshan | HQ427248.1 | HQ427391.1 | n/a |
| Euonymus phellomana | Loes. ex Diels | Celastraceae | Changbaishan | CANGB019-14 | n/a | n/a |
| Euonymus tashiroi | Maxim. | Celastraceae | Lienhuachih | KJ688172 | KJ687572 | KJ686825 |
| Euonymus verrucosus | Scop. | Celastraceae | Changbaishan | CANGB020-14 | n/a | n/a |
| Eurya chinensis | R. Br. | Pentaphylacaceae | Dinghushan | HQ415122 | HQ415300 | HQ415472 |
| Eurya loquaiana | Dunn | Pentaphylacaceae | Fushan | KJ688176 | NA | KJ686830 |
| Eurya macartneyi | Champ. | Pentaphylacaceae | Dinghushan | HQ415121 | HQ415299 | HQ415471 |
| Eurya muricata | Dunn | Pentaphylacaceae | Gutianshan | HQ427228 | HQ427373 | n/a |
| Eurya rubiginosa | Hung T. Chang | Pentaphylacaceae | Gutianshan | HQ427222.1 | n/a | n/a |
| Eurycoma longifolia | Jack | Simaroubaceae | Bukit-Timah | KJ594704 | KJ708926 | BTSIN269-13 |
| Euscaphis japonica | (Thunb.) Dippel | Staphyleaceae | Gutianshan | HQ427180 | HQ427328 | n/a |
| Eustigma balansae | Oliv. | Hamamelidaceae | Dinghushan | HQ415214.1 | HQ415379.1 | HQ415561.1 |
| Eustigma oblongifolium | Gardner & Champ. | Hamamelidaceae | Lienhuachih | KJ688184 | KJ687581 | KJ686839 |
| Evodia faugeaii |  | Rutaceae | Gutianshan | KF912881.1 | n/a | n/a |
| Fagraea fragrans | Roxb. ex Carey & Wall. | Gentianaceae | Bukit-Timah | KJ594706 | n/a | n/a |
| Fagus grandifolia | Ehrh. | Fagaceae | SERC | KJ593410 | KJ592948 | n/a |
| Fagus sylvatica | L. | Fagaceae | Wytham | JN641795.1 | JN895059.1 | n/a |
| Faramea occidentalis | (L.)A.Rich. | Rubiaceae | Luquillo | HM446798 | HM446688 | HM446930 |
| Ficus ampelas | K.D. Koenig ex Roxb. | Moraceae | Lienhuachih | KJ688186 | NA | KJ686841 |
| Ficus bullenei | I.M.Johnst. | Moraceae | BCI | GQ981735 | GQ981991 | GQ982218 |
| Ficus citrifolia | Mill. | Moraceae | Luquillo | HM446799 | HM446689 | HM446931 |
| Ficus costaricana | (Liebm.)Miq. | Moraceae | BCI | GQ981737 | GQ981993 | GQ982220 |
| Ficus crassinervia | Desf. ex Willd. | Moraceae | Luquillo | HM446800 | HM446690 | HM446932 |
| Ficus erecta | Thunb. | Moraceae | Gutianshan | HQ427220.1 | n/a | n/a |
| Ficus erecta var. beecheyana | (Hook.&Arn.)King | Moraceae | Fushan | KJ688187 | KJ687582 | KJ686842 |
| Ficus esquiroliana | H.Lév. | Moraceae | Dinghushan | HQ415152 | HQ415326 | HQ415502 |
| Ficus fistulosa | Reinw. ex Blume | Moraceae | Lienhuachih | KJ688191 | NA | KJ686845 |
| Ficus formosana | Maxim. | Moraceae | Fushan | KJ688197 | KJ687585 | KJ686851 |
| Ficus grossularioides | Burm.f. | Moraceae | Bukit-Timah | KJ594707 | KJ708927 | n/a |
| Ficus hirta | Vahl | Moraceae | Dinghushan | HQ415157 | HQ415330 | HQ415506 |
| Ficus insipida | Willd. | Moraceae | BCI | GQ981738 | GQ981994 | GQ982221 |
| Ficus lamponga | Miq. | Moraceae | Bukit-Timah | n/a | n/a | BTSIN132-13 |
| Ficus maxima | Mill. | Moraceae | BCI | GQ981739 | GQ981995 | GQ982222 |
| Ficus nervosa | B. Heyne ex Roth | Moraceae | Lienhuachih | KJ688206 | KJ687592 | KJ686860 |
| Ficus obtusifolia | Kunth | Moraceae | BCI | GQ981740 | GQ981996 | GQ982223 |
| Ficus pandurata | Hance | Moraceae | Dinghushan | HQ415153 | HQ415327 | HQ415503 |
| Ficus popenoei | Standl. | Moraceae | BCI | GQ981741 | GQ981997 | GQ982224 |
| Ficus scortechinii | King | Moraceae | Bukit-Timah | n/a | KJ708928 | n/a |
| Ficus sintenisii | Warb. | Moraceae | Luquillo | HM446801 | HM446691 | HM446933 |
| Ficus sinuata | Thunb. | Moraceae | Bukit-Timah | KJ594709 | KJ708929 | BTSIN461-13 |
| Ficus superba | (Miq.) Miq. | Moraceae | Dinghushan | HQ415150.1 | HQ415324.1 | HQ415500.1 |
| Ficus superba var. japonica | Miq. | Moraceae | Fushan | KJ688208 | NA | NA |
| Ficus tonduzii | Standl. | Moraceae | BCI | GQ981742 | GQ981998 | GQ982225 |
| Ficus trigonata | L. | Moraceae | BCI | GQ981743 | n/a | GQ982226 |
| Ficus variegata | Blume | Moraceae | Dinghushan | HQ415154 | n/a | n/a |
| Ficus variolosa | Lindl. ex Benth. | Moraceae | Dinghushan | HQ415151 | HQ415325 | HQ415501 |
| Ficus vasculosa | Wall. ex Miq. | Moraceae | Dinghushan | HQ415149 | HQ415323 | HQ415499 |
| Ficus yoponensis | Desv. | Moraceae | BCI | GQ981744 | GQ981999 | GQ982227 |
| Flacourtia rukam | Zoll. & Moritzi | Salicaceae | Bukit-Timah | n/a | JF738593.1 | BTSIN626-13 |
| Flueggea virosa | (Roxb. ex Willd.) Royle | Phyllanthaceae | Dinghushan | HQ415223 | HQ415387 | HQ415569 |
| Frangula alnus | Mill. | Rhamnaceae | Wytham | EU676982.1 | EU749374.1 | EU750523.1 |
| Fraxinus americana | L. | Oleaceae | Wabikon Lake | KJ593422 | KJ592959 | n/a |
| Fraxinus excelsior | L. | Oleaceae | Wytham | FJ862056.1 | FJ395414.1 | FJ395513.1 |
| Fraxinus insularis | Hemsl. | Oleaceae | Gutianshan | HQ427187 | HQ427335 | n/a |
| Fraxinus mandshurica | Rupr. | Oleaceae | Changbaishan | CANGB021-14 | n/a | n/a |
| Fraxinus nigra | Marshall | Oleaceae | Wabikon Lake | KJ593423 | KJ592960 | n/a |
| Fraxinus pennsylvanica | Marshall | Oleaceae | SERC | HQ590104.1 | HQ593301.1 | HQ596703.1 |
| Gaertnera grisea | Hook. f. ex C.B. Clarke | Rubiaceae | Bukit-Timah | n/a | AM117227.1 | BTSIN639-13 |
| Galearia fulva | (Tul.) Miq. | Pandaceae | Bukit-Timah | KJ594713 | KJ708930 | n/a |
| Galearia maingayi | Hook. f. | Pandaceae | Bukit-Timah | KJ594714 | KJ708931 | n/a |
| Garcinia atroviridis | Griff. ex T. Anderson | Clusiaceae | Bukit-Timah | KJ594716 | n/a | BTSIN004-13 |
| Garcinia eugeniifolia | Wall. ex T. Anderson | Clusiaceae | Bukit-Timah | KJ594720 | n/a | n/a |
| Garcinia forbesii | King | Clusiaceae | Bukit-Timah | KJ594717 | n/a | BTSIN418-13 |
| Garcinia hombroniana | Pierre | Clusiaceae | Bukit-Timah | KJ594718 | KJ708932 | BTSIN295-13 |
| Garcinia intermedia | (Pittier)Hammel | Clusiaceae | BCI | GQ981745 | n/a | GQ982228 |
| Garcinia madruno | (Kunth)Hammel | Clusiaceae | BCI | GQ981746 | n/a | GQ982229 |
| Garcinia multiflora | Champ. ex Benth. | Clusiaceae | Nanjenshan | HQ415192.1 | n/a | HQ415541.1 |
| Garcinia oblongifolia | Champ. ex Benth. | Clusiaceae | Dinghushan | HQ415193 | n/a | n/a |
| Garcinia scortechinii | King | Clusiaceae | Bukit-Timah | KJ594720 | n/a | BTSIN381-13 |
| Gardenia jasminoides | J. Ellis | Rubiaceae | Lienhuachih | KJ688214 | KJ687596 | KJ686866 |
| Gardenia tubifera | Wall. ex Roxb. | Rubiaceae | Bukit-Timah | KJ594722 | KJ708933 | BTSIN449-13 |
| Genipa americana | L. | Rubiaceae | Luquillo | HM446802 | HM446692 | HM446934 |
| Gironniera subaequalis | Planch. | Cannabaceae | Dinghushan | HQ415052 | HQ415240 | HQ415404 |
| Glochidion acuminatum | M¸ll. Arg. | Phyllanthaceae | Fushan | KJ688215 | NA | NA |
| Glochidion eriocarpum | Champ. ex Benth. | Phyllanthaceae | Dinghushan | HQ415188 | HQ415358 | HQ415537 |
| Glochidion hypoleucum | Hayata | Phyllanthaceae | Bukit-Timah | KJ594724 | n/a | BTSIN174-13 |
| Glochidion puberulum | Hosok. | Phyllanthaceae | Gutianshan | HQ427128.1 | n/a | n/a |
| Glochidion wrightii | Benth. | Phyllanthaceae | Dinghushan | HQ415187 | HQ415357 | HQ415536 |
| Gluta malayana | Ding Hou | Anacardiaceae | Bukit-Timah | KJ594725 | KJ708936 | BTSIN028-13 |
| Gluta wallichii | Ding Hou | Anacardiaceae | Bukit-Timah | KJ594726 | KJ708937 | BTSIN201-13 |
| Glycosmis chlorosperma | Spreng. | Rutaceae | Bukit-Timah | KJ594728 | KJ708939 | BTSIN360-13 |
| Glycosmis citrifolia | (Willd.) Lindl. | Rutaceae | Lienhuachih | KJ688225 | KJ687602 | KJ686875 |
| Glycosmis parviflora | (Sims) Little | Rutaceae | Dinghushan | HQ415179 | HQ415350 | HQ415528 |
| Gnetum_gnemon | L. | Gnetaceae | Bukit-Timah | AY296534.1 | n/a | n/a |
| Gomphandra quadrifida | (Blume) Sleumer | Stemonuraceae | Bukit-Timah | KJ594729 | KJ708940 | BTSIN346-13 |
| Gomphia serrata | (Gaertn.) Kanis | Ochnaceae | Bukit-Timah | KJ594730 | n/a | BTSIN015-13 |
| Goniothalamus macrophyllus | Hook. f. & Thomson | Annonaceae | Bukit-Timah | n/a | KJ708941 | BTSIN267-13 |
| Goniothalamus tapis | Miq. | Annonaceae | Bukit-Timah | n/a | KJ708941 | n/a |
| Gonystylus confusus | Airy Shaw | Thymelaeaceae | Bukit-Timah | KJ594731 | KJ708943 | BTSIN357-13 |
| Gonzalagunia spicata | (Lam.) M. Gómez | Rubiaceae | Luquillo | HM446803 | HM446693 | HM446935 |
| Gordonia axillaris | (Roxb. ex Ker Gawl.) D. Dietr. | Theaceae | Fushan | KJ688226 | NA | KJ686876 |
| Gordonia singaporeana | (Dyer) Wall. ex Ridl. | Theaceae | Bukit-Timah | KJ594733 | KJ708945 | BTSIN142-13 |
| Guapira standleyana | Woodson | Nyctaginaceae | BCI | GQ981748 | GQ982001 | GQ982231 |
| Guarea bullata | Radlk. | Meliaceae | BCI | JQ592719.1 | JQ588338.1 | n/a |
| Guarea fuzzy | n/a | Meliaceae | BCI | GQ981749 | n/a | GQ982232 |
| Guarea glabra | Vahl | Meliaceae | Luquillo | HM446804 | HM446694 | HM446936 |
| Guarea grandifolia | DC. | Meliaceae | BCI | GQ981750 | GQ982002 | GQ982233 |
| Guarea guidonia | (L.)Sleumer | Meliaceae | Luquillo | HM446805 | HM446695 | HM446937 |
| Guatteria caribaea | Urb. | Annonaceae | Luquillo | HM446806 | HM446696 | HM446938 |
| Guatteria dumetorum | R.E.Fr. | Annonaceae | BCI | GQ981752 | n/a | GQ982235 |
| Guazuma ulmifolia | Lam. | Sterculiaceae | Luquillo | HM446807 | n/a | HM446939 |
| Guettarda foliacea | Standl. | Rubiaceae | BCI | GQ981754 | GQ982004 | GQ982237 |
| Guettarda valenzuelana | A. Rich. | Rubiaceae | Luquillo | HM446808 | HM446697 | HM446940 |
| Guioa pubescens | Radlk. | Sapindaceae | Bukit-Timah | KJ594735 | KJ708947 | BTSIN411-13 |
| Gustavia superba | (Kunth)O.Berg | Lecythidaceae | BCI | GQ981755 | GQ982005 | GQ982238 |
| Gymnacranthera farquhariana | Warb. | Myristicaceae | Bukit-Timah | KJ594739 | KJ708949 | BTSIN482-13 |
| Gynotroches axillaris | Blume | Rhizophoraceae | Bukit-Timah | KJ594741 | n/a | n/a |
| Hamamelis virginiana | L. | Hamamelidaceae | SCBI | DQ352368.1 | n/a | EU595863.1 |
| Hamelia axillaris | Sw. | Rubiaceae | Luquillo | HM446809 | n/a | n/a |
| Hamelia patens | Jacq. | Rubiaceae | BCI | GQ981757 | GQ982007 | GQ982240 |
| Hampea appendiculata | (Donn.Sm.)Standl. | Malvaceae | BCI | GQ981758 | n/a | GQ982241 |
| Hasseltia floribunda | Kunth | Salicaceae | BCI | GQ981759 | GQ982008 | GQ982242 |
| Heisteria acuminata | (Bonpl.)Engl. | Olacaceae | BCI | GQ981760 | GQ982009 | GQ982243 |
| Heisteria concinna | Standl. | Olacaceae | BCI | GQ981761 | GQ982010 | GQ982244 |
| Heisteria_scandens | Ducke | Olacaceae | BCI | JQ593018.1 | JQ588534.1 | n/a |
| Helicia cochinchinensis | Lour. | Proteaceae | Lienhuachih | KJ688230 | NA | KJ686880 |
| Helicia formosana | Hemsl. | Proteaceae | Fushan | NA | KJ687603 | NA |
| Helicia rengetiensis | Masam. | Proteaceae | Lienhuachih | KJ688232 | KJ687605 | KJ686881 |
| Helicia reticulata | W.T. Wang | Proteaceae | Dinghushan | HQ415175 | HQ415346 | HQ415524 |
| Henriettea fascicularis | (Sw.) M. Gómez | Melastomataceae | Luquillo | HM446810 | HM446698 | HM446941 |
| Heritiera borneensis | (Merr.) Kosterm. | Malvaceae | Bukit-Timah | KJ594742 | KJ708951 | BTSIN423-13 |
| Heritiera elata | Ridl. | Malvaceae | Bukit-Timah | n/a | KJ708952 | BTSIN176-13 |
| Herrania purpurea | (Pittier)R.E.Schult. | Malvaceae | BCI | GQ981762 | GQ982011 | GQ982245 |
| Hevea brasiliensis | (Willd.exA.Juss.)MŸll.Arg. | Euphorbiaceae | Bukit-Timah | KJ594743 | KJ708953 | BTSIN306-13 |
| Hibiscus pernambucensis | Arruda | Malvaceae | Luquillo | HM446811 | HM446699 | HM446943 |
| Hieronyma alchorneoides | Allem‹o | Euphorbiaceae | BCI | n/a | GQ982012 | GQ982246 |
| Hirtella americana | L. | Chrysobalanaceae | BCI | GQ981763 | n/a | GQ982247 |
| Hirtella rugosa | Thuill. ex Pers. | Chrysobalanaceae | Luquillo | HM446812 | n/a | HM446944 |
| Hirtella triandra | Sw. | Chrysobalanaceae | BCI | GQ981764 | n/a | GQ982248 |
| Homalium cochinchinense | Druce | Salicaceae | Dinghushan | HQ415194 | HQ415362 | HQ415542 |
| Homalium cochinchinensis | (Lour.) Druce | Salicaceae | Lienhuachih | KJ688234 | KJ687606 | KJ686883 |
| Homalium racemosum | Jacq. | Flacourtiaceae | Luquillo | HM446813 | HM446700 | HM446945 |
| Hopea ferrugineum | n/a | Dipterocarpaceae | Bukit-Timah | KJ594745 | n/a | n/a |
| Hopea griffithii | Kurz | Dipterocarpaceae | Bukit-Timah | n/a | KJ708954 | BTSIN625-13 |
| Hopea mengarawan | Miq. | Dipterocarpaceae | Bukit-Timah | KJ594746 | KJ708955 | BTSIN084-13 |
| Hopea sangal | Korth. | Dipterocarpaceae | Bukit-Timah | KJ594747 | KJ708957 | BTSIN058-13 |
| Horsfieldia polyspherula | J. Sinclair | Myristicaceae | Bukit-Timah | KJ594749 | KJ708958 | BTSIN023-13 |
| Horsfieldia wallichii | Warb. | Myristicaceae | Bukit-Timah | KJ594752 | KJ708961 | BTSIN177-13 |
| Hovenia acerba | Lindl. | Rhamnaceae | Dinghushan | HQ415232 | HQ415396 | HQ415578 |
| Hovenia trichocarpa | Chun & Tsiang | Rhamnaceae | Gutianshan | HQ427241.1 | n/a | n/a |
| Hura crepitans | L. | Euphorbiaceae | BCI | GQ981765 | GQ982013 | GQ982249 |
| Hybanthus prunifolius | (Humb.&Bonpl.exSchult.)Schulze-Menz | Violaceae | BCI | GQ981766 | GQ982014 | GQ982250 |
| Hydrangea chinensis | Maxim. | Hydrangeaceae | Fushan | KJ688235 | NA | KJ686884 |
| Idesia polycarpa | Maxim. | Salicaceae | Gutianshan | HQ427272 | HQ427414 | n/a |
| Ilex aquifolium | L. | Aquifoliaceae | Wytham | FJ395601.1 | FJ395435.1 | FN675791.1 |
| Ilex asprella | (Hook. & Arn.) Champ. ex Benth. | Aquifoliaceae | Lienhuachih | KJ688236 | KJ687607 | KJ686885 |
| Ilex chapaensis | Merr. | Aquifoliaceae | Dinghushan | HQ415069 | HQ415254 | HQ415421 |
| Ilex chinensis | Sims | Aquifoliaceae | Gutianshan | HQ427140 | n/a | n/a |
| Ilex cochinchinensis | (Lour.) Loes. | Aquifoliaceae | Dinghushan | HQ415071 | HQ415256 | HQ415423 |
| Ilex elmerrilliana | S.Y. Hu | Aquifoliaceae | Gutianshan | HQ427132.1 | n/a | n/a |
| Ilex ficoidea | Hemsl. | Aquifoliaceae | Fushan | KJ688237 | KJ687608 | KJ686886 |
| Ilex formosana | Maxim. | Aquifoliaceae | Fushan | KJ688248 | KJ687619 | KJ686897 |
| Ilex goshiensis | Hayata | Aquifoliaceae | Fushan | KJ688253 | KJ687624 | KJ686902 |
| Ilex hayataiana | n/a | Aquifoliaceae | Fushan | KJ688262 | KJ687633 | KJ686911 |
| Ilex latifolia | Thunb. | Aquifoliaceae | Gutianshan | HQ427134 | HQ427289 | n/a |
| Ilex lonicerifolia | Hayata | Aquifoliaceae | Fushan | KJ688263 | KJ687634 | KJ686912 |
| Ilex macrocarpa | Oliv. | Aquifoliaceae | Dinghushan | HQ415064 | n/a | HQ415416 |
| Ilex memecylifolia | Champ. ex Benth. | Aquifoliaceae | Dinghushan | HQ415065 | HQ415252 | HQ415417 |
| Ilex micrococca | Maxim. | Aquifoliaceae | Gutianshan | HQ427135 | HQ427290 | n/a |
| Ilex opaca | Aiton | Aquifoliaceae | SERC | EF590536.1 | GQ248140.1 | n/a |
| Ilex pubescens | Hook. & Arn. | Aquifoliaceae | Fushan | KJ688275 | KJ687646 | KJ686924 |
| Ilex rotunda | Thunb. | Aquifoliaceae | Dinghushan | HQ427138 | HQ415255 | HQ415422 |
| Ilex sideroxyloides | (Sw.) Griseb. | Aquifoliaceae | Luquillo | L01928.2* | n/a | n/a |
| Ilex suaveolens | (H. LÈv.) Loes. | Aquifoliaceae | Gutianshan | HQ427139 | HQ427293 | n/a |
| Ilex triflora | Blume | Aquifoliaceae | Dinghushan | HQ415068 | n/a | HQ415420 |
| Ilex verticillata | (L.) A. Gray | Aquifoliaceae | Wabikon Lake | KJ593485 | KJ592999 | n/a |
| Ilex wilsonii | Loes. | Aquifoliaceae | Gutianshan | HQ427140 | HQ427294 | n/a |
| Illicium lanceolatum | A.C. Sm. | Schisandraceae | Gutianshan | HQ427126 | HQ427283 | n/a |
| Indocalamus longiauritus | Hand.-Mazz. | Poaceae | Dinghushan | HQ415166 | HQ415338 | HQ415515 |
| Inga acuminata | Benth. | Fabaceae | BCI | GQ982015 | GQ981767 | GQ982251 |
| Inga cocleensis | Pittier | Fabaceae | BCI | GQ981768 | GQ982016 | GQ982252 |
| Inga goldmanii | Pittier | Fabaceae | BCI | GQ981769 | GQ982017 | GQ982253 |
| Inga laurina | (Sw.)Willd. | Fabaceae | BCI | HM446814 | HM446701 | HM446946 |
| Inga marginata | Willd. | Fabaceae | BCI | GQ981771 | GQ982018 | GQ982255 |
| Inga nobilis | Willd. | Fabaceae | BCI | GQ981772 | GQ982019 | GQ982256 |
| Inga oerstediana | Benth.exSeem. | Fabaceae | BCI | GQ981773 | n/a | GQ982257 |
| Inga pezizifera | Benth. | Fabaceae | BCI | GQ981774 | GQ982020 | GQ982258 |
| Inga punctata | Willd. | Fabaceae | BCI | GQ981775 | GQ982021 | GQ982259 |
| Inga ruiziana | G.Don | Fabaceae | BCI | GQ981776 | GQ982022 | GQ982260 |
| Inga sapindoides | Willd. | Fabaceae | BCI | GQ981777 | GQ982023 | GQ982261 |
| Inga spectabilis | (Vahl)Willd. | Fabaceae | BCI | GQ981778 | GQ982024 | GQ982262 |
| Inga thibaudiana | DC. | Fabaceae | BCI | GQ981779 | GQ982025 | GQ982263 |
| Inga umbellifera | (Vahl)Steud. | Fabaceae | BCI | GQ981780 | GQ982026 | GQ982264 |
| Inga vera | Willd. | Fabaceae:Mimos. | Luquillo | HM446815 | HM446702 | HM446947 |
| Irvingia malayana | Oliver ex Bennett | Irvingiaceae | Bukit-Timah | KJ594753 | KJ708962 | n/a |
| Itea chinensis | Hook. & Arn. | Iteaceae | Dinghushan | HQ415186 | HQ415356 | HQ415535 |
| Itea oblonga | Hand.-Mazz. | Iteaceae | Gutianshan | HQ427159.1 | n/a | n/a |
| Itea parviflora | Hemsl. | Iteaceae | Fushan | KJ688283 | NA | NA |
| Ixonanthes reticulata | Jack | Ixonanthaceae | Bukit-Timah | n/a | n/a | n/a |
| Ixora chinensis | Lam. | Rubiaceae | Dinghushan | HQ415123 | HQ415301 | HQ415473 |
| Ixora ferrea | (Jacq.) Benth. | Rubiaceae | Luquillo | HM446816 | HM446703 | HM446948 |
| Ixora javanica | (Blume) DC. | Rubiaceae | Bukit-Timah | KJ594754 | KJ708963 | BTSIN271-13 |
| Ixora pendula | Jack | Rubiaceae | Bukit-Timah | KJ594757 | KJ708966 | BTSIN066-13 |
| Jacaranda copaia | (Aubl.)D.Don | Bignoniaceae | BCI | GQ981781 | n/a | GQ982265 |
| Juglans cinerea | L. | Juglandaceae | Wabikon Lake | HQ590142.1 | HQ594030.1 | n/a |
| Juglans mandshurica | Maxim. | Juglandaceae | Changbaishan | CANGB022-14 | n/a | n/a |
| Juglans nigra | L. | Juglandaceae | SCBI | U00437.1 | U92851.1 | n/a |
| Juniperus virginiana | L. | Cupressaceae | SCBI | NC_024023 | NC_024023 | NC_024023 |
| Knema cinerea | Warb. | Myristicaceae | Bukit-Timah | KJ594758 | KJ708967 | BTSIN199-13 |
| Knema hookeriana | Warb. | Myristicaceae | Bukit-Timah | KJ594759 | KJ708968 | BTSIN417-13 |
| Knema laurina | Warb. | Myristicaceae | Bukit-Timah | KJ594761 | KJ708970 | BTSIN318-13 |
| Knema patentinervia | (J.Sinclair) W.J.de Wilde | Myristicaceae | Bukit-Timah | KJ594762 | KJ708971 | BTSIN597-13 |
| Koilodepas longifolium | Hook. f. | Euphorbiaceae | Bukit-Timah | KJ594763 | n/a | BTSIN362-13 |
| Kokoona reflexa | (M.A.Lawson) Ding Hou | Celastraceae | Bukit-Timah | KJ594764 | KJ708972 | BTSIN075-13 |
| Koompassia malaccensis | Maing. | Fabaceae | Bukit-Timah | KJ594765 | KJ708973 | BTSIN293-13 |
| Lacistema aggregatum | (P.J.Bergius)Rusby | Lacistemataceae | BCI | n/a | GQ982027 | n/a |
| Lacmellea panamensis | (Woodson)Markgr. | Apocynaceae | BCI | GQ981782 | GQ982028 | GQ982266 |
| Laetia procera | (Poepp.)Eichler | Salicaceae | BCI | HM446817 | HM446704 | HM446949 |
| Laetia thamnia | L. | Salicaceae | BCI | GQ981784 | n/a | GQ982268 |
| Lafoensia punicifolia | DC. | Lythraceae | BCI | n/a | GQ982030 | GQ982269 |
| Lagerstroemia subcostata | Koehne | Lythraceae | Fushan | KJ688287 | NA | KJ686933 |
| Larix decidua | Mill. | Pinaceae | Wytham | NC_016058.1 | NC_016058.1 | NC_016058.1 |
| Larix gmelinii | (Rupr.) Kuzen. | Pinaceae | Changbaishan | CANGB023-14 | n/a | n/a |
| Larix laricina | (Du Roi) K. Koch | Pinaceae | Wabikon Lake | KJ593496 | n/a | n/a |
| Lasianthus appressihirtus var. maximus | SimizuexL.J.Liu | Rubiaceae | Fushan | KJ688296 | KJ687656 | KJ686941 |
| Lasianthus bunzanensis | Simizu | Rubiaceae | Lienhuachih | KJ688297 | NA | KJ686942 |
| Lasianthus chinensis | (Champ. ex Benth.) Benth. | Rubiaceae | Dinghushan | HQ415060 | HQ415248 | HQ415412 |
| Lasianthus curtisii | King & Gamble | Rubiaceae | Fushan | KJ688298 | KJ687657 | KJ686943 |
| Lasianthus cyanocarpus | Jack | Rubiaceae | Lienhuachih | KJ688307 | KJ687666 | KJ686952 |
| Lasianthus ellipticus | Wight | Rubiaceae | Bukit-Timah | BTSIN214-13 | BTSIN214-13 | BTSIN214-13 |
| Lasianthus fordii | Hance | Rubiaceae | Fushan | KJ688311 | KJ687670 | KJ686956 |
| Lasianthus japonicus | Miq. | Rubiaceae | Gutianshan | HQ427196 | HQ427345 | n/a |
| Lasianthus microstachys | Hayata | Rubiaceae | Fushan | KJ688316 | KJ687675 | KJ686961 |
| Lasianthus obliquinervis | Merr. | Rubiaceae | Lienhuachih | KJ688325 | KJ687683 | KJ686970 |
| Leandra dichotoma | (Pav.exD.Don)Cogn. | Melastomataceae | BCI | GQ981785 | GQ982031 | GQ982270 |
| Lepisanthes fruticosa | (Roxb.) Leenh. | Sapindaceae | Bukit-Timah | KJ594767 | KJ708974 | BTSIN205-13 |
| Lespedeza formosa | (Vogel) Koehne | Fabaceae | Gutianshan | HQ427143.1 | n/a | n/a |
| Licania hypoleuca | Benth. | Chrysobalanaceae | BCI | GQ981786 | GQ982032 | GQ982271 |
| Licania platypus | (Hemsl.)Fritsch | Chrysobalanaceae | BCI | GQ981787 | GQ982033 | GQ982272 |
| Licania splendens | (Korth.) Prance | Chrysobalanaceae | Bukit-Timah | KJ594768 | KJ708975 | BTSIN474-13 |
| Limlia uraiana | (Hayata) Masam. & Tomiya | Fagaceae | Fushan | KJ688332 | KJ687690 | KJ686979 |
| Lindackeria laurina | C.Presl | Achariaceae | BCI | GQ981788 | GQ982034 | GQ982273 |
| Lindera aggregata | (Sims) Kosterm. | Lauraceae | Gutianshan | HQ427260 | HQ427402 | n/a |
| Lindera benzoin | (L.) Blume | Lauraceae | SERC | AY337732.1 | AJ247169.2 | n/a |
| Lindera chunii | Merr. | Lauraceae | Dinghushan | HQ415171 | HQ415342 | HQ415520 |
| Lindera communis | Hemsl. | Lauraceae | Fushan | KJ688337 | KJ687695 | KJ686984 |
| Lindera glauca | (Siebold & Zucc.) Blume | Lauraceae | Gutianshan | HQ427265 | HQ427407 | n/a |
| Lindera metcalfiana | C.K. Allen | Lauraceae | Dinghushan | HQ415172 | HQ415343 | HQ415521 |
| Lindera reflexa | Hemsl. | Lauraceae | Gutianshan | HQ427264 | HQ427406 | n/a |
| Liquidambar formosana | Hance | Altingiaceae | Gutianshan | HQ427164 | HQ427313 | n/a |
| Liquidambar styraciflua | L. | Altingiaceae | SERC | AF119181.1 | AF133218.1 | AB445366.1 |
| Liriodendron tulipifera | L. | Magnoliaceae | SERC | AY008947.1 | n/a | AB021047.1 |
| Litchi chinensis | Sonn. | Sapindaceae | Dinghushan | HQ415120 | HQ415298 | HQ415470 |
| Lithocarpus amygdalifolius | (Skan) Hayata | Fagaceae | Lienhuachih | KJ688347 | KJ687704 | KJ686993 |
| Lithocarpus glaber | (Thunb.) Nakai | Fagaceae | Gutianshan | HQ427174 | HQ427322 | n/a |
| Lithocarpus gracilis | (Korth.) Soepadmo | Fagaceae | Bukit-Timah | n/a | KJ708976 | n/a |
| Lithocarpus sundaicus | (Blume) Rehder | Fagaceae | Bukit-Timah | n/a | KJ708977 | n/a |
| Litsea accedens | (Blume) Boerl. | Lauraceae | Bukit-Timah | n/a | KJ708978 | BTSIN660-13 |
| Litsea acuminata | (Blume) Kurata | Lauraceae | Fushan | KJ688350 | NA | KJ686996 |
| Litsea castanea | Hook. f. | Lauraceae | Bukit-Timah | n/a | KJ708981 | BTSIN390-13 |
| Litsea coreana | H.Lév. | Lauraceae | Gutianshan | HQ427263.1 | n/a | n/a |
| Litsea costalis | (Nees) Kosterm. | Lauraceae | Bukit-Timah | KJ594769 | KJ708982 | BTSIN073-13 |
| Litsea cubeba | (Lour.) Pers. | Lauraceae | Lienhuachih | KJ688359 | KJ687710 | KJ687004 |
| Litsea elliptica | Blume | Lauraceae | Bukit-Timah | KJ594770 | KJ708983 | BTSIN244-13 |
| Litsea elongata | (Nees) Hook. f. | Lauraceae | Gutianshan | HQ427261 | HQ427403 | n/a |
| Litsea firma | (Blume) Hook. f. | Lauraceae | Bukit-Timah | KJ594773 | n/a | n/a |
| Litsea grandis | (Nees) Hook. f. | Lauraceae | Bukit-Timah | KJ594774 | KJ708986 | n/a |
| Litsea hypophaea | Hayata | Lauraceae | Lienhuachih | KJ688361 | KJ687711 | KJ687006 |
| Litsea ridleyi | Gamble | Lauraceae | Bukit-Timah | KJ594775 | KJ708987 | BTSIN204-13 |
| Litsea rotundifolia | (Nees) C.K. Allen | Lauraceae | Dinghushan | HQ415128 | HQ415306 | HQ415478 |
| Litsea verticillata | Hance | Lauraceae | Dinghushan | HQ415129 | n/a | HQ415479 |
| Lonchocarpus heptaphyllus | (Poir.)DC. | Fabaceae | BCI | GQ981789 | GQ982035 | GQ982274 |
| Lonchocarpus latifolius | DC. | Fabaceae:Papil. | Luquillo | HM446818 | HM446705 | HM446950 |
| Lonicera praeflorens | Batalin | Caprifoliaceae | Changbaishan | CANGB024-14 | n/a | n/a |
| Lophopetalum pallidum | M.A.Lawson | Celastraceae | Bukit-Timah | KJ594776 | n/a | BTSIN068-13 |
| Lophopetalum wightianum | Arn. | Celastraceae | Bukit-Timah | KJ594776 | KJ708988 | BTSIN049-13 |
| Loropetalum chinensis | (R. Br.) Oliv. | Hamamelidaceae | Gutianshan | HQ427163.1 | n/a | n/a |
| Lozania pittieri | (S.F.Blake)L.B.Sm. | Lacistemataceae | BCI | GQ981790 | n/a | GQ982275 |
| Ludwigia octovalvis | (Jacq.) P.H. Raven | Onagraceae | Luquillo | L1022* | n/a | n/a |
| Luehea seemannii | Triana&Planch. | Malvaceae | BCI | GQ981791 | GQ982036 | GQ982276 |
| Lyonia ovalifolia | (Wall.) Drude | Ericaceae | Gutianshan | HQ427150.1 | n/a | n/a |
| Maackia amurensis | Rupr. | Fabaceae | Changbaishan | CANGB025-14 | n/a | n/a |
| Macaranga andamanica | Kurz | Euphorbiaceae | Dinghushan | HQ415129 | n/a | HQ415479 |
| Macaranga bancana | (Miq.) Müll. Arg. | Euphorbiaceae | Bukit-Timah | KJ594777 | n/a | n/a |
| Macaranga lowii | King ex Hook. f. | Euphorbiaceae | Bukit-Timah | KJ594780 | n/a | n/a |
| Macaranga sampsonii | Hance | Euphorbiaceae | Dinghushan | HQ415215 | HQ415380 | HQ415562 |
| Machilus breviflora | (Benth.) Hemsl. | Lauraceae | Dinghushan | HQ415159 | HQ415332 | HQ415508 |
| Machilus chinensis | (Benth.) Hemsl. | Lauraceae | Dinghushan | HQ415162 | HQ415335 | HQ415511 |
| Machilus grijsii | Hance | Lauraceae | Dinghushan | KF569893.1 | KF740400.1 | n/a |
| Machilus japonica var. kusanoi | (Hayata)J.C.Liao | Lauraceae | Fushan | KJ688368 | NA | KJ687012 |
| Machilus kwangtungensis | Yen C. Yang | Lauraceae | Dinghushan | HQ415161 | HQ415334 | HQ415510 |
| Machilus pauhoi | Kaneh. | Lauraceae | Gutianshan | HQ427276 | HQ427418 | n/a |
| Machilus phoenicis | Dunn | Lauraceae | Dinghushan | HQ415160 | HQ415333 | HQ415509 |
| Machilus robusta | W.W. Sm. | Lauraceae | Dinghushan | HQ415164.1 | HQ415336.1 | HQ415513.1 |
| Machilus thunbergii | Siebold & Zucc. & Zucc. | Lauraceae | Fushan | KJ688372 | NA | KJ687017 |
| Machilus velutina | Champ. ex Benth. | Lauraceae | Dinghushan | HQ415165 | HQ415337 | HQ415514 |
| Machilus zuihoensis | Hayata | Lauraceae | Fushan | KJ688384 | NA | KJ687031 |
| Maclura cochinchinensis | (Lour.) Corner | Moraceae | Gutianshan | HQ427219 | HQ427365 | n/a |
| Maclurodendron porteri | (Hook. f.) T.G. Hartley | Rutaceae | Bukit-Timah | KJ594781 | n/a | n/a |
| Macrocnemum roseum | (Ruiz&Pav.)Wedd. | Rubiaceae | BCI | GQ981792 | GQ982037 | GQ982278 |
| Madhuca kingiana | (Brace ex King & Gamble) H.J. Lam | Sapotaceae | Bukit-Timah | KJ594782 | KJ708989 | BTSIN217-13 |
| Madhuca korthalsii | (Pierre ex Burck) H.J. Lam | Sapotaceae | Bukit-Timah | KJ594783 | KJ708990 | BTSIN096-13 |
| Madhuca malaccensis | (C.B. Clarke) H.J. Lam | Sapotaceae | Bukit-Timah | KJ594784 | KJ708991 | BTSIN041-13 |
| Maesa japonica | Merr. | Primulaceae | Fushan | KJ688389 | KJ687721 | KJ687035 |
| Maesa perlaria var. formosana | (Mez)YuenP.Yang | Primulaceae | Fushan | KJ688390 | KJ687722 | KJ687036 |
| Maesa ramentacea | (Roxb.) A. DC. | Primulaceae | Bukit-Timah | n/a | KJ708993 | BTSIN328-13 |
| Maesa salicifolia | E. Walker | Primulaceae | Dinghushan | HQ415072 | HQ415257 | HQ415424 |
| Magnolia cylindrica | E.H. Wilson | Magnoliaceae | Gutianshan | HQ427278 | HQ427420 | n/a |
| Magnolia elegans | (Blume) H. Keng | Magnoliaceae | Bukit-Timah | KJ594786 | KJ708994 | BTSIN006-13 |
| Magnolia paenetalauma | Dandy | Magnoliaceae | Dinghushan | HQ415131 | HQ415308 | HQ415481 |
| Magnolia splendens | Urb. | Magnoliaceae | Luquillo | HM446819 | n/a | HM446951 |
| Mahonia bealei | (Fortune) CarriËre | Berberidaceae | Gutianshan | L75871.2 | n/a | n/a |
| Mallotus apelta | (Lour.) M¸ll. Arg. | Euphorbiaceae | Dinghushan | HQ427129 | HQ427286 | HQ415565 |
| Mallotus hookerianus | (Seem.) M¸ll. Arg. | Euphorbiaceae | Dinghushan | HQ415222 | HQ415386 | HQ415568 |
| Mallotus japonicus | (Spreng.) M¸ll. Arg. | Euphorbiaceae | Fushan | KJ688398 | NA | KJ686635 |
| Mallotus paniculatus | (Lam.) M¸ll. Arg. | Euphorbiaceae | Fushan | KJ688404 | NA | KJ686630 |
| Mallotus penangensis | Müll. Arg. | Euphorbiaceae | Bukit-Timah | KJ594787 | n/a | n/a |
| Mallotus philippensis | (Lam.) M¸ll. Arg. | Euphorbiaceae | Lienhuachih | KJ688410 | NA | KJ686636 |
| Malpighia fucata | Ker Gawl. | Malpighiaceae | Luquillo | HM446820 | HM446707 | HM446952 |
| Malpighia romeroana | Cuatrec. | Malpighiaceae | BCI | GQ981793 | GQ982038 | GQ982279 |
| Malus baccata | (L.) Borkh. | Rosaceae | Changbaishan | CANGB026-14 | n/a | n/a |
| Malus leiocalyca | S.Z. Huang | Rosaceae | Gutianshan | HQ427202 | HQ427351 | n/a |
| Malus pumila | Mill. | Rosaceae | Wabikon Lake | KJ593530 | KJ593022 | n/a |
| Mangifera gracilipes | Hook.f. | Anacardiaceae | Bukit-Timah | KJ594789 | KJ708995 | BTSIN072-13 |
| Mangifera indica | L. | Anacardiaceae | Luquillo | HM446821 | n/a | HM446953 |
| Manglietia yuyuanensis | Y.W. Law | Magnoliaceae | Gutianshan | HQ427274.1 | n/a | n/a |
| Manilkara bidentata | (A.DC.)A.Chev. | Sapotaceae | Luquillo | HM446822 | HM446708 | HM446954 |
| Maquira guianensis | Aubl. | Moraceae | BCI | GQ981794 | GQ982039 | GQ982280 |
| Maranthes corymbosa | Blume | Chrysobalanaceae | Bukit-Timah | KJ594791 | KJ708996 | BTSIN018-13 |
| Margaritaria nobilis | L. f. | Phyllanthaceae | BCI | HM446823 | HM446709 | HM446955 |
| Marila laxiflora | Rusby | Calophyllaceae | BCI | GQ981796 | n/a | GQ982282 |
| Mastixia trichotoma | Blume | Cornaceae | Bukit-Timah | BTSIN578-13 | BTSIN578-13 | BTSIN578-13 |
| Matayba domingensis | (DC.) Radlk. | Sapindaceae | Luquillo | HM446824 | HM446710 | HM446956 |
| Matthaea sancta | Blume | Monimiaceae | Bukit-Timah | KJ594794 | KJ708998 | BTSIN442-13 |
| Maytenus elongata | Britton | Celastraceae | Luquillo | HM446825 | HM446711 | HM446957 |
| Maytenus schippii | Lundell | Celastraceae | BCI | GQ981797 | GQ982041 | GQ982283 |
| Meiogyne virgata | (Blume) Miq. | Annonaceae | Bukit-Timah | KJ594795 | KJ708999 | n/a |
| Melastoma candidum | D. Don | Melastomataceae | Fushan | KJ688413 | NA | KJ687041 |
| Melastoma sanguineum | Sims | Melastomataceae | Dinghushan | HQ415218 | n/a | HQ415564 |
| Melicope glabra | (Blume) T.G. Hartley | Rutaceae | Bukit-Timah | KJ594799 | KJ709002 | BTSIN431-13 |
| Melicope pteleifolia | (Champ. ex Benth.) T.G. Hartley | Rutaceae | Lienhuachih | KJ688417 | NA | KJ687045 |
| Meliosma flexuosa | Pamp. | Sabiaceae | Gutianshan | HQ427214 | HQ427361 | n/a |
| Meliosma fordii | Hemsl. | Sabiaceae | Dinghushan | HQ415133 | n/a | HQ415483 |
| Meliosma herbertii | Rolfe | Sabiaceae | Luquillo | HM446826 | HM446712 | HM446958 |
| Meliosma lanceolata | Blume | Sabiaceae | Bukit-Timah | KJ594801 | KJ709003 | n/a |
| Meliosma oldhamii | Miq. ex Maxim. | Sabiaceae | Gutianshan | HQ427213 | HQ427360 | n/a |
| Meliosma rhoifolia | Maxim. | Sabiaceae | Lienhuachih | KJ688420 | NA | NA |
| Meliosma rigida | Siebold & Zucc. | Sabiaceae | Dinghushan | HQ415132 | HQ415309 | HQ415482 |
| Meliosma squamulata | Hance | Sabiaceae | Fushan | KJ688423 | NA | KJ687049 |
| Memecylon campanulatum | C.B.Clarke | Melastomataceae | Bukit-Timah | BTSIN586-13 | BTSIN586-13 | BTSIN586-13 |
| Memecylon ligustrifolium | Champ. ex Benth. | Melastomataceae | Dinghushan | HQ415087 | HQ415270 | HQ415438 |
| Memecylon megacarpum | Furtado | Melastomataceae | Bukit-Timah | KJ594802 | n/a | BTSIN151-13 |
| Memecylon minutiflorum | Miq. | Melastomataceae | Bukit-Timah | KJ594803 | n/a | n/a |
| Memecylon nigrescens | Hook. & Arn. | Melastomataceae | Dinghushan | HQ415088 | HQ415271 | HQ415439 |
| Mesechites trifidus | (Jacq.) Müll. Arg | Apocynaceae | BCI | n/a | EF456269.1 | n/a |
| Mezzettia parviflora | Becc. | Annonaceae | Bukit-Timah | KJ594804 | KJ709004 | BTSIN184-13 |
| Michelia compressa | (Maxim.) Sarg. | Magnoliaceae | Fushan | KJ688428 | KJ687727 | KJ687054 |
| Michelia foveolata | Merr. ex Dandy | Magnoliaceae | Dinghushan | HQ415092 | HQ415275 | HQ415443 |
| Michelia maudiae | Dunn | Magnoliaceae | Dinghushan | HQ415093 | HQ415276 | HQ415444 |
| Michelia skinnneriana | Dunn | Magnoliaceae | Gutianshan | HQ427275.1 | n/a | n/a |
| Miconia affinis | DC. | Melastomataceae | BCI | GQ981798 | GQ982042 | GQ982284 |
| Miconia argentea | (Sw.)DC. | Melastomataceae | BCI | GQ981799 | GQ982043 | GQ982285 |
| Miconia dorsiloba | Gleason | Melastomataceae | BCI | GQ981800 | n/a | GQ982286 |
| Miconia elata | (Sw.)DC. | Melastomataceae | BCI | GQ981801 | n/a | GQ982287 |
| Miconia hondurensis | Donn.Sm. | Melastomataceae | BCI | GQ981802 | n/a | GQ982288 |
| Miconia impetiolaris | (Sw.) D. Don ex DC. | Melastomataceae | BCI | HM446827 | n/a | HM446959 |
| Miconia laevigata | (L.) D. Don | Melastomataceae | Luquillo | HM446828 | n/a | HM446960 |
| Miconia mirabilis | (Aubl.) L.O. Williams | Melastomataceae | Luquillo | HM446829 | n/a | HM446961 |
| Miconia nervosa | (Sm.)Triana | Melastomataceae | BCI | GQ981804 | n/a | GQ982290 |
| Miconia prasina | (Sw.)DC. | Melastomataceae | Luquillo | HM446830 | n/a | HM446962 |
| Miconia racemosa | (Aubl.) DC. | Melastomataceae | Luquillo | HM446831 | n/a | HM446963 |
| Miconia tetrandra | (Sw.) D. Don ex G. Don | Melastomataceae | Luquillo | HM446832 | n/a | HM446964 |
| Microcos blattaefolia | (Corner) Rao | Malvaceae | Bukit-Timah | KJ594807 | KJ709007 | BTSIN445-13 |
| Microcos paniculata | L. | Malvaceae | Dinghushan | HQ415136 | HQ415312 | HQ415486 |
| Microdesmis caseariifolia | Planch. ex Hook. f. | Pandaceae | Dinghushan | HQ415208 | HQ415373 | HQ415555 |
| Micropholis garciniifolia | Pierre | Sapotaceae | Luquillo | HM446833 | HM446713 | HM446965 |
| Micropholis guyanensis | (A.DC.)Pierre | Sapotaceae | Luquillo | HM446834 | HM446714 | HM446966 |
| Mischocarpus pentapetalus | (Roxb.) Radlk. | Sapindaceae | Dinghushan | HQ415054 | HQ415242 | HQ415406 |
| Monocarpia marginalis | (Scheff.) J. Sinclair | Annonaceae | Bukit-Timah | BTSIN327-13 | BTSIN327-13 | BTSIN327-13 |
| Morella cerifera | (L.) Small | Myricaceae | BCI | AF119179.1 | n/a | EF590715.1 |
| Morinda seibertii | (Standl.) Steyerm. | Rubiaceae | BCI | GQ981805 | n/a | GQ982291 |
| Morus alba | L. | Moraceae | SERC | JN407320.1 | n/a | GQ435324.1 |
| Morus rubra | L. | Moraceae | SERC | U06812.1 | n/a | n/a |
| Mosannona garwoodii | Chatrou&Welzenis | Annonaceae | BCI | GQ981806 | GQ982045 | GQ982292 |
| Mouriri myrtilloides | (Sw.)Poir. | Melastomataceae | BCI | GQ981807 | n/a | GQ982293 |
| Myrcia deflexa | (Poir.) DC. | Myrtaceae | Luquillo | HM446835 | HM446715 | HM446967 |
| Myrcia fallax | (Rich.) DC. | Myrtaceae | Luquillo | HM446836 | HM446716 | HM446968 |
| Myrcia gatunensis | Standl. | Myrtaceae | BCI | GQ981808 | GQ982046 | GQ982294 |
| Myrcia leptoclada | DC. | Myrtaceae | Luquillo | HM446837 | HM446717 | HM446969 |
| Myrcia splendens | (Sw.)DC. | Myrtaceae | Luquillo | HM446838 | HM446718 | HM446970 |
| Myrica esculenta | Buch.-Ham. ex D. Don | Myricaceae | Bukit-Timah | KJ594810 | n/a | n/a |
| Myrica rubra | (Lour.) Siebold & Zucc. | Myricaceae | Fushan | KJ688436 | NA | NA |
| Myristica cinnamomea | King | Myristicaceae | Bukit-Timah | KJ594811 | KJ709009 | BTSIN286-13 |
| Myristica maxima | Warb. | Myristicaceae | Bukit-Timah | BTSIN095-13 | BTSIN095-13 | BTSIN095-13 |
| Myrospermum frutescens | Jacq. | Fabaceae | BCI | GQ981809 | GQ982047 | GQ982295 |
| Myrsine coriacea | (Sw.)R.Br.exRoem.&Schult. | Myrsinaceae | Luquillo | HM446839 | n/a | n/a |
| Myrsine seguinii | H.Lév. | Primulaceae | Dinghushan | HQ415125.1 | HQ415303.1 | HQ415475.1 |
| Nauclea officinalis | (Pierre ex Pit.) Merr. & Chun | Rubiaceae | Dinghushan | HQ415201 | n/a | n/a |
| Nectandra antillana | Meisn. | Lauraceae | Luquillo | GQ981812 | n/a | HM446971 |
| Nectandra cissiflora | Nees | Lauraceae | BCI | GQ981810 | GQ982048 | GQ982296 |
| Nectandra fuzzy | none | Lauraceae | BCI | GQ982049 | GQ981811 | GQ982297 |
| Nectandra hihua | (Ruiz & Pav.) Rohwer | Lauraceae | BCI | JQ592333.1 | JQ588098.1 | HM446971.1 |
| Nectandra lineata | (Kunth)Rohwer | Lauraceae | BCI | GQ982050 | GQ981812 | GQ982298 |
| Nectandra umbrosa | (Kunth)Mez | Lauraceae | BCI | GQ981813 | GQ982051 | GQ982299 |
| Neea amplifolia | Donn.Sm. | Nyctaginaceae | BCI | GQ981814 | GQ982052 | GQ982300 |
| Neesia malayana | Bakh. | Malvaceae | Bukit-Timah | KJ594814 | KJ709011 | BTSIN021-13 |
| Neolitsea aciculata var. variabillima | J.C.Liao | Lauraceae | Fushan | KJ688441 | NA | KJ687063 |
| Neolitsea aurata | (Hayata) Koidz. | Lauraceae | Dinghushan | HQ415213 | HQ415378 | HQ415560 |
| Neolitsea cambodiana | Lecomte | Lauraceae | Dinghushan | HQ415211.1 | HQ415376.1 | HQ415558.1 |
| Neolitsea chui | Merr. | Lauraceae | Dinghushan | HQ415210 | HQ415375 | HQ415557 |
| Neolitsea konishii | (Hayata) Kaneh. & Sasaki | Lauraceae | Fushan | KJ688446 | KJ687737 | NA |
| Neolitsea umbrosa | (Nees) Gamble | Lauraceae | Dinghushan | HQ415212 | HQ415377 | HQ415559 |
| Neolitsea zeylanica | (Nees & T. Nees) Merr. | Lauraceae | Bukit-Timah | KJ594816 | KJ709012 | BTSIN102-13 |
| Neoscortechinia forbesii | (Hook. f.) S. Moore | Euphorbiaceae | Bukit-Timah | KJ594815 | n/a | n/a |
| Neoscortechinia kingii | (Hook. f.) Pax & K. Hoffm. | Euphorbiaceae | Bukit-Timah | n/a | KJ709013 | BTSIN649-13 |
| Nephelium chryseum | Blume | Sapindaceae | Dinghushan | HQ415181 | HQ415352 | HQ415530 |
| Nephelium costatum | Hiern | Sapindaceae | Bukit-Timah | KJ594817 | KJ709014 | BTSIN465-13 |
| Nephelium lappaceum | L. | Sapindaceae | Bukit-Timah | KJ594819 | KJ709016 | BTSIN471-13 |
| Nephelium ramboutan ake | (Labill.) Leenh. | Sapindaceae | Bukit-Timah | KJ594821 | KJ709019 | BTSIN074-13 |
| Nothaphoebe umbelliflora | (Blume) Blume | Lauraceae | Bukit-Timah | KJ594822 | KJ709020 | BTSIN047-13 |
| Nyssa sinensis | Oliv. | Cornaceae | Gutianshan | HQ427178 | HQ427326 | n/a |
| Nyssa sylvatica | Marshall | Cornaceae | SERC | AF119178.1 | n/a | n/a |
| Ochanostachys amentacea | Mast. | Olacaceae | Bukit-Timah | KJ594824 | KJ709022 | n/a |
| Ochroma pyramidale | (Cav.exLam.)Urb. | Bombacaceae | Luquillo | HM446840 | HM446719 | n/a |
| Ocotea cernua | (Nees)Mez | Lauraceae | BCI | GQ981816 | n/a | GQ982302 |
| Ocotea floribunda | (Sw.) Mez | Lauraceae | Luquillo | HM446841 | HM446720 | HM446972 |
| Ocotea leucoxylon | (Sw.)Laness. | Lauraceae | Luquillo | HM446842 | HM446721 | HM446973 |
| Ocotea moschata | (Meisn.) Mez | Lauraceae | Luquillo | HM446843 | HM446722 | HM446974 |
| Ocotea oblonga | (Meisn.)Mez | Lauraceae | BCI | GQ981817 | GQ982053 | GQ982303 |
| Ocotea puberula | (Rich.)Nees | Lauraceae | BCI | GQ981818 | GQ982054 | GQ982304 |
| Ocotea sintenisii | (Mez) A. H. Liogier | Lauraceae | Luquillo | HM446844 | HM446723 | HM446975 |
| Ocotea spathulata | Mez | Lauraceae | Luquillo | HM446845 | HM446724 | HM446976 |
| Ocotea whitei | Woodson | Lauraceae | BCI | GQ981818 | n/a | GQ982305 |
| Oenocarpus mapora | H.Karst. | Arecaceae | BCI | GQ981819 | n/a | GQ982306 |
| Oncosperma horridum | (Griff.) Scheff. | Arecaceae | Bukit-Timah | KJ594825 | KJ709024 | BTSIN486-13 |
| Oreocnide pedunculata | (Shirai) Masam. | Urticaceae | Fushan | KJ688452 | NA | KJ687073 |
| Ormosia amazonica | Ducke | Fabaceae | BCI | GQ981820 | n/a | GQ982307 |
| Ormosia coccinea | (Aubl.)Jacks. | Fabaceae | BCI | GQ981821 | GQ982055 | GQ982308 |
| Ormosia fordiana | Oliv. | Fabaceae:Papil. | Dinghushan | HQ415096 | HQ415278 | HQ415447 |
| Ormosia formosana | Kaneh. | Fabaceae | Lienhuachih | KJ688458 | KJ687743 | KJ687078 |
| Ormosia glaberrima | Y.C. Wu | Fabaceae:Papil. | Dinghushan | HQ415097 | HQ415279 | HQ415448 |
| Ormosia krugii | Urb. | Fabaceae:Papil. | Luquillo | HM446846 | HM446725 | HM446977 |
| Ormosia macrocalyx | Ducke | Fabaceae | BCI | GQ981822 | GQ982056 | GQ982309 |
| Ormosia semicastrata | Hance | Fabaceae:Papil. | Dinghushan | HQ415098 | HQ415280 | HQ415449 |
| Orophea creaghii | (Ridl.) Leonard’a & P.J.A. Kessler | Annonaceae | Bukit-Timah | KJ594826 | KJ709025 | BTSIN443-13 |
| Osmanthus cooperi | Hemsl. | Oleaceae | Gutianshan | HQ427188 | HQ427336 | n/a |
| Osmanthus matsumuranus | Hayata | Oleaceae | Fushan | KJ688459 | KJ687744 | KJ687079 |
| Osmelia philippina | Fern.-Vill. | Salicaceae | Bukit-Timah | KJ594827 | KJ709026 | BTSIN298-13 |
| Ostrya virginiana | (Mill.) K. Koch | Betulaceae | Wabikon Lake | KJ593568 | KJ593034 | n/a |
| Ouratea lucens | (Kunth)Engl. | Ochnaceae | BCI | GQ981823 | n/a | n/a |
| Oxandra laurifolia | (Sw.) A. Rich. | Annonaceae | Luquillo | HM446847 | HM446726 | HM446978 |
| Pachira quinata | (Jacq.)W.S.Alverson | Malvaceae | BCI | GQ981824 | GQ982057 | GQ982310 |
| Pachira sessilis | Benth. | Malvaceae | BCI | n/a | n/a | n/a |
| Padus avium | Mill. | Rosaceae | Changbaishan | CANGB027-14 | n/a | n/a |
| Palaquium impressinervium | Ng | Sapotaceae | Bukit-Timah | KJ594829 | n/a | n/a |
| Palaquium microphyllum | King & Gamble | Sapotaceae | Bukit-Timah | n/a | KJ709027 | n/a |
| Palaquium obovatum | (Griff.) Engl. | Sapotaceae | Bukit-Timah | KJ594830 | KJ709028 | BTSIN351-13 |
| Palaquium oxleyanum | Burck | Sapotaceae | Bukit-Timah | KJ594831 | n/a | n/a |
| Palicourea guianensis | Aubl. | Rubiaceae | BCI | GQ981825 | GQ982058 | GQ982311 |
| Palicourea riparia | Benth. | Rubiaceae | Luquillo | HM446848 | HM446727 | HM446979 |
| Pandanus sp. | na | Pandaceae | Bukit-Timah | KJ594832 | KJ709029 | n/a |
| Parartocarpus bracteatus | (King) Becc. | Moraceae | Bukit-Timah | KJ594833 | n/a | BTSIN094-13 |
| Parinari argentasericea | Kosterm. | Chrysobalanaceae | Bukit-Timah | n/a | KJ709030 | n/a |
| Parinari oblongifolia | Hook. f. | Chrysobalanaceae | Bukit-Timah | n/a | KJ709031 | BTSIN672-13 |
| Parishia maingayi | ex Hook. f. | Anacardiaceae | Bukit-Timah | KJ594834 | KJ709032 | n/a |
| Parishia paucijuga | Engl. | Anacardiaceae | Bukit-Timah | KJ594835 | KJ709033 | BTSIN105-13 |
| Parkia speciosa | Hassk. | Fabaceae | Bukit-Timah | KJ594837 | KJ709035 | BTSIN475-13 |
| Pasania hancei var. ternaticupula | (Hayata)J.C.Liao | Fagaceae | Fushan | KJ688466 | KJ687751 | KJ687086 |
| Pasania harlandii | (Hance ex Walp.) Oerst. | Fagaceae | Fushan | KJ688474 | KJ687756 | KJ687093 |
| Pasania konishii | (Hayata) Schottky | Fagaceae | Lienhuachih | KJ688484 | KJ687760 | KJ687100 |
| Pasania nantoensis | (Hayata) Schottky | Fagaceae | Lienhuachih | KJ688485 | KJ687761 | KJ687101 |
| Pasania synbalanos | (Hance) Schottky | Fagaceae | Lienhuachih | KJ688492 | NA | NA |
| Paulownia tomentosa | (Thunb.) Steud. | Paulowniaceae | SCBI | L36447.1 | AF051997.1 | n/a |
| Pavetta hongkongensis | Bremek. | Rubiaceae | Dinghushan | HQ415062 | HQ415250 | HQ415414 |
| Payena lucida | A. DC. | Sapotaceae | Bukit-Timah | KJ594838 | KJ709037 | BTSIN372-13 |
| Payena maingayi | C.B. Clarke | Sapotaceae | Bukit-Timah | KJ594840 | KJ709038 | BTSIN052-13 |
| Pellacalyx saccardianus | Scort. | Rhizophoraceae | Bukit-Timah | KJ594842 | KJ709040 | BTSIN303-13 |
| Pentace triptera | Mast. | Malvaceae | Bukit-Timah | KJ594844 | KJ709042 | BTSIN210-13 |
| Pentagonia macrophylla | Benth. | Rubiaceae | BCI | GQ981826 | GQ982059 | GQ982312 |
| Pentaphylax euryoides | Gardner & Champ. | Pentaphylacaceae | Dinghushan | HQ415203 | HQ415369 | HQ415550 |
| Perebea xanthochyma | H.Karst. | Moraceae | BCI | GQ981827 | GQ982060 | GQ982313 |
| Perrottetia arisanensis | Hayata | Dipentodontaceae | Fushan | KJ688496 | KJ687769 | KJ687109 |
| Pertusadina hainanensis | (F.C. How) Ridsdale | Rubiaceae | Gutianshan | HQ427197 | HQ427346 | n/a |
| Phaeanthus ophthalmicus | (Roxb. ex G.Don) J.Sinclair | Annonaceae | Bukit-Timah | KJ594845 | KJ709043 | BTSIN083-13 |
| Phellodendron amurense | Rupr. | Rutaceae | Changbaishan | CANGB028-14 | n/a | n/a |
| Philadelphus schrenkii | Rupr. | Hydrangeaceae | Changbaishan | CANGB029-14 | n/a | n/a |
| Photinia beauverdiana | C.K. Schneid. | Rosaceae | Gutianshan | HQ427204 | HQ427353 | n/a |
| Photinia glabra | (Thunb.) Franch. & Sav. | Rosaceae | Gutianshan | HQ427205 | HQ427354 | n/a |
| Photinia parvifolia | (E. Pritz.) C.K. Schneid. | Rosaceae | Gutianshan | HQ427206 | HQ427355 | n/a |
| Photinia prunifolia | (Hook. & Arn.) Lindl. | Rosaceae | Dinghushan | HQ415183 | n/a | HQ415532 |
| Photinia serrulata | Lindl. | Rosaceae | Gutianshan | HQ427207.1 | n/a | n/a |
| Phyllanthus glaucus | Wall. ex M¸ll. Arg. | Euphorbiaceae | Gutianshan | HQ427130 | n/a | n/a |
| Picea abies | (L.) H. Karst. | Pinaceae | Wytham | NC_021456 | NC_021456 | NC_021456 |
| Picea glauca | (Moench) Voss | Pinaceae | Wabikon Lake | KJ593601 | n/a | n/a |
| Picea jezoensis | (Siebold & Zucc.) CarriËre | Pinaceae | Changbaishan | CANGB030-14 | n/a | n/a |
| Picea koraiensis | Nakai | Pinaceae | Changbaishan | CANGB031-14 | n/a | n/a |
| Picea mariana | (Mill.) Britton, Sterns & Poggenb. | Pinaceae | Wabikon Lake | KJ593605 | n/a | n/a |
| Picramnia latifolia | Tul. | Picramniaceae | BCI | GQ981828 | n/a | GQ982314 |
| Picrasma quassioides | (D. Don) Benn. | Simaroubaceae | Gutianshan | HQ427179 | HQ427327 | n/a |
| Pieris formosa | (Wall.) D. Don | Ericaceae | Gutianshan | HQ427148 | HQ427301 | n/a |
| Pieris japonica | (Thunb.) D. Don ex G. Don | Ericaceae | Gutianshan | HQ427151 | HQ427303 | n/a |
| Pimelodendron griffithianum | (J. Mueller-Arg. in Alph. de Candolle) Benth. ex Hook. f. | Euphorbiaceae | Bukit-Timah | KJ594847 | n/a | n/a |
| Pinanga sp | n/a | Arecaceae | Bukit-Timah | KJ594849 | KJ709045 | n/a |
| Pinus koraiensis | Siebold & Zucc. | Pinaceae | Changbaishan | CANGB032-14 | n/a | n/a |
| Pinus lambertiana | Douglas | Pinaceae | Yosemite | NC_011156 | NC_011156 | NC_011156 |
| Pinus massoniana | D. Don | Pinaceae | Dinghushan | HQ427243 | HQ427386 | n/a |
| Pinus morrisonicola | Hayata | Pinaceae | Lienhuachih | KJ688497 | NA | NA |
| Pinus ponderosa | P. Lawson & C. Lawson | Pinaceae | Yosemite | AY497234.1 | AY497270.1 | n/a |
| Pinus pungens | Lamb. | Pinaceae | SCBI | KC156900.1 | KC157032.1 | KC157397.1 |
| Pinus strobus | L. | Pinaceae | Wabikon Lake | KJ593613 | n/a | n/a |
| Pinus sylvestris var sylvestriformis | (Taken.) W.C.Cheng & C.D.Chu | Pinaceae | Changbaishan | CANGB033-14 | n/a | n/a |
| Pinus taeda | L. | Pinaceae | SERC | AF119177.1 | n/a | n/a |
| Pinus virginiana | Mill. | Pinaceae | SERC | AB063379.1 | n/a | n/a |
| Piper aduncum | L. | Piperaceae | Luquillo | HM446849 | HM446728 | HM446980 |
| Piper aequale | Vahl | Piperaceae | BCI | GQ981829 | n/a | GQ982315 |
| Piper arboreum | Aubl. | Piperaceae | BCI | n/a | GQ981830 | GQ982316 |
| Piper blattarum | Spreng. | Piperaceae | Luquillo | HM446850 | HM446729 | HM446981 |
| Piper carrilloanum | C. DC. | Piperaceae | BCI | GQ981831 | GQ982061 | GQ982320 |
| Piper colonense | C.DC. | Piperaceae | BCI | GQ981832 | n/a | n/a |
| Piper cordulatum | C.DC. | Piperaceae | BCI | GQ981833 | GQ982062 | GQ982317 |
| Piper glabrescens | (Miq.) C. DC. | Piperaceae | Luquillo | HM446851 | HM446730 | HM446982 |
| Piper hispidum | Sw. | Piperaceae | Luquillo | HM446852 | n/a | HM446983 |
| Piper multiplinervium | C. DC. | Piperaceae | BCI | JQ593229.1 | JQ588651.1 | n/a |
| Piper perlasense | Yunck. | Piperaceae | BCI | GQ981834 | GQ982063 | GQ982318 |
| Piper reticulatum | L. | Piperaceae | BCI | GQ981835 | n/a | GQ982319 |
| Piper umbellatum | L. | Piperaceae | Luquillo | HM446853 | n/a | HM446984 |
| Pisonia subcordata | Sw. | Nyctaginaceae | Luquillo | HM446854 | HM446731 | HM446985 |
| Pithecellobium clypearia | (Jack) Benth. | Fabaceae:Mimos. | Dinghushan | HQ415100.1 | HQ415281.1 | HQ415451.1 |
| Pithecellobium hymenaeifolium | (Humb. & Bonpl. ex Willd.) Benth. | Fabaceae | BCI | KJ082505 | KJ012723 | KJ426883 |
| Pittosporum glabratum | Lindl. | Pittosporaceae | Dinghushan | HQ415091 | HQ415274 | HQ415442 |
| Pittosporum illicioides | Makino | Pittosporaceae | Gutianshan | HQ427157 | HQ427307 | n/a |
| Platanus occidentalis | L. | Platanaceae | SERC | AF081073.1 | AF543747.1 | HE661195.1 |
| Platycarya strobilacea | Siebold & Zucc. | Juglandaceae | Gutianshan | HQ427158 | HQ427308 | n/a |
| Platymiscium pinnatum | (Jacq.)Dugand | Fabaceae | BCI | n/a | GQ982064 | GQ982321 |
| Platypodium elegans | Vogel | Fabaceae | BCI | GQ981836 | GQ982065 | GQ982322 |
| Pleodendron macranthum | (Baill.) Tiegh. | Canellaceae | Luquillo | HM446855 | HM446732 | HM446986 |
| Pluchea symphytifolia | (Mill.) Gillis | Asteraceae | Luquillo | JQ590700.1 | n/a | n/a |
| Podocarpus nakaii | Hayata | Podocarpaceae | Lienhuachih | KJ688679 | NA | NA |
| Polyalthia glauca | (Hassk.) Boerl. | Annonaceae | Bukit-Timah | KJ594850 | n/a | n/a |
| Polyalthia hypoleuca | Hook. f. & Thomson | Annonaceae | Bukit-Timah | KJ594851 | KJ709046 | BTSIN355-13 |
| Polyalthia macropoda | King | Annonaceae | Bukit-Timah | KJ594852 | KJ709047 | n/a |
| Polyalthia rumphii | (Blume ex Hensch.) Merr. | Annonaceae | Bukit-Timah | KJ594854 | KJ709049 | BTSIN103-13 |
| Polyosma kingiana | Schltr. | Escalloniaceae | Bukit-Timah | KJ594855 | KJ709050 | BTSIN462-13 |
| Popowia pisocarpa | (Blume) Endl. | Annonaceae | Bukit-Timah | KJ594857 | KJ709052 | BTSIN453-13 |
| Popowia tomentosa | Maingay ex Hook.f. & Thomson | Annonaceae | Bukit-Timah | KJ594858 | KJ709053 | BTSIN380-13 |
| Populus balsamifera | L. | Salicaceae | Wabikon Lake | KJ593626 | KJ593064 | n/a |
| Populus davidiana | Dode | Salicaceae | Changbaishan | CANGB034-14 | n/a | n/a |
| Populus koreana | Rehder | Salicaceae | Changbaishan | CANGB035-14 | n/a | n/a |
| Populus tremuloides | Michx. | Salicaceae | Wabikon Lake | KJ593631 | KJ593068 | n/a |
| Populus ussuriensis | Kom. | Salicaceae | Changbaishan | CANGB036-14 | n/a | n/a |
| Porterandia anisophylla | (Jack ex Roxb.) Ridl. | Rubiaceae | Bukit-Timah | KJ594859 | KJ709054 | BTSIN290-13 |
| Posoqueria latifolia | (Rudge)Schult. | Rubiaceae | BCI | GQ981837 | GQ982066 | GQ982323 |
| Poulsenia armata | (Miq.)Standl. | Moraceae | BCI | GQ981838 | n/a | GQ982324 |
| Pourouma bicolor | Mart. | Urticaceae | BCI | GQ981839 | GQ982067 | GQ982325 |
| Pourthiaea beauverdiana var. notabilis | (C.K.Schneid.)Hatus. | Rosaceae | Fushan | KJ688498 | KJ687770 | KJ686637 |
| Pouteria fossicola | Cronquist | Sapotaceae | BCI | GQ981840 | GQ982068 | GQ982326 |
| Pouteria malaccensis | (C.B. Clarke) Baehni | Sapotaceae | Bukit-Timah | KJ594861 | KJ709056 | BTSIN061-13 |
| Pouteria reticulata | (Engl.)Eyma | Sapotaceae | BCI | n/a | GQ981841 | GQ982327 |
| Pouteria stipitata | Cronquist | Sapotaceae | BCI | GQ981842 | GQ982069 | GQ982328 |
| Premna microphylla | Turcz. | Lamiaceae | Gutianshan | HQ427183 | HQ427331 | n/a |
| Prestoea montana | (Graham) G. Nicholson | Arecaceae | Luquillo | HM446857.1 | HM446733.1 | HM446987.1 |
| Prioria copaifera | Griseb. | Fabaceae | BCI | n/a | n/a | GQ982329 |
| Prismatomeris glabra | (Korth.) Valeton | Rubiaceae | Bukit-Timah | KJ594862 | KJ709057 | BTSIN275-13 |
| Protium confusum | (Rose)Pittier | Burseraceae | BCI | GQ981843 | GQ982070 | GQ982330 |
| Protium costaricense | (Rose)Engl. | Burseraceae | BCI | GQ981844 | GQ982071 | GQ982331 |
| Protium panamense | (Rose)I.M.Johnst. | Burseraceae | BCI | GQ981845 | n/a | GQ982332 |
| Protium tenuifolium | (Engl.)Engl. | Burseraceae | BCI | GQ981846 | n/a | GQ982333 |
| Prunus arborea | (Blume) Kalkman | Rosaceae | Bukit-Timah | KJ594863 | KJ709058 | BTSIN280-13 |
| Prunus avium | (L.) L. | Rosaceae | Wytham | HQ235394.1 | FJ899109.1 | n/a |
| Prunus emarginata | (Douglas) Eaton | Rosaceae | Yosemite | U06820.1 | n/a | n/a |
| Prunus persica | (L.) Batsch | Rosaceae | SCBI | NC_014697 | NC_014697 | NC_014697 |
| Prunus phaeosticta | (Hance) Maxim. | Rosaceae | Fushan | KJ688505 | KJ687776 | KJ687113 |
| Prunus polystachya | Kalkman | Rosaceae | Bukit-Timah | KJ594865 | KJ709060 | BTSIN251-13 |
| Prunus schneideriana | Koehne | Rosaceae | Gutianshan | HQ427209 | HQ427356 | n/a |
| Prunus serotina | Ehrh. | Rosaceae | Wabikon Lake | KJ593640 | KJ593077 | n/a |
| Prunus serrulata | Lindl. | Rosaceae | Changbaishan | CANGB037-14 | n/a | n/a |
| Prunus spinosa | L. | Rosaceae | Wytham | n/a | HQ235276.1 | FR865110.1 |
| Prunus spinulosa | Siebold & Zucc. | Rosaceae | Gutianshan | HQ427210 | HQ427357 | n/a |
| Prunus virginiana | L. | Rosaceae | Wabikon Lake | KJ593643 | KJ593079 | n/a |
| Pseudobombax septenatum | (Jacq.)Dugand | Malvaceae | BCI | GQ981847 | GQ982072 | GQ982334 |
| Pseudolmedia spuria | (Sw.)Griseb. | Moraceae | Luquillo | HM446858 | HM446734 | HM446988 |
| Pseudosamanea guachapele | (Kunth) Harms | Fabaceae | BCI | JQ591565.1 | n/a | AF524983.1 |
| Pseudotsuga menziesii | (Mirb.) Franco | Pinaceae | Wind_River | NC_016064 | NC_016064 | NC_016064 |
| Psidium friedrichsthalianum | (O.Berg)Nied. | Myrtaceae | BCI | GQ981848 | GQ982073 | GQ982335 |
| Psychotria acuminata | Benth. | Rubiaceae | BCI | GQ981849 | GQ982074 | GQ982336 |
| Psychotria asiatica | L. | Rubiaceae | Dinghushan | HQ415119 | HQ415297 | HQ415469 |
| Psychotria berteroana | DC. | Rubiaceae | Luquillo | HM446859 | HM446735 | HM446989 |
| Psychotria brachiata | Sw. | Rubiaceae | Luquillo | HM446860 | HM446736 | HM446990 |
| Psychotria chagrensis | Standl. | Rubiaceae | BCI | GQ981850 | GQ982075 | GQ982337 |
| Psychotria cyanococca | Dombrain | Rubiaceae | BCI | GQ981851 | n/a | GQ982338 |
| Psychotria deflexa | DC. | Rubiaceae | Luquillo | HM446861 | HM446737 | HM446991 |
| Psychotria graciliflora | Benth. | Rubiaceae | BCI | GQ981853 | GQ982077 | GQ982340 |
| Psychotria grandis | Sw. | Rubiaceae | Luquillo | HM446862 | HM446738 | HM446992 |
| Psychotria hoffmannseggiana | (Schult.)MŸll.Arg. | Rubiaceae | BCI | GQ981853 | n/a | n/a |
| Psychotria horizontalis | Sw. | Rubiaceae | BCI | GQ981855 | n/a | GQ982342 |
| Psychotria limonensis | K.Krause | Rubiaceae | BCI | GQ981856 | GQ982079 | GQ982343 |
| Psychotria marginata | Sw. | Rubiaceae | BCI | GQ981857 | GQ982080 | GQ982344 |
| Psychotria psychotriifolia | (Seem.)Standl. | Rubiaceae | BCI | GQ981858 | n/a | GQ982345 |
| Psychotria racemosa | Rich. | Rubiaceae | BCI | GQ981859 | n/a | GQ982346 |
| Psychotria rubra | (Lour.) Poir. | Rubiaceae | Lienhuachih | KJ688513 | NA | KJ687121 |
| Psychotria tenuifolia | Sw. | Rubiaceae | BCI | GQ981860 | GQ982081 | GQ982347 |
| Psydrax dicoccos | Gaertn. | Rubiaceae | Dinghushan | HQ415205.1 | HQ415371.1 | HQ415571.1 |
| Pternandra coerulescens | Jack | Melastomataceae | Bukit-Timah | n/a | KJ709061 | BTSIN167-13 |
| Pterocarpus belizensis | Standl. | Fabaceae | BCI | HM446863.1 | HM446739.1 | HM446993.1 |
| Pterocarpus officinalis | Jacq. | Fabaceae:Papil. | Luquillo | HM446863 | HM446739 | HM446993 |
| Pterocarpus rohrii | Vahl | Fabaceae | BCI | GQ981862* | n/a | n/a |
| Pterospermum heterophyllum | Hance | Malvaceae | Dinghushan | HQ415057 | HQ415245 | HQ415409 |
| Pterospermum lanceifolium | Roxb. | Malvaceae | Dinghushan | HQ415058 | HQ415246 | HQ415410 |
| Ptychopyxis caput-medusae | (Hook. f.) Ridl. | Euphorbiaceae | Bukit-Timah | KJ594867 | KJ709062 | BTSIN215-13 |
| Pygeum topengii | Merr. | Rosaceae | Dinghushan | HQ415196 | n/a | HQ415544 |
| Pyrenaria shinkoensis | (Hayata) H. Keng | Theaceae | Fushan | KJ688514 | KJ687781 | KJ687122 |
| Quararibea asterolepis | Pittier | Malvaceae | BCI | n/a | n/a | GQ982350 |
| Quararibea turbinata | (Sw.) Poir. | Malvaceae | Luquillo | HM446864 | HM446740 | HM446994 |
| Quassia amara | L. | Simaroubaceae | BCI | GQ981863 | n/a | GQ982351 |
| Quercus alba | L. | Fagaceae | SCBI | LM652853.1 | LM652870.1 | LM652974.1 |
| Quercus argentata | Martin-Donos & Timb.-Lagr. | Fagaceae | Bukit-Timah | KJ594869 | KJ709063 | BTSIN341-13 |
| Quercus marilandica | (L.) MŸnchh. | Fagaceae | SCBI | LM652853.1 | n/a | n/a |
| Quercus michauxii | Nutt. | Fagaceae | SCBI | LM652853.1 | n/a | n/a |
| Quercus mongolica | Fisch. ex Ledeb. | Fagaceae | Changbaishan | CANGB038-14 | n/a | n/a |
| Quercus muehlenbergii | Engelm. | Fagaceae | SCBI | KF683161.1 | n/a | n/a |
| Quercus phillyraeoides | A. Gray | Fagaceae | Gutianshan | HQ427176 | HQ427324 | n/a |
| Quercus robur | L. | Fagaceae | Wytham | KF683161.1 | n/a | n/a |
| Quercus rubra | L. | Fagaceae | Wabikon Lake | KJ593650 | KJ593082 | n/a |
| Quercus serrata | Thunb. | Fagaceae | Gutianshan | HQ427171.1 | n/a | n/a |
| Radermachera sinica | (Hance) Hemsl. | Bignoniaceae | Lienhuachih | KJ688522 | KJ687788 | KJ687129 |
| Randia armata | (Sw.)DC. | Rubiaceae | BCI | GQ981864 | GQ982084 | GQ982352 |
| Randia cochinchinensis | (Lour.) Merr. | Rubiaceae | Fushan | KJ688526 | KJ687792 | KJ687133 |
| Raphiolepis indica | n/a | Rosaceae | Gutianshan | HQ415182 | HQ415353 | HQ415531 |
| Rauvolfia littoralis | Rusby | Apocynaceae | BCI | GQ981865 | GQ982085 | n/a |
| Rauvolfia nitida | Jacq. | Apocynaceae | Luquillo | DQ660663 | n/a | n/a |
| Reevesia formosana | Sprague | Malvaceae | Lienhuachih | KJ688530 | NA | NA |
| Reevesia pycnantha | Ling | Malvaceae | Gutianshan | HQ427249 | HQ427392 | n/a |
| Reevesia thyrsoidea | Lindl. | Malvaceae | Dinghushan | HQ415190 | HQ415360 | HQ415539 |
| Rhamnus crenata | Siebold & Zucc. | Rhamnaceae | Gutianshan | HQ427242 | HQ427385 | n/a |
| Rhamnus ussuriensis | J.J. Vassil. | Rhamnaceae | Changbaishan | CANGB039-14 | n/a | n/a |
| Rhaphiolepis indica | (L.) Lindl. ex Ker | Rosaceae | Dinghushan | HQ415182 | HQ415353 | HQ415531 |
| Rhaphiolepis indica var tashiroi | Hayata | Rosaceae | Lienhuachih | KJ688531 | NA | KJ687137 |
| Rheedia_portoricensis | Urb. | Malpighiaceae | Luquillo | AF518377.1 | n/a | HM446995.1 |
| Rhodamnia cinerea | Jack | Myrtaceae | Bukit-Timah | KJ594870 | KJ709064 | BTSIN149-13 |
| Rhododendron henryi | Hance | Ericaceae | Dinghushan | HQ415074 | HQ415258 | HQ415425 |
| Rhododendron latoucheae | Franch. | Ericaceae | Gutianshan | HQ427145 | HQ427298 | n/a |
| Rhododendron leptosanthum | Hayata | Ericaceae | Fushan | NA | NA | KJ687140 |
| Rhododendron mariae | Hance | Ericaceae | Dinghushan | HQ415073 | n/a | n/a |
| Rhododendron mariesii | Hemsl. & E.H. Wilson | Ericaceae | Lienhuachih | KJ688539 | NA | KJ687146 |
| Rhododendron ovatum | (Lindl.) Planch. ex Maxim. | Ericaceae | Gutianshan | HQ427144 | HQ427297 | n/a |
| Rhododendron simsii | Planch. | Ericaceae | Dinghushan | HQ427146 | HQ427299 | HQ415427 |
| Rhodomyrtus tomentosa | (Aiton) Hassk. | Myrtaceae | Dinghushan | HQ415191 | HQ415361 | HQ415540 |
| Rhus hypoleuca | Champ. ex Benth. | Anacardiaceae | Gutianshan | n/a | HQ427342 | n/a |
| Rhus succedanea | L. | Anacardiaceae | Fushan | KJ688544 | KJ687802 | KJ687150 |
| Ribes mandshuricum | (Maxim.) Kom. | Grossulariaceae | Changbaishan | CANGB040-14 | n/a | n/a |
| Rinorea sylvatica | (Seem.)Kuntze | Violaceae | BCI | GQ981866 | GQ982086 | GQ982353 |
| Robinia pseudoacacia | L. | Fabaceae | SCBI | DQ006096.1 | n/a | n/a |
| Rondeletia portoricensis | J.C. Krug & Urb. | Rubiaceae | Luquillo | HM446866 | HM446741 | HM446996 |
| Rosa macrophylla | Lindl. | Rosaceae | Changbaishan | CANGB041-14 | n/a | n/a |
| Rosenbergiodendron formosum | (Jacq.)Fagerl. | Rubiaceae | BCI | GQ981867 | GQ982087 | GQ982354 |
| Roystonea borinquena | O.F. Cook | Arecaceae | Luquillo | HM446867 | HM446742 | HM446997 |
| Rubus chingii | Hu | Rosaceae | Gutianshan | HQ427211 | HQ427358 | n/a |
| Rubus pensilvanicus | Poir. | Rosaceae | SCBI | EU676983.1 | n/a | n/a |
| Rubus phoenicolasius | Maxim. | Rosaceae | SCBI | JN965825.1 | n/a | n/a |
| Rubus_allegheniensis | Porter | Rosaceae | SCBI | EU676983.1 | EU749375.1 | EU750524.1 |
| Ryania speciosa | Vahl | Salicaceae | BCI | HM446868 | n/a | n/a |
| Sambucus nigra | L. | Adoxaceae | Wytham | n/a | FN668836.1 | FN675824.1 |
| Sambucus racemosa | L. | Adoxaceae | Wabikon Lake | KJ593677 | KJ593099 | n/a |
| Sambucus williamsii | Hance | Adoxaceae | Changbaishan | CANGB042-14 | n/a | n/a |
| Sambucus_canadensis | L. | Adoxaceae | SCBI | HQ590258.1 | HQ593429.1 | HQ596833.1 |
| Samyda dodecandra | Jacq. | Salicaceae | Luquillo | HM446868 | HM446743.1 | n/a |
| Sandoricum beccarianum | Baill. | Meliaceae | Bukit-Timah | KJ594872 | KJ709065 | n/a |
| Santiria apiculata | A.W.Benn. | Burseraceae | Bukit-Timah | KJ594873 | KJ709066 | BTSIN230-13 |
| Santiria griffithii | Engl. | Burseraceae | Bukit-Timah | KJ594874 | KJ709067 | BTSIN594-13 |
| Santiria laevigata | Blume | Burseraceae | Bukit-Timah | KJ594876 | KJ709069 | BTSIN382-13 |
| Santiria rubiginosa | Blume | Burseraceae | Bukit-Timah | BTSIN655-13 | n/a | BTSIN655-13 |
| Santiria tomentosa | Blume | Burseraceae | Bukit-Timah | KJ594877 | KJ709070 | BTSIN622-13 |
| Sapindus mukorossi | Gaertn. | Sapindaceae | Lienhuachih | KJ688552 | KJ687809 | KJ687158 |
| Sapium broadleaf | n/a | Euphorbiaceae | BCI | GQ982088 | GQ981868 | GQ982355 |
| Sapium discolor | (Champ. ex Benth.) M¸ll. Arg. | Euphorbiaceae | Dinghushan | HQ415199 | HQ415366 | HQ415547 |
| Sapium glandulosum | (L.)Morong | Euphorbiaceae | BCI | GQ981869 | GQ982089 | GQ982356 |
| Sapium laurocerasus | Desf. | Euphorbiaceae | Luquillo | HM446869 | n/a | HM446999 |
| Saprosma glomerulatum | King & Gamble | Rubiaceae | Bukit-Timah | KJ594878 | KJ709071 | BTSIN273-13 |
| Sarcosperma laurinum | (Benth.) Hook. f. | Sapotaceae | Dinghushan | HQ415158 | HQ415331 | HQ415507 |
| Sarcotheca griffithii | Hallier f. | Oxalidaceae | Bukit-Timah | n/a | KJ709072 | BTSIN603-13 |
| Sassafras albidum | (Nutt.) Nees | Lauraceae | SCBI | GU271217.1 | n/a | EF491223.1 |
| Sassafras tzumu | (Hemsl.) Hemsl. | Lauraceae | Gutianshan | HQ427277 | HQ427419 | n/a |
| Saurauia tristyla | DC. | Actinidiaceae | Dinghushan | n/a | EU310435.1 | n/a |
| Scaphium macropodum | Beumee ex K. Heyne | Malvaceae | Bukit-Timah | KJ594879 | KJ709073 | BTSIN339-13 |
| Schefflera heptaphylla | (L.) Frodin | Araliaceae | Dinghushan | HQ415082.1 | n/a | HQ415433.1 |
| Schefflera morototoni | (Aubl.)Maguire,Steyerm.&Frodin | Araliaceae | Luquillo | HM446870 | HM446744 | HM447000 |
| Schefflera octophylla | (Lour.) Harms | Araliaceae | Fushan | KJ688559 | KJ687814 | KJ687161 |
| Schima superba | Gardner & Champ. | Theaceae | Lienhuachih | KJ688568 | KJ687823 | KJ687170 |
| Schizolobium parahyba | (Vell.)S.F.Blake | Fabaceae | BCI | GQ981870 | GQ982090 | GQ982357 |
| Schoepfia chinensis | Gardner & Champ. | Olacaceae | Dinghushan | HQ415145 | HQ415320 | HQ415495 |
| Schoepfia jasminodora | Siebold & Zucc. | Olacaceae | Dinghushan | HQ415146 | HQ415321 | HQ415496 |
| Scleropyrum wallichianum | (Wight & Arn.) Arn. | Santalaceae | Bukit-Timah | KJ594880 | KJ709074 | BTSIN272-13 |
| Scorodocarpus borneensis | (Baill.) Becc. | Olacaceae | Bukit-Timah | KJ594882 | KJ709075 | BTSIN266-13 |
| Senna dariensis | (Britton&Rose)H.S.Irwin&Barneby | Fabaceae | BCI | GQ981871 | GQ982091 | GQ982358 |
| Shorea assamica | Dyer | Dipterocarpaceae | Bukit-Timah | n/a | KJ709077 | BTSIN581-13 |
| Shorea curtisii | Dyer ex King | Dipterocarpaceae | Bukit-Timah | BTSIN109-13 | n/a | BTSIN109-13 |
| Shorea gibbosa | Brandis | Dipterocarpaceae | Bukit-Timah | KJ594885 | KJ709079 | BTSIN030-13 |
| Shorea gratissima | Dyer | Dipterocarpaceae | Bukit-Timah | KJ594886 | n/a | BTSIN358-13 |
| Shorea ochrophloia | Symington | Dipterocarpaceae | Bukit-Timah | n/a | KJ709081 | BTSIN668-13 |
| Shorea ovalis | Blume | Dipterocarpaceae | Bukit-Timah | n/a | KJ709082 | BTSIN628-13 |
| Shorea pauciflora | King | Dipterocarpaceae | Bukit-Timah | n/a | KJ709083 | BTSIN580-13 |
| Simarouba amara | Aubl. | Simaroubaceae | BCI | HM446871 | n/a | HM447001 |
| Sindora coriacea | (Baker) Prain | Fabaceae | Bukit-Timah | KJ594887 | KJ709084 | BTSIN179-13 |
| Sindora echinocalyx | Prain | Fabaceae | Bukit-Timah | KJ594891 | KJ709088 | BTSIN042-13 |
| Siparuna guianensis | Aubl. | Siparunaceae | BCI | GQ981872 | GQ982092 | GQ982360 |
| Siparuna pauciflora | (Beurl.)A.DC. | Siparunaceae | BCI | GQ981873 | GQ982093 | GQ982361 |
| Sloanea berteroana | Choisy ex DC. | Elaeocarpaceae | Luquillo | HM446872 | HM446745 | HM447002 |
| Sloanea sinensis | (Hance) Hemsl. | Elaeocarpaceae | Dinghushan | HQ427152 | n/a | n/a |
| Sloanea terniflora | (DC.)Standl. | Elaeocarpaceae | BCI | GQ981874 | GQ982094 | GQ982362 |
| Socratea exorrhiza | (Mart.)H.Wendl. | Arecaceae | BCI | GQ981875 | GQ982095 | GQ982363 |
| Solanum asperum | Rich. | Solanaceae | BCI | GQ981876 | GQ982096 | GQ982364 |
| Solanum circinatum | Bohs | Solanaceae | BCI | GQ981877 | GQ982097 | GQ982365 |
| Solanum hayesii | Fernald | Solanaceae | BCI | GQ981878 | GQ982098 | GQ982366 |
| Solanum lepidotum | Dunal | Solanaceae | BCI | GQ981879 | GQ982099 | GQ982367 |
| Solanum rugosum | Dunal | Solanaceae | Luquillo | JQ594175.1 | JQ589269.1 | n/a |
| Solanum torvum | Sw. | Solanaceae | Luquillo | KJ082583 | KJ012783 | KJ426943 |
| Sorbaria sorbifolia | (L.) A. Braun | Rosaceae | Changbaishan | CANGB043-14 | n/a | n/a |
| Sorbus alnifolia | (Siebold & Zucc.) C. Koch | Rosaceae | Changbaishan | CANGB044-14 | n/a | n/a |
| Sorbus folgneri | (C.K. Schneid.) Rehder | Rosaceae | Gutianshan | HQ427212 | HQ427359 | n/a |
| Sorbus pohuashanensis | (Hance) Hedl. | Rosaceae | Changbaishan | CANGB045-14 | n/a | n/a |
| Sorocea affinis | Hemsl. | Moraceae | BCI | GQ981880 | GQ982100 | GQ982368 |
| Spachea membranacea | Cuatrec. | Malpighiaceae | BCI | GQ981881 | GQ982101 | GQ982369 |
| Spathodea campanulata | P.Beauv. | Bignoniaceae | Luquillo | HM446873 | HM446746 | HM447003 |
| Spiraea chamaedryfolia | L. | Rosaceae | Changbaishan | CANGB046-14 | n/a | n/a |
| Spondias mombin | L. | Anacardiaceae | BCI | GQ981882 | n/a | GQ982370 |
| Spondias radlkoferi | Donn.Sm. | Anacardiaceae | BCI | GQ981883 | n/a | GQ982371 |
| Stemmadenia grandiflora | (Jacq.)Miers | Apocynaceae | BCI | GQ981884 | GQ982102 | GQ982372 |
| Stemonurus malaccensis | (Mast.) Sleumer | Stemonuraceae | Bukit-Timah | BTSIN154-13 | n/a | BTSIN154-13 |
| Sterculia apetala | (Jacq.)H.Karst. | Malvaceae | BCI | GQ981885 | GQ982103 | GQ982373 |
| Sterculia coccinea | Roxb. | Malvaceae | Bukit-Timah | KJ594892 | KJ709089 | BTSIN477-13 |
| Sterculia lanceolata | Cav. | Malvaceae | Dinghushan | HQ415135 | HQ415311 | HQ415485 |
| Sterculia parviflora | Roxb. | Malvaceae | Bukit-Timah | KJ594896 | KJ709092 | BTSIN289-13 |
| Sterculia rubiginosa | Vent. | Malvaceae | Bukit-Timah | KJ594897 | KJ709093 | n/a |
| Stereospermum fimbriatum | DC. | Bignoniaceae | Bukit-Timah | n/a | KJ709094 | BTSIN584-13 |
| Streblus elongatus | (Miq.) Corner | Moraceae | Bukit-Timah | BTSIN085-13 | n/a | BTSIN085-13 |
| Strombosia ceylanica | Gardner | Olacaceae | Bukit-Timah | KJ594898 | KJ709095 | n/a |
| Strombosia javanica | Blume | Olacaceae | Bukit-Timah | KJ594899 | KJ709096 | BTSIN029-13 |
| Stylogyne turbacensis | (Kunth)Mez | Primulaceae | BCI | GQ981886 | GQ982104 | GQ982374 |
| Styrax dasyanthus | Perkins | Styracaceae | Gutianshan | HQ427123 | HQ427280 | n/a |
| Styrax formosanus | Matsum. | Styracaceae | Fushan | KJ688573 | NA | KJ687175 |
| Styrax odoratissimus | Champ. ex Benth. | Styracaceae | Gutianshan | HQ427125 | HQ427282 | n/a |
| Styrax suberifolius | Hook. & Arn. | Styracaceae | Dinghushan | HQ427124 | HQ427281 | HQ415402 |
| Swartzia simplex | Spreng. | Fabaceae | BCI | GQ981887 | GQ982105 | n/a |
| Swartzia simplex var grandiflora | Spreng. | Fabaceae | BCI | GQ981887 | GQ982105 | n/a |
| Swartzia simplex var ochnacea | Spreng. | Fabaceae | BCI | GQ981887 | GQ982105 | n/a |
| Swietenia macrophylla | King | Meliaceae | Luquillo | HM446874 | HM446747 | HM447004 |
| Swintonia schwenckei | Teijsm. & Binn. | Anacardiaceae | Bukit-Timah | KJ594901 | n/a | BTSIN436-13 |
| Symphonia globulifera | L.f. | Clusiaceae | BCI | GQ981889 | n/a | GQ982376 |
| Symplocos adenopus | Hance | Symplocaceae | Dinghushan | HQ415169 | HQ415340 | HQ415518 |
| Symplocos anomala | Brand | Symplocaceae | Gutianshan | HQ427233 | n/a | n/a |
| Symplocos caudata | Wall. Ex G.Don | Symplocaceae | Fushan | KJ688586 | KJ687836 | KJ687188 |
| Symplocos cochinchinensis | (Lour.) S. Moore | Symplocaceae | Dinghushan | HQ415170 | HQ415341 | HQ415519 |
| Symplocos glauca | (Thunb.) Koidz. | Symplocaceae | Fushan | KJ688592 | KJ687842 | KJ687194 |
| Symplocos heishanensis | Hayata | Symplocaceae | Fushan | KJ688599 | NA | KJ687201 |
| Symplocos lancifolia | Siebold & Zucc. | Symplocaceae | Dinghushan | HQ415167 | HQ415339 | HQ415516 |
| Symplocos martinicensis | Jacq. | Symplocaceae | Luquillo | HM446875 | n/a | HM447005 |
| Symplocos morrisonicola | Hayata | Symplocaceae | Lienhuachih | KJ688604 | KJ687849 | KJ687204 |
| Symplocos paniculata | Miq. | Symplocaceae | Gutianshan | HQ427234 | HQ427378 | n/a |
| Symplocos setchuensis | Brand | Symplocaceae | Fushan | KJ688608 | KJ687853 | KJ687208 |
| Symplocos sonoharae | Koidz. | Symplocaceae | Fushan | KJ688613 | NA | KJ687213 |
| Symplocos stellaris | Brand | Symplocaceae | Gutianshan | HQ427236 | HQ427379 | n/a |
| Symplocos sumuntia | Buch.-Ham. ex D. Don | Symplocaceae | Gutianshan | HQ427232 | HQ427377 | n/a |
| Symplocos wikstroemiifolia | Hayata | Symplocaceae | Fushan | KJ688623 | KJ687866 | KJ687222 |
| Syringa reticulata subsp amurensis | (Rupr.) P.S.Green & M.C.Chang | Oleaceae | Changbaishan | CANGB047-14 | n/a | n/a |
| Syringa wolfii | C.K. Schneid. | Oleaceae | Changbaishan | CANGB048-14 | n/a | n/a |
| Syzygium acuminatissimum | (Blume) DC. | Myrtaceae | Dinghushan | HQ415209.1 | HQ415374.1 | HQ415556.1 |
| Syzygium brittle | none | Myrtaceae | Bukit-Timah | n/a | KJ709097 | n/a |
| Syzygium buxifolirm | n/a | Myrtaceae | Gutianshan | HQ427244.1 | n/a | n/a |
| Syzygium championii | (Benth.) Merr. & L.M. Perry | Myrtaceae | Dinghushan | HQ415142 | HQ415318 | HQ415492 |
| Syzygium duthieanum | (King) Masam. | Myrtaceae | Bukit-Timah | KJ594903 | KJ709098 | n/a |
| Syzygium filiforme kilat | (Wall. ex Duthie) Chantaran. & J. Parn. | Myrtaceae | Bukit-Timah | n/a | KJ709099 | BTSIN620-13 |
| Syzygium formosanum | (Hayata) | Myrtaceae | Fushan | KJ688630 | NA | NA |
| Syzygium grande | (Wight) Walp. | Myrtaceae | Bukit-Timah | n/a | KJ709101 | n/a |
| Syzygium hancei | Merr. & L.M. Perry | Myrtaceae | Dinghushan | HQ415140 | HQ415316 | HQ415490 |
| Syzygium jambos | (L.)Alston | Myrtaceae | Luquillo | HM446876 | HM446748 | HM447006 |
| Syzygium levinei | (Merr.) Merr. & L.M. Perry | Myrtaceae | Dinghushan | HQ415137 | HQ415313 | HQ415487 |
| Syzygium linocieroideum | (King) I.M.Turner | Myrtaceae | Bukit-Timah | BTSIN633-13 | n/a | BTSIN633-13 |
| Syzygium ngadimanianum | (M.R. Hend.) I.M.Turner | Myrtaceae | Bukit-Timah | KJ594904 | KJ709102 | BTSIN113-13 |
| Syzygium nigricans | (King) Merr. & L.M. Perry | Myrtaceae | Bukit-Timah | KJ594909 | KJ709107 | n/a |
| Syzygium oblongifolium | Merr. | Myrtaceae | Bukit-Timah | n/a | n/a | n/a |
| Syzygium palembanicum | Miq. | Myrtaceae | Bukit-Timah | n/a | KJ709108 | n/a |
| Syzygium pauper | (Ridl.) I.M.Turner | Myrtaceae | Bukit-Timah | n/a | KJ709109 | n/a |
| Syzygium pendens | (Duthie) I.M.Turner | Myrtaceae | Bukit-Timah | n/a | KJ709110 | n/a |
| Syzygium pseudoformosum | (King) Merr. & L.M. Perry | Myrtaceae | Bukit-Timah | KJ594910 | KJ709111 | BTSIN422-13 |
| Syzygium pustulatum | (Duthie) Merr. | Myrtaceae | Bukit-Timah | KJ594911 | KJ709114 | BTSIN135-13 |
| Syzygium rehderianum | Merr. & L.M. Perry | Myrtaceae | Dinghushan | HQ415139 | HQ415315 | HQ415489 |
| Syzygium ridleyi | (King) Chantaran. & J.A.N. Parnell | Myrtaceae | Bukit-Timah | KJ594912 | KJ709116 | n/a |
| Syzygium singaporense | (King) Airy Shaw | Myrtaceae | Bukit-Timah | KJ594913 | KJ709117 | n/a |
| Syzygium subdecussatum | (Duthie) I.M.Turner | Myrtaceae | Bukit-Timah | n/a | KJ709118 | BTSIN123-13 |
| Syzygium zeylanicum | (L.) DC. | Myrtaceae | Bukit-Timah | KJ594914 | n/a | n/a |
| Tabebuia guayacan | (Seem.)Hemsl. | Bignoniaceae | BCI | GQ981890 | GQ982107 | GQ982377 |
| Tabebuia heterophylla | (DC.) Britton | Bignoniaceae | Luquillo | HM446877 | n/a | HM447007 |
| Tabebuia rosea | (Bertol.)A.DC. | Bignoniaceae | BCI | GQ981891 | GQ982108 | GQ982378 |
| Tabernaemontana arborea | Rose | Apocynaceae | BCI | GQ981892 | GQ982109 | GQ982379 |
| Tachigali versicolor | Standl.&L.O.Williams | Fabaceae | BCI | GQ981893 | n/a | GQ982380 |
| Talisia croatii | Acev.-Rodr. | Sapindaceae | BCI | GQ981894 | n/a | GQ982381 |
| Talisia nervosa | Radlk. | Sapindaceae | BCI | AJ403008.1 | EU720643.1 | EU720643.1 |
| Tarenna costata | (Miq.) Merr. | Rubiaceae | Bukit-Timah | KJ594915 | KJ709119 | BTSIN429-13 |
| Tarenna mollissima | (Hook. & Arn.) B.L. Rob. | Rubiaceae | Dinghushan | HQ427200 | HQ427349 | HQ415548 |
| Taxus baccata | L. | Taxaceae | Wytham | NC_020321 | NC_020321 | NC_020321 |
| Taxus brevifolia | Nutt. | Taxaceae | Wind_River | AF249666.1 | n/a | n/a |
| Teijsmanniodendron coriaceum | Kosterm. | Lamiaceae | Bukit-Timah | KJ594916 | KJ709120 | BTSIN120-13 |
| Terminalia amazonia | (J.F.Gmel.)Exell | Combretaceae | BCI | GQ981895 | n/a | GQ982382 |
| Terminalia citrina | Roxb. ex Fleming | Combretaceae | Bukit-Timah | n/a | n/a | n/a |
| Terminalia oblonga | (Ruiz&Pav.)Steud. | Combretaceae | BCI | GQ981896 | n/a | GQ982383 |
| Ternstroemia gymnanthera | (Wight & Arn.) Sprague | Pentaphylacaceae | Lienhuachih | KJ688633 | KJ687870 | KJ687229 |
| Ternstroemia luquillensis | Krug & Urb. | Theaceae | Luquillo | KJ082611 | KJ012802 | KJ426968 |
| Tetradium glabrifolium | (Champ.ex Benth.) T.G. Hartley | Rutaceae | Fushan | KJ688636 | KJ687871 | KJ687232 |
| Tetragastris balsamifera | Oken | Burseraceae | Luquillo | HM446878 | HM446749 | HM447008 |
| Tetragastris panamensis | (Engl.)Kuntze | Burseraceae | BCI | GQ428579.1 | n/a | GQ982384 |
| Tetrathylacium johansenii | Standl. | Salicaceae | BCI | GQ981898 | GQ982110 | GQ982385 |
| Theobroma cacao | L. | Malvaceae | BCI | GQ981897 | GQ982111 | GQ982386 |
| Thevetia ahouai | (L.)A.DC. | Apocynaceae | BCI | GQ981899 | GQ982112 | GQ982387 |
| Thuja occidentalis | L. | Cupressaceae | Wabikon Lake | KJ593720 | n/a | n/a |
| Thuja plicata | Donn ex D. Don | Cupressaceae | Wind_River | AF127428.2 | n/a | n/a |
| Tilia americana | L. | Malvaceae | Wabikon Lake | KJ593722 | KJ593130 | n/a |
| Tilia amurensis | Rupr. | Malvaceae | Changbaishan | CANGB049-14 | n/a | n/a |
| Tilia endochrysea | Hand.-Mazz. | Malvaceae | Gutianshan | HQ427156 | HQ427306 | n/a |
| Tilia mandshurica | Rupr. & Maxim. | Malvaceae | Changbaishan | CANGB050-14 | n/a | n/a |
| Timonius wallichianus | (Korth.) Valeton | Rubiaceae | Bukit-Timah | KJ594918 | KJ709122 | BTSIN342-13 |
| Tocoyena pittieri | (Standl.)Standl. | Rubiaceae | BCI | GQ981900 | GQ982113 | GQ982388 |
| Toxicodendron succedaneum | (L.) Kuntze | Anacardiaceae | Dinghushan | HQ427194 | HQ427343 | n/a |
| Toxicodendron sylvestre | (Siebold & Zucc.) Kuntze | Anacardiaceae | Dinghushan | HQ415143 | HQ415319 | HQ415493 |
| Trattinnickia aspera | Kunth | Burseraceae | BCI | GQ981901.1 | GQ982114.1 | GQ982389.1 |
| Trema cannabina | Lour. | Rosaceae | Lienhuachih | KJ688640 | KJ687875 | KJ687238 |
| Trema micrantha | (L.)Blume | Ulmaceae | Luquillo | KJ082620 | KJ012809 | n/a |
| Trema orientalis | (L.) Blume | Cannabaceae | Lienhuachih | KJ688644 | KJ687879 | KJ687242 |
| Trema tomentosa | (Roxb.) H. Hara | Ulmaceae | Dinghushan | HQ415174 | HQ415345 | HQ415523 |
| Triadica cochinchinensis | Lour. | Euphorbiaceae | Dinghushan | HQ415199.1 | HQ415366.1 | HQ415547.1 |
| Tricalysia dubia | (Lindl.) Ohwi | Rubiaceae | Fushan | KJ688647 | KJ687881 | KJ687244 |
| Trichanthera gigantea | (Bonpl.) Nees | Acanthaceae | BCI | GQ981903 | GQ982116 | GQ982391 |
| Trichilia pallida | Sw. | Meliaceae | Luquillo | HM446879 | HM446750 | HM447009 |
| Trichilia tuberculata | (Triana&Planch.)C.DC. | Meliaceae | BCI | GQ981905 | n/a | GQ982393 |
| Trichospermum galeottii | (Turcz.)Kosterm. | Malvaceae | BCI | JQ594273.1 | n/a | n/a |
| Trigonostemon longifolius | Baill. | Euphorbiaceae | Bukit-Timah | KJ594921 | KJ709124 | n/a |
| Triomma malaccensis | Hook. f. | Burseraceae | Bukit-Timah | KJ594922 | KJ709125 | n/a |
| Triplaris cumingiana | Fisch.&C.A.Mey.exC.A.Mey. | Polygonaceae | BCI | GQ981906 | GQ982118 | GQ982394 |
| Trophis caucana | (Pittier)C.C.Berg | Moraceae | BCI | GQ981907 | GQ982119 | GQ982395 |
| Trophis racemosa | (L.)Urb. | Moraceae | BCI | GQ981908 | GQ982120 | GQ982396 |
| Tsuga canadensis | (L.) CarriËre | Pinaceae | Wabikon Lake | KJ593735 | n/a | n/a |
| Turpinia formosana | Nakai | Staphyleaceae | Fushan | KJ688656 | KJ687890 | KJ687253 |
| Turpinia occidentalis | (Sw.)G.Don | Staphyleaceae | Luquillo | HM446880 | HM446751 | HM447010 |
| Tutcheria microcarpa | Dunn | Theaceae | Gutianshan | HQ427231 | HQ427376 | n/a |
| Ulmus americana | L. | Ulmaceae | Wabikon Lake | KJ593739 | KJ593141 | n/a |
| Ulmus davidiana | Planch. | Ulmaceae | Changbaishan | CANGB051-14 | n/a | n/a |
| Ulmus laciniata | (Trautv.) Mayr | Ulmaceae | Changbaishan | CANGB052-14 | n/a | n/a |
| Ulmus rubra | Muhl. | Ulmaceae | SCBI | KC539680.1 | KC539611.1 | n/a |
| Unonopsis pittieri | Saff. | Annonaceae | BCI | GQ981910 | GQ982122 | GQ982398 |
| Urera baccifera | (L.)Gaudich.exWedd. | Urticaceae | Luquillo | HM446881 | HM446752 | HM447011 |
| Urophyllum blumeanum | (Wight) Hook. f. | Rubiaceae | Bukit-Timah | KJ594923 | KJ709126 | BTSIN203-13 |
| Urophyllum glabrum | Jack ex Wall. | Rubiaceae | Bukit-Timah | KJ594924 | KJ709127 | BTSIN206-13 |
| Urophyllum hirsutum | (Wight) Hook.f. | Rubiaceae | Bukit-Timah | KJ594925 | n/a | n/a |
| Vaccinium bracteatum | Thunb. | Ericaceae | Fushan | KJ688660 | NA | KJ687256 |
| Vaccinium randaiense | Hayata | Ericaceae | Lienhuachih | KJ688664 | NA | KJ687261 |
| Vachellia melanoceras | Beurl. | Fabaceae | BCI | GQ981912 | GQ982124 | GQ982400 |
| Vatica maingayi | Dyer | Dipterocarpaceae | Bukit-Timah | KJ594927 | KJ709130 | BTSIN044-13 |
| Vatica odorata | Symington | Dipterocarpaceae | Bukit-Timah | n/a | KJ709131 | BTSIN624-13 |
| Vatica ridleyana | Brandis | Dipterocarpaceae | Bukit-Timah | KJ594928 | KJ709132 | BTSIN416-13 |
| Verbesina gigantea | Jacq. | Asteraceae | BCI | JQ590731.1 | JQ586939.1 | n/a |
| Vernicia montana | Lour. | Euphorbiaceae | Gutianshan | HQ427131 | HQ427287 | n/a |
| Viburnum burejaeticum | Regel & Herd. | Caprifoliaceae | Changbaishan | CANGB053-14 | n/a | n/a |
| Viburnum erosum | Thunb. | Caprifoliaceae | Gutianshan | HQ427216 | HQ427362 | n/a |
| Viburnum formosanum | (Hance) Hayata | Adoxaceae | Fushan | KJ688668 | NA | KJ687265 |
| Viburnum luzonicum | Rolfe | Adoxaceae | Lienhuachih | KJ688669 | NA | KJ687266 |
| Viburnum odoratissimum | Ker Gawl. | Adoxaceae | Dinghushan | HQ415114 | HQ415292 | HQ415464 |
| Viburnum prunifolium | L. | Adoxaceae | SCBI | DQ006080.1 | n/a | n/a |
| Viburnum sargentii | Koehne | Adoxaceae | Changbaishan | CANGB054-14 | n/a | n/a |
| Viburnum sempervirens | K. Koch | Adoxaceae | Gutianshan | HQ427217.1 | n/a | n/a |
| Viburnum_acerifolium | L. | Adoxaceae | SCBI | DQ006079.1 | n/a | DQ006166.1 |
| Virola multiflora | (Standl.)A.C.Sm. | Myristicaceae | BCI | GQ981913 | GQ982125 | GQ982401 |
| Virola nobilis | A.C. Sm. | Myristicaceae | BCI | GQ981914 | GQ982126 | GQ982402 |
| Virola sebifera | Aubl. | Myristicaceae | BCI | GQ981915 | n/a | n/a |
| Vismia baccifera | (L.)Triana&Planch. | Hypericaceae | BCI | GQ981916 | n/a | GQ982403 |
| Vismia billbergiana | Beurl. | Hypericaceae | BCI | GQ981917 | GQ982127 | GQ982404 |
| Vitex divaricata | Sw. | Verbenaceae | Luquillo | U78716 | n/a | HM447012 |
| Vitex quinata | (Lour.) F.N. Williams | Verbenaceae | Dinghushan | HQ415126 | HQ415304 | HQ415476 |
| Vochysia ferruginea | Mart. | Vochysiaceae | BCI | GQ981918 | GQ982128 | GQ982405 |
| Walsura chrysogyne | (Miq.) Bakh.f.apud van Steenis | Meliaceae | Bukit-Timah | KJ594932 | KJ709133 | BTSIN450-13 |
| Weigela japonica | Thunb. | Caprifoliaceae | Gutianshan | HQ427218.1 | HQ427364.1 | n/a |
| Wendlandia formosana | Cowan | Rubiaceae | Fushan | KJ688671 | NA | KJ687269 |
| Wendlandia uvariifolia | Hance | Rubiaceae | Lienhuachih | KJ688677 | NA | KJ687277 |
| Wikstroemia indica | (L.) C.A. Mey. | Thymelaeaceae | Dinghushan | HQ415147 | HQ415322 | HQ415497 |
| Wikstroemia monnula | Hance | Thymelaeaceae | Gutianshan | HQ427215 | n/a | n/a |
| Wikstroemia nutans | Champ. ex Benth. | Thymelaeaceae | Dinghushan | HQ415148 | n/a | HQ415498 |
| Xanthophyllum amoenum | Chodat | Polygalaceae | Bukit-Timah | BTSIN557-13 | n/a | BTSIN557-13 |
| Xanthophyllum discolor | Chodat | Polygalaceae | Bukit-Timah | KJ594934 | KJ709134 | BTSIN311-13 |
| Xanthophyllum ellipticum | Korth. ex Miq. | Polygalaceae | Bukit-Timah | BTSIN141-13 | n/a | BTSIN141-13 |
| Xanthophyllum eurhynchum | Miq. | Polygalaceae | Bukit-Timah | KJ594935 | KJ709135 | BTSIN310-13 |
| Xanthophyllum griffithii | Hook.f. ex A.W.Benn. | Polygalaceae | Bukit-Timah | KJ594936 | n/a | BTSIN350-13 |
| Xanthophyllum hainanense | Hu | Polygalaceae | Dinghushan | HQ415112 | HQ415290 | HQ415462 |
| Xerospermum noronhianum | (Blume) Blume | Sapindaceae | Bukit-Timah | KJ594938 | KJ709136 | BTSIN062-13 |
| Xylopia caudata | Hook. f. & Thomson | Annonaceae | Bukit-Timah | KJ594940 | KJ709137 | BTSIN464-13 |
| Xylopia macrantha | Triana&Planch. | Annonaceae | BCI | n/a | n/a | GQ982406 |
| Xylopia malayana | Hook. f. & Thomson | Annonaceae | Bukit-Timah | BTSIN190-13 | n/a | BTSIN190-13 |
| Xylosma oligandra | Donn.Sm. | Salicaceae | BCI | GQ981919 | GQ982129 | GQ982407 |
| Xylosma racemosa | (Siebold & Zucc.) Miq. | Salicaceae | Gutianshan | HQ427273.1 | n/a | n/a |
| Xylosma schwaneckeana | (Krug & Urb.) Urb. | Salicaceae | Luquillo | HM446882 | HM446753 | HM447013 |
| Zanthoxylum ailanthoides | Siebold & Zucc. | Rutaceae | Lienhuachih | KJ688678 | KJ687897 | KJ687278 |
| Zanthoxylum avicennae | (Lam.) DC. | Rutaceae | Dinghushan | HQ415109 | HQ415288 | HQ415459 |
| Zanthoxylum ekmanii | (Urb.)Alain | Rutaceae | BCI | GQ981920 | GQ982130 | GQ982408 |
| Zanthoxylum juniperinum | Poepp. | Rutaceae | BCI | GQ981921 | n/a | GQ982409 |
| Zanthoxylum martinicense | (Lam.) DC. | Rutaceae | Luquillo | HM446883 | HM446754 | HM447014 |
| Zanthoxylum myriacanthum | Wall. ex Hook. f. | Rutaceae | Dinghushan | HQ415108 | HQ415287 | HQ415458 |
| Zanthoxylum panamense | P.Wilson | Rutaceae | BCI | n/a | GQ981922 | GQ982410 |
| Zanthoxylum setulosum | P.Wilson | Rutaceae | BCI | GQ981923 | GQ982131 | GQ982411 |
| Zuelania guidonia | (Sw.)Britton&Millsp. | Salicaceae | BCI | GQ981924 | n/a | GQ982412 |
