# Supplementary material for: Comparative evolutionary diversity and phylogenetic structure across multiple forest dynamics plots: a mega-phylogeny approach
Source: Front Genet. 2014 Nov 5;5:358. doi: 10.3389/fgene.2014.00358 (PMC4220724; doi:10.3389/fgene.2014.00358)
Supplement: Supplementary file 2 [file Table2.DOCX]

Supplemental Table S2. Configuration parameters for GARLI ML tree search

[general]

searchreps=1

streefname = random

constraintfile = constraint.txt

subsetspecificrates = 0

linkmodels =0

saveevery = 10000

enforcetermconditions = 1

genthreshfortopoterm = 10000

significanttopochange = 0.02

scorethreshforterm = 0.01

logevery = 1000

restart = 0

datafname = infile

availablememory = 5000

outputphyliptree = 0

outputeachbettertopology = 0

writecheckpoints = 0

outputmostlyuselessfiles = 0

refinestart = 1

randseed = -1

ofprefix = garli_run

collapsebranches = 1

[model1]

ratematrix = 6rate

datatype = nucleotide

numratecats = 4

ratehetmodel = gamma

invariantsites = estimate

statefrequencies = estimate

numratecats=4

[model2]

ratematrix = 6rate

datatype = nucleotide

numratecats = 4

ratehetmodel = gamma

invariantsites = estimate

statefrequencies = estimate

numratecats=4

[model3]

ratematrix = 6rate

datatype = nucleotide

numratecats = 4

ratehetmodel = gamma

invariantsites = estimate

statefrequencies = estimate

numratecats=4

[master]

gammashapebrlen = 1000

modweight = 0.05

brlenweight = 0.2

topoweight = 1.0

randnniweight = 0.1

randsprweight = 0.3

limsprweight = 0.6

stopgen = 814783646

stoptime = 814783646

resampleproportion = 1.0

bootstrapreps=0

startoptprec = 0.5

minoptprec = 0.01

numberofprecreductions = 20

gammashapemodel = 1000

selectionintensity = 0.5

nindivs = 4

holdover = 1

intervalstostore = 5

meanbrlenmuts = 5

treerejectionthreshold = 50.0

intervallength = 100

inferinternalstateprobs = 0

holdoverpenalty = 0

limsprrange= 6

uniqueswapbias = 0.1

distanceswapbias = 1.0

Estimated model parameters for the data partitions (by gene, named below) inferred by GARLI for the ML phylogeny used in this study for the mega-phylogeny.

Model 1 (=rbcL)

Number of states = 4 (nucleotide data)

Nucleotide Relative Rate Matrix: 6 rates

AC = 1.000, AG = 4.000, AT = 1.000, CG = 1.000, CT = 4.000, GT = 1.000

Equilibrium State Frequencies: estimated

(ACGT) 0.2708 0.2157 0.2241 0.2895

Rate Heterogeneity Model:

4 discrete gamma distributed rate categories, alpha param estimated

0.5000

Substitution rate categories under this model:

rate proportion

0.0334 0.2500

0.2519 0.2500

0.8203 0.2500

2.8944 0.2500

Model 2 = (matK)

Number of states = 4 (nucleotide data)

Nucleotide Relative Rate Matrix: 6 rates

AC = 1.000, AG = 4.000, AT = 1.000, CG = 1.000, CT = 4.000, GT = 1.000

Equilibrium State Frequencies: estimated

(ACGT) 0.2936 0.1848 0.1596 0.3621

Rate Heterogeneity Model:

4 discrete gamma distributed rate categories, alpha param estimated

0.5000

Substitution rate categories under this model:

rate proportion

0.0334 0.2500

0.2519 0.2500

0.8203 0.2500

2.8944 0.2500

Model 3 ( =psbA-trnH)

Number of states = 4 (nucleotide data)

Nucleotide Relative Rate Matrix: 6 rates

AC = 1.000, AG = 4.000, AT = 1.000, CG = 1.000, CT = 4.000, GT = 1.000

Equilibrium State Frequencies: estimated

(ACGT) 0.3375 0.1136 0.1567 0.3922

Rate Heterogeneity Model:

4 discrete gamma distributed rate categories, alpha param estimated

0.5000

Substitution rate categories under this model:

rate proportion

0.0334 0.2500

0.2519 0.2500

0.8203 0.2500

2.8944 0.2500
