# Supplementary material for: Comparative evolutionary diversity and phylogenetic structure across multiple forest dynamics plots: a mega-phylogeny approach
Source: Front Genet. 2014 Nov 5;5:358. doi: 10.3389/fgene.2014.00358 (PMC4220724; doi:10.3389/fgene.2014.00358)
Supplement: Supplementary file 3 [file Table3.DOCX]

Supplemental Table S3. Standard effects sizes for PD randomization

| Plot | ntaxa | pd.obs | pd.rand.mean | pd.rand.sd | pd.obs.rank | pd.obs.z | pd.obs.p | runs |
| --- | --- | --- | --- | --- | --- | --- | --- | --- |
|  |  |  |  |  |  |  |  |  |
| BCI | 337 | 28.883 | 32.481 | 1.486 | 13.000 | -2.420 | 0.013 | 999 |
| BT | 326 | 25.805 | 31.885 | 1.486 | 1.000 | -4.091 | 0.001 | 999 |
| Dinghushan | 192 | 18.557 | 23.370 | 1.440 | 2.000 | -3.342 | 0.002 | 999 |
| Gutianshan | 146 | 16.102 | 19.612 | 1.454 | 2.000 | -2.414 | 0.002 | 999 |
| Luquillo | 141 | 19.572 | 19.280 | 1.460 | 561.000 | 0.200 | 0.561 | 999 |
| Lienhuachih | 129 | 14.176 | 18.210 | 1.461 | 1.000 | -2.760 | 0.001 | 999 |
| Fushan | 98 | 12.676 | 15.261 | 1.343 | 6.000 | -1.925 | 0.006 | 999 |
| SCBI | 62 | 8.670 | 11.229 | 1.235 | 3.000 | -2.071 | 0.003 | 999 |
| Changbai | 54 | 7.155 | 10.215 | 1.210 | 1.000 | -2.529 | 0.001 | 999 |
| Nanjenshan | 42 | 8.269 | 8.510 | 1.043 | 465.000 | -0.231 | 0.465 | 999 |
| SERC | 30 | 6.192 | 6.687 | 0.979 | 349.000 | -0.505 | 0.349 | 999 |
| Wabikon_Lake | 28 | 5.631 | 6.349 | 0.947 | 205.000 | -0.758 | 0.205 | 999 |
| Wytham | 18 | 4.384 | 4.557 | 0.828 | 495.000 | -0.209 | 0.495 | 999 |
| Wind_River | 7 | 3.126 | 2.070 | 0.479 | 979.000 | 2.205 | 0.979 | 999 |
| Yosemite | 7 | 2.995 | 2.106 | 0.549 | 962.000 | 1.621 | 0.962 | 999 |

Supplemental Table S4. Standard effects sizes for MPD randomization.

| Plot | ntaxa | mpd.obs | mpd.rand.mean | mpd.rand.sd | mpd.obs.rank | mpd.obs.z | mpd.obs.p | runs |
| --- | --- | --- | --- | --- | --- | --- | --- | --- |
|  |  |  |  |  |  |  |  |  |
| BCI | 337 | 0.611 | 0.658 | 0.018 | 6.000 | -2.535 | 0.006 | 999 |
| BT | 326 | 0.600 | 0.657 | 0.020 | 1.000 | -2.913 | 0.001 | 999 |
| Dinghushan | 192 | 0.608 | 0.656 | 0.027 | 19.000 | -1.781 | 0.019 | 999 |
| Gutianshan | 146 | 0.602 | 0.657 | 0.031 | 20.000 | -1.749 | 0.020 | 999 |
| Luquillo | 141 | 0.671 | 0.657 | 0.030 | 693.000 | 0.468 | 0.693 | 999 |
| Lienhuachih | 129 | 0.615 | 0.659 | 0.035 | 105.000 | -1.248 | 0.105 | 999 |
| Fushan | 98 | 0.716 | 0.656 | 0.039 | 918.000 | 1.525 | 0.918 | 999 |
| SCBI | 62 | 0.659 | 0.658 | 0.050 | 570.000 | 0.031 | 0.570 | 999 |
| Changbai | 54 | 0.692 | 0.656 | 0.053 | 764.000 | 0.670 | 0.764 | 999 |
| Nanjenshan | 42 | 0.594 | 0.657 | 0.062 | 117.000 | -1.013 | 0.117 | 999 |
| SERC | 30 | 0.750 | 0.660 | 0.075 | 862.000 | 1.193 | 0.862 | 999 |
| Wabikon_Lake | 28 | 0.781 | 0.659 | 0.076 | 916.000 | 1.617 | 0.916 | 999 |
| Wytham | 18 | 0.787 | 0.654 | 0.089 | 916.000 | 1.506 | 0.916 | 999 |
| Wind_River | 7 | 0.924 | 0.658 | 0.146 | 966.000 | 1.821 | 0.966 | 999 |
| Yosemite | 7 | 0.859 | 0.655 | 0.156 | 936.000 | 1.306 | 0.936 | 999 |

Supplemental Table S5: Standard effects sizes for MNTD randomization.

| Plot | ntaxa | mntd.obs | mntd.rand.mean | mntd.rand.sd | mntd.obs.rank | mntd.obs.z | mntd.obs.p | runs |
| --- | --- | --- | --- | --- | --- | --- | --- | --- |
|  |  |  |  |  |  |  |  |  |
| BCI | 337 | 0.095 | 0.102 | 0.007 | 153.000 | -1.001 | 0.153 | 999 |
| Bukit-Timah | 326 | 0.079 | 0.104 | 0.008 | 1.000 | -3.138 | 0.001 | 999 |
| Dinghushan | 192 | 0.088 | 0.137 | 0.013 | 1.000 | -3.895 | 0.001 | 999 |
| Gutianshan | 146 | 0.119 | 0.157 | 0.015 | 3.000 | -2.490 | 0.003 | 999 |
| Luquillo | 141 | 0.143 | 0.160 | 0.016 | 153.000 | -1.052 | 0.153 | 999 |
| Lienhuachih | 129 | 0.110 | 0.166 | 0.018 | 1.000 | -3.203 | 0.001 | 999 |
| Fushan | 98 | 0.116 | 0.190 | 0.022 | 1.000 | -3.414 | 0.001 | 999 |
| SCBI | 62 | 0.131 | 0.233 | 0.030 | 1.000 | -3.402 | 0.001 | 999 |
| Changbaishan | 54 | 0.104 | 0.247 | 0.033 | 1.000 | -4.318 | 0.001 | 999 |
| Nanjenshan | 42 | 0.232 | 0.275 | 0.042 | 145.000 | -1.008 | 0.145 | 999 |
| SERC | 30 | 0.192 | 0.309 | 0.053 | 4.000 | -2.223 | 0.004 | 999 |
| Wabikon_Lake | 28 | 0.175 | 0.319 | 0.056 | 1.000 | -2.559 | 0.001 | 999 |
| Wytham | 18 | 0.172 | 0.367 | 0.069 | 1.000 | -2.828 | 0.001 | 999 |
| Wind_River | 7 | 0.308 | 0.485 | 0.111 | 35.000 | -1.597 | 0.035 | 999 |
| Yosemite | 7 | 0.313 | 0.479 | 0.113 | 43.000 | -1.473 | 0.043 | 999 |
| CTFS megatree | 1347 | 0.042 |  |  |  |  |  |  |
